# Supplementary material for: Exploration of physiological and biochemical processes of canola with exogenously applied fertilizers and plant growth regulators under drought stress
Source: PLoS One. 2021 Dec 20;16(12):e0260960. doi: 10.1371/journal.pone.0260960 (PMC8687561; doi:10.1371/journal.pone.0260960)
Supplement: Attachment — Submitted filename: PONE-D-21-20309_R1.pdf [file pone.0260960.s006.pdf]

PLOS ONE

Physiological and Biochemical Exploration of Canola for Overcoming Yield Limitation by Integrated Fertilizer management under different water regimes

--Manuscript Draft--

**Manuscript Number:**	PONE-D-21-20309R1
**Article Type:**	Research Article
**Full Title:**	Physiological and Biochemical Exploration of Canola for Overcoming Yield Limitation by Integrated Fertilizer management under different water regimes
**Short Title:**	Impact of PGR and Fertilizers on the growth and yield of canola
**Corresponding Author:**	Mohammad Aquil Siddiqui, Ph.d<br>Pakistan Atomic Energy Commission<br>Hyderabad, PAKISTAN
**Keywords:**	Antioxidants; elicitors; mitigation strategies; photosynthesis; water shortage; yield
**Abstract:**	Environmental stressors may modify the nutritional profile and economic value of crops. Chemical fertilizers and phyto-hormones are major assets which can enhance the canola production under stressful conditions. Physio-biochemical responses altered remarkably by drought, and this also varies with the use of nitrogen/phosphorus fertilizers (N/P) and plant growth regulators (PGRs). The key interest of current study was to evaluate nutritional quality and physio-biochemical modulation in Brassica napus L. from early growth to seed stage with chemical fertilizers and PGRs in different water regimes. To monitor responses in canola, two season field experiment was conducted as spilt plot under randomized complete block design (RCBD) with four treatments (Control, Chemical fertilizers [Nitrogen (N) 90 kg/ha), Phosphorus (P) and Potassium (K) 45 kg/ha], PGRs indole acetic acid (IAA) 15g/ha, gibberellic acid (GA 3) 15g/ha and the combination of NPK and PGRs] under different irrigations regimes (60, 100, 120, 150 mm evaporations). The activity of peroxidase, catalase, superoxide dismutase, polyphenol oxidase, soluble sugar, leaf temperature, malondialdehyde and proline contents were elevated, leaf water contents, stomatal conductance, chlorophyll contents, membrane stability index and grain yield of canola were reduced under water stress. The combined application of fertilizers and plant growth regulator (PGR) further increased the antioxidant enzymes activities, soluble sugars, along with recovery of leaf water contents, chlorophyll contents, stomatal conductance, and membrane stability index but decreased the proline contents and leaf temperature at different rate of evaporation. There is positive interaction of applied elicitors to the water stress in canola except leaf area. The outcomes depicted that the combination of NPK with PGRs improved the various morpho-physiological as well as biochemical parameters and reduced the pressure of chemical fertilizers cost about 60%. It also reduced the deleterious effect of water limitation on the physiology and grain yield and oil contents of canola in field.
**Order of Authors:**	Mohammad Aquil Siddiqui, Ph.d
	Muhammad Mahran Aslam
	Fozia Farhat
	Shafquat Yasmeen
	Muhammad Tahir Tahir khan
	Mahboob Ali Sial
	Imtiaz Ahmed Khan
**Response to Reviewers:**	File attached and response the all reviewer comments
**Additional Information:**	
**Question**	**Response**
**Financial Disclosure**	The author(s) received no specific funding for this work.

Powered by Editorial Manager® and ProduXion Manager® from Aries Systems Corporation

Enter a financial disclosure statement that describes the sources of funding for the work included in this submission. Review the submission guidelines for detailed requirements. View published research articles from *PLOS ONE* for specific examples.<br><br>This statement is required for submission and **will appear in the published article** if the submission is accepted. Please make sure it is accurate.<br><br>**Unfunded studies**<br>Enter: *The author(s) received no specific funding for this work.*<br><br>**Funded studies**<br>Enter a statement with the following details:<br>• Initials of the authors who received each award<br>• Grant numbers awarded to each author<br>• The full name of each funder<br>• URL of each funder website<br>• Did the sponsors or funders play any role in the study design, data collection and analysis, decision to publish, or preparation of the manuscript?<br>• **NO** - Include this sentence at the end of your statement: *The funders had no role in study design, data collection and analysis, decision to publish, or preparation of the manuscript.*<br>• **YES** - Specify the role(s) played.<br><br>* typeset	
**Competing Interests**<br><br>Use the instructions below to enter a competing interest statement for this submission. On behalf of all authors, disclose any competing interests that could be perceived to bias this work—acknowledging all financial support and any other relevant financial or non-financial competing interests.<br><br>This statement is required for submission and **will appear in the published article** if	The authors have declared that no competing interests exist.

the submission is accepted. Please make sure it is accurate and that any funding sources listed in your Funding Information later in the submission form are also declared in your Financial Disclosure statement.<br><br>View published research articles from *PLOS ONE* for specific examples.<br><br>**NO authors have competing interests**<br><br>Enter: *The authors have declared that no competing interests exist.*<br><br>**Authors with competing interests**<br><br>Enter competing interest details beginning with this statement:<br><br>*I have read the journal's policy and the authors of this manuscript have the following competing interests: [insert competing interests here]*<br><br>* typeset	
**Ethics Statement**<br><br>Enter an ethics statement for this submission. This statement is required if the study involved:<br><br>• Human participants<br>• Human specimens or tissue<br>• Vertebrate animals or cephalopods<br>• Vertebrate embryos or tissues<br>• Field research<br><br>Write "N/A" if the submission does not require an ethics statement.<br><br>General guidance is provided below. Consult the submission guidelines for detailed instructions. **Make sure that all information entered here is included in the Methods section of the manuscript.**	N/A

**Format for specific study types**<br><br>**Human Subject Research (involving human participants and/or tissue)**<br>• Give the name of the institutional review board or ethics committee that approved the study<br>• Include the approval number and/or a statement indicating approval of this research<br>• Indicate the form of consent obtained (written/oral) or the reason that consent was not obtained (e.g. the data were analyzed anonymously)<br><br>**Animal Research (involving vertebrate animals, embryos or tissues)**<br>• Provide the name of the Institutional Animal Care and Use Committee (IACUC) or other relevant ethics board that reviewed the study protocol, and indicate whether they approved this research or granted a formal waiver of ethical approval<br>• Include an approval number if one was obtained<br>• If the study involved *non-human primates*, add *additional details* about animal welfare and steps taken to ameliorate suffering<br>• If anesthesia, euthanasia, or any kind of animal sacrifice is part of the study, include briefly which substances and/or methods were applied<br><br>**Field Research**<br><br>Include the following details if this study involves the collection of plant, animal, or other materials from a natural setting:<br>• Field permit number<br>• Name of the institution or relevant body that granted permission	
**Data Availability**<br><br>Authors are required to make all data underlying the findings described fully available, without restriction, and from the time of publication. PLOS allows rare exceptions to address legal and ethical concerns. See the PLOS Data Policy and FAQ for detailed information.	Yes - all data are fully available without restriction

A Data Availability Statement describing where the data can be found is required at submission. Your answers to this question constitute the Data Availability Statement and **will be published in the article**, if accepted.<br><br>**Important:** Stating 'data available on request from the author' is not sufficient. If your data are only available upon request, select 'No' for the first question and explain your exceptional situation in the text box.<br><br>Do the authors confirm that all data underlying the findings described in their manuscript are fully available without restriction?	
**Describe where the data may be found in full sentences. If you are copying our sample text, replace any instances of XXX with the appropriate details.**<br><br>• If the data are **held or will be held in a public repository**, include URLs, accession numbers or DOIs. If this information will only be available after acceptance, indicate this by ticking the box below. For example: *All XXX files are available from the XXX database (accession number(s) XXX, XXX.*).<br>• If the data are all contained **within the manuscript and/or Supporting Information files**, enter the following: *All relevant data are within the manuscript and its Supporting Information files.*<br>• If neither of these applies but you are able to provide **details of access elsewhere**, with or without limitations, please do so. For example:<br><br>*Data cannot be shared publicly because of [XXX]. Data are available from the XXX Institutional Data Access / Ethics Committee (contact via XXX) for researchers who meet the criteria for access to confidential data.*<br><br>*The data underlying the results presented in the study are available from (include the name of the third party*	All relevant data are within the manuscript.

Powered by Editorial Manager® and ProduXion Manager® from Aries Systems Corporation

and contact information or URL).<br>• This text is appropriate if the data are owned by a third party and authors do not have permission to share the data.<br><br>* typeset	
Additional data availability information:	Tick here if the URLs/accession numbers/DOIs will be available only after acceptance of the manuscript for publication so that we can ensure their inclusion before publication.

Powered by Editorial Manager® and ProduXion Manager® from Aries Systems Corporation

Manuscript
Click here to access/download;Manuscript;canola-1_PLOS ONE.docx

Physiological and Biochemical Exploration of Canola for Overcoming Yield Limitation by Integrated Fertilizer management under different water regimes

Muhammad Mahran Aslam[a], Fozia Farhat[b], Mohammad Aquil Siddiqui[a*], Shafquat Yasmeen[a], Muhammad Tahir Khan[a], Mahboob Ali Sial[a] and Imtiaz Ahmad Khan[a]

[a]Nuclear Institute of Agriculture (NIA), Tando Jam, 70060, Sindh, Pakistan

[b]Department of Botany, Government College Women University, Faisalabad, Pakistan

[*]Corresponding author: Mohammad Aquil Siddiqui[a]

Corresponding author email: Siddiqui_aquil@yahoo.com

ABSTRACT

Environmental stressors may modify the nutritional profile and economic value of crops. Chemical fertilizers and phyto-hormones are major assets which can enhance the canola production under stressful conditions. Physio-biochemical responses altered remarkably by drought, and this also varies with the use of nitrogen/phosphorus fertilizers (N/P) and plant growth regulators (PGRs). The key interest of current study was to evaluate nutritional quality and physio-biochemical modulation in *Brassica napus* L. from early growth to seed stage with chemical fertilizers and PGRs in different water regimes. To monitor responses in canola, two season field experiment was conducted as spilt plot under randomized complete block design (RCBD) with four treatments (Control, Chemical fertilizers [Nitrogen (N) 90 kg/ha), Phosphorus (P) and Potassium (K) 45 kg/ha], PGRs indole acetic acid (IAA) 15g/ha, gibberellic acid (GA_3) 15g/ha and the combination of NPK and PGRs] under different irrigations regimes (60, 100, 120, 150 mm evaporations). The activity of peroxidase, catalase, superoxide dismutase, polyphenol oxidase, soluble sugar, leaf temperature, malondialdehyde and proline contents were elevated , leaf water contents, stomatal conductance, chlorophyll contents, membrane stability index and grain yield of canola were reduced under water stress. The combined application of fertilizers and plant growth regulator (PGR) further increased the antioxidant enzymes activities, soluble sugars, along with recovery of leaf water contents, chlorophyll contents, stomatal conductance, and membrane stability index but decreased the proline contents and leaf temperature at different rate of evaporation. There is positive interaction of applied elicitors to the water stress in canola except leaf area. The outcomes depicted that the combination of NPK with PGRs improved the various morpho-physiological as well as biochemical parameters and reduced the pressure of chemical fertilizers cost about 60%. It also reduced the deleterious effect of water limitation on the physiology and grain yield and oil contents of canola in field.

Keywords: antioxidants, elicitors, mitigation strategies, photosynthesis, water shortage, yield

1. Introduction

Canola crop can survive under diverse environment conditions due to wide range of variability [1,2]. Globally, it is emerging oil seed crop among the other oil seeds due to low erucic acid and glucosinolates in oil and seed cake respectively [3]. Pakistan facing the low yield of canola due to the inefficient management of macro and micronutrient. Thus, the nutrient management strategy

is quite useful source to optimize the canola yield in the arid and semi-arid region of the country [4]. The drastic change in climate and rapid increase of world population is the threat of sustainable food security. These climate change favors the drought and heat stress that limit the crop productivity and effecting future food security [2]. The rain pattern has also been transformed due to climate shift which boosted the drought stress all over the world which decline the crop productivity by effecting physiology, plant growth and reproduction of plant severely[5].

The plant growth and development mainly restricted by the drought stress via declining turgor pressure of plant cells which caused hindrance in the biochemical and physiological intriguing mechanisms of plants [6]. The drought stress also drastically effect the photosynthesis of the plants [7]. The water deficit condition cause the reduced concentration of intracellular CO_2, chlorophyll destruction, photochemical system disorder and stomatal closure [8]. The plant showed physiological response in the form of reactive oxygen species (ROS) production under drought stress. The excess amount of ROS can damage the cell membrane by elevating the lipid peroxidation[9,10]. The plants mitigate the ROS harmful effect by the activities of enzymatic and non- enzymatic antioxidants[11]. Superoxide dismutase (SOD), peroxidase (POX), catalase (CAT) and polyphenol peroxidase (PPO) are the antioxidant enzymes which diminished the ROS concentration during drought stress [9,12]. The accumulation of osmolytes, proline, soluble sugar, soluble proteins are the non-enzymatic reaction that caused osmatic regulation under water deficient condition stress [13]. The antioxidant activities elevated under stress condition in ajowan and canola plant against ROS [14]. The plants showed increase level of proline, antioxidants including POX. PPO and SOD but reduced MDA level under stress condition [15,16]. The performance of plant declined due to water deficit stress by reducing Chl a, Chl b, Membrane stability index, chlorophyll content index and increasing leaf temperature [9].

Balance application of fertilizer influence the crop yield, quality with soil sustainability [17] . Nitrogen play a pivotal role in plant tissue growth and development, being a part of protein, chlorophyll, nucleotides, protein, amino acid which directly influence the quality and quantity of crop production [18]. The adequate nitrogen supply is important attribute to boost up the canola productivity [19]. Thus, any fluctuation in the soil profile, texture, and moisture content at various critical stage of growth and development may sway the nitrogen use efficiency in canola. Canola crop is very responsive to fertilizer application especially nitrogen, phosphorus and potassium

which significantly effects the growth and yield per ha [20]. It also stimulate the leaf area (LA) development after flowering in canola [21,22].

The management of soil fertility is limiting factor for the sustainable agriculture production [20]. This challenges of soil fertility can be overcome by the optimal application of fertilizers and plant growth regulators [23]. The chemical and bio- fertilizer are very effective in improving the micro and macro nutrient via organic compound degradation and nitrogen fixation [24]. This lead to improve nutrient uptake and reduce impact of drought in crops. This would increase the activities of PPO, CAT and POX which ultimately improve the grain yield under drought stress in response to fertilizer and PGRs [9]. The combined application of chemical fertilizers, bio fertilizers and PGRs enhanced the accumulation of proline, sugar contents and chlorophyll contents [25]. The fertilizers along with plant growth regulators elevated the stomatal conductance, water contents and total chlorophyll content under water limiting conditions [26]. These combined application of nutrients improve the soil fertility under water stress condition [27]. Thus, this manuscript comprised to gauge the morphological, physiological and biochemical rejoinders of canola to assimilate fertilizers management and PGRs with limited supply of water.

2. Materials and Methods

2.1. Experimental design and treatments

The two-year (2018-19 & 2019-2020) field experiment was conducted at experimental field of Nuclear Institute of Agriculture, TandoJam Sindh to investigate the variability in physio-biochemical parameters and yield of canola in reaction to fertilization under excessive rate of water evaporation. The experiment was performed in split plots as random complete block design (RCBD) in triplicate with four irrigation levels.

**Irrigation Levels**	**Treatment with Elicitors**
1_0= 60mm evaporation	T_0 = No treatment
	T_1= N (90 kg/ha),P (45 kg/ha) and K (45 kg/ha)
	T_2 =IAA (15g/ha) and GA_3 (15g/ha)
	T_3 = T_1 + T_2

1_1= 100mm evaporation T_0 = No treatment
T_1= N (90 kg/ha), P (45 kg/ha) and K (45 kg/ha)
T_2 =IAA (15g/ha) and GA_3 (15g/ha)
$T_3 = T_1 + T_2$
1_2= 120mm evaporation T_0 = No treatment
T_1= N (90 kg/ha), P (45 kg/ha) and K (45 kg/ha)
T_2 =IAA (15g/ha) and GA_3 (15g/ha)
$T_3 = T_1 + T_2$
1_3= 150mm evaporation T_0 = No treatment
T_1= N (90 kg/ha), P (45 kg/ha) and K (45 kg/ha)
T_2 =IAA (15g/ha) and GA_3 (15g/ha)
$T_3 = T_1 + T_2$

Weeding operations continued frequently when needed during crop growth and development of surhan-2012 plants in both years.

2.2. Determination of Agronomic and yield related parameters

At vegetative stage, height of ten randomly selected canola plants of each treatment were measured with measuring tape (cm) from ground to the tip of flag leaf and counted number of branches per plant while number of siliqua plant^{-1}, siliqua length (cm) and number of seeds siliqua^{-1} were counted after harvesting. Similarly, recorded the data related to days to flower when plant had 50% flowers and days to maturity at discoloration stage. Grains of canola plants were dried in the sun and recorded 1000 grain weight by using digital balance (Model- Explorer OHAUS). Biological yield was determined after harvesting from 4 rows and calculated as kg ha^{-1}.

2.3. Determination of Quality traits

Oil content were extracted with petroleum ether using soxhlet apparatus. All the dried seed samples were coarsely ground and packed carefully into the thimble for oil extraction. The extraction was performed continuously for three cycles (90-120 min.) and oil productivity was drawn through standard formula. Two significant fatty acids, erucic acid (%) and glusionlate (µmol g^{-1}) were detected through High Performance Liquid Chromatography (HLPC). Oil contents were analysed using Gradient HPLC (Shimadzu, Japan) having LC-10AT, SCTL 10A system controller, SPD-10AR UV-VIS detector at 280 nm with C18 stationary column (Shim-Pack CLC-ODS). Elution was done for 60 min with a flow rate of 1ml/min in a gradient system of two mobile phases A (H_2O_2: AA-94:6, pH 2.27), B (ACN100%)[28]. Moisture contents (%) was determined by the weight of water in a seed.

2.4. Plant nutrient content and uptake (grain)

For determination of phosphorus (P) and potassium (K) contents, seed samples were dried at 70°C for 48h. Dried and powdered grain sample (0.5g) was digested with 20mL concentrated nitric acid (HNO_3) by adopting method of [29]. The samples were place for 3 hours at room temperature. After 3 hours, samples were put on the digestion block at 250° C until the solution became tinted yellow in appearance. The digested solution was diluted with 50mL of distilled water and filtered with whatman No. 42 filter paper. Nitrogen content was examined by kjeldahl apparatus (Jones, 1991) while phosphorus content was determined through spectrophotometer (Model Analytikjena Spekol 1500 Germany) at 430 nm (Shimadzu UV3100, Japan)[30].

The phosphorus (P) contents from digested plant samples was determined by recording optical density at 430 nm with spectrophotometer (Model-Spectronic-21) by [31]. The potassium (K) content in gains was resolute by flame photometer (Model-Flame photometer-400) according to the method suggested by [32]

N uptake (kg ha^{-1}) in grain: N (%) x grain yield (kg ha^{-1}) /100

P uptake (kg ha^{-1}) in grain**:** P (%) x grain yield (kg ha^{-1}) /100

K uptake (kg ha^{-1}) in grain: K (%) x grain yield (kg ha^{-1}) /100

2.5. Determination of Physiological and Biochemical parameters

2.5.1. Chlorophyll Contents

Fresh leaf samples were collected from each treatment and subjected to grinding with 80% acetone. Semi-liquid extract was filtered and centrifuged at 10000rpm for 5minutes [33]. The supernatant was then subjected to spectrophotometer (Model Analytikjena Spekol 1500 Germany)

2.5.2. Leaf water content

Leaf water content was measured by harvesting three leaves per plant from every plot after 45 days of sowing. Fresh leaf sample (FW) were weighed in gram and let them dry at high temperature (80^{0}C) and reweighed as dry weight (DW). Leaf water content (LWC) was calculated by following formula. LWC = [(FW – DW) / (FW)]

2.5.3. Leaf temperature

The leaf temperature (LT) was measured at flowering stage with the help of infrared thermometer (TES- 1327). The leave temperature (^{0}C) was measured by randomly selecting 3 plants of every treatment and replicate. Later, the mean LT was carefully recorded.

2.5.4. Stomatal conductance (g_s)

Portable photosystem (Porometer AP4, Delta-T Devices Ltd., Cambridge, U.K.) was used to measure the stomatal conductance. This data was carefully recorded 60 days after sowing (DAS).

2.5.5. Membrane stability index (MSI)

The previously reported method of Ghassemi-Golezani et al. (2016)[34] with slight modification was used to calculate membrane stability index. About 0.1 g leaf samples was mixed with double distilled water (10 ml) in falcon tube and incubated at 40^{0}C for 30 min and electrical conductivity was measured (EC_1). Thereafter, that conductivity of these sample were assessed after placing at 100^{0}C for 10 min (EC_2). The MSI was measured by the following formulas:

$$\text{Membrane Stability Index (MSI)} = (EC_1 / EC_2) \times 100$$

2.6. Determination of osmolytes

The total soluble sugar content was estimated in canola leaves[35]. The standard calibration curve of pure glucose was used to determine total soluble sugars of leaves and expressed as mg/g DW. To determine proline contents in canola, leaf sample was thoroughly grinded in 3% sulfosalicylic acid. The extracted sample was poured and mixed with glacial acetic acid and ninhydrin in a test tube with a ratio of 1:1:1. These mixture was heated at 100^{0}C for 60 min in a Bain Marie oven. Then reaction mixture was cooled at room temperature and the toluene used for the extraction of mixture, vortexed for 30sec. The absorbance of the upper phase was recorded at 520 nm. Calibration curve of pure proline was used to compare the proline content of canola leaves and expressed as mg/g FW [36].

2.7. Determination of Antioxidants

Young leaves were collected from each treatment at 60 DAS and assayed the activity of polyphenol oxidase (PPO) by Kumar and Khan (1982) method [37]. The reaction mixture contains 0.1 M phosphate buffer, 1 ml catechol and 5 ml enzyme extract. The reaction mixture was incubated at 25^{0}C for 5 min, later, the reaction was terminated by dissolving 1 ml of 3 ml NH_2SO_4.

The PPO activity was determined in the form of absorbance of resultant purpurogallin at 495 nm and expressed as Umg^{-1} (U= change in 0.1 absorbance min^{-1}, mg^{-1} protein. The CAT activity was determined with an interval of 20 seconds for 2 minutes at 240 nm (Ug^{-1} FW) according to the devised method of Singh and Sharma[38]. The POX activity was observed with an interval 0f 30 sec for 2 minutes at 470 nm due to guaiacol oxidation. The activity was determined from reaction solution consisted 1 ml of 1% guaiacol, 0.3 ml of enzyme extract, 2.5 ml of 50 mM potassium buffer (pH= 7.0) and 1 ml of 1% H_2O_2 for 2 min in reaction mix [39]. The SOD activity was assessed by the estimation of volume of enzyme effected as 50% inhibition of nitro blue tetrazolium [39].

2.8. Determination of Lipid peroxidation

Malondialdehyde content (mmol g^{-1} FW) from canola leaves was determined 60 DAS [40] to estimate rate of lipid peroxidation. About 0.5 g of leaves was homogenized in 5% trichloroacetic acid (5 ml), heated at 25 ^{0}C for 10 min and centrifuged at 1800 g. The 2-thiobarbituric acid (TBA) was added in supernatant, placed at 98^{0}C for 10 min and cooled at room temperature. Finally, recorded the absorbance at 532 nm.

2.9. Statistical analysis

All the experimental data was recorded and subjected to analysis of variance (ANOVA) with linear models of statistics to observe statistical significant/non-significant differences among different traits of *Brassica napus* through computer program, Student Edition of Statistix (SWX), Version 8.1 (Analytical Software, 2005). Moreover, least significant difference (LSD) test was applied to verify the level of significance (5%) among different combination means[41].

3. Results

The results of canola presented in this manuscript was recorded for two consecutive years i.e. 2018-2019 and 2019-2020. The mean of all attributes have been tabulated and described in the result section.

3.1. Agronomic and yield performance of canola with fertilizers and PGRs under drought

Weather data from October, 2018 to April, 2020 was recorded very carefully (Table 1) and soil profile was also checked by using dry sample of soil from the experimental area (Table 2).

The mean data of two consecutive years of agronomic as well as yield attributes (plant height, days to flower, number of branches per plant, number of seed per plant, biological yield per plot, 1000 seed weight and seed yield) presented a significant ($p < 0.01$) interaction of irrigation to that of NPK and PGR (Table 3). The plant growth was affected by the severe water stress (I_4), when no elicitor was provided to the canola seedlings. The combination of chemical fertilizers (T_1) and hormone (T_2) enhanced the agronomic performance under severe water deficit (I_4=150mm evaporation) condition by improving number of seeds/plant (1.76), biological yield/plant (5.32Kg), 1000 seed weight (4.32g) and seed yield/hectare (2318Kg/ha) (Table 3**).** The low water supply during critical growth stage reduced the yield of canola (Table 3). Moreover, PGR showed non-significant difference among all levels of evaporation for plant height but significantly vary for other agronomic and yield traits. The fertilizer applications significant enhanced the biological seed yield/plant (5.43Kg) of canola and the highest yield (2450Kg/ha) was recorded with NPK (T_1) under normal irrigation (I_0). The T_1 and T_2 treatment presented a non- significant difference under normal irrigation condition. However, the T_3 treatment significantly increased the grain yield of canola under mild (I_3) and severe water stress (I_4), compared to other treatments. The maximum grain yield was recorded with $T_{1,}$ compared to T_1 under normal irrigation and declined with the severity of water stress. Moreover, data displayed a strong and significant interaction between different rate of evaporation (I) and applied elicitors (T) in all studied features of canola except leaf area (Table 3).

3.2. Physiological performance of canola with fertilizers and PGRs under drought

The mean of two-year canola data related to *Chl a*, *Chl b* and *total chlorophyll* contents presented a significant ($p < 0.01$) effect of chemical fertilizer (T_1) under water stress (Table 4). The chlorophyll contents decline in canola with increasing rate of evaporation (I_0-I_4). NPK treatment (T_1) significantly enhanced *Chl a* and total chlorophyll contents under all level of irrigation except I_4. The *Chl b* contents decreased significantly with progression of water stress and applied treatments of NPK and PGRs also fail to mitigate adverse effects of over evaporation. The T_1 and T_2 treatment of chemicals to mitigate water stress showed non-significant difference for all studied features of photosynthesis. A highly significant relation was observed between different irrigation regimes (I) and treatment with elicitors (T) for chlorophyll contents (Table 4).

The average of two season's data for water contents and temperature of canola leaves showed a significant ($p < 0.01$) interaction of irrigation and fertilizer (Table 4). Both features behave antagonistic to each other, with the decrease in leaf water content, LT increased during severe drought (I_4). The irrigation regimes I_1 and I_2 showed non-significant difference on water contents and leaf temperature and same effects were observed with chemical fertilizer (T_1) under mild (I_1) and moderate (I_2) rate of evaporation. However, the T_3 treatment increased the LWC by reducing LT of canola under mild (I_0) and severe stressful (I_4) condition (Table 4).

The NPK application significantly ($p < 0.01$) influenced the stomatal conductance of canola plant under different rate of evaporation (Table 4). The water stress reduced the stomatal conductance and showed no significant change under the mild (I_1) and moderate (I_2) irrigation stress. The combined effect of PGRs (T_2) and chemical fertilizers (T_2) significantly enhanced the stomatal conductance under normal (I_0) and mild (I_1) water stress. A drastic reduction of stomatal conductance was recorded with increasing water stress. A significant interaction of stomatal conductance (143.4**) was recorded for evaporation and applied treatments (Table 4)

3.3. Qualitative performance of canola with fertilizers and PGRs under drought

The mean of two years statistical data depicted that the interactive effect of irrigation and fertilizers significantly influenced the quality related traits (oil contents, oil yield, protein, glucosinolates, and moisture and erucic acid contents) of canola (Table 5). These features decline with the progression of water deficit condition. The maximum oil contents, oil yield protein and moisture contents were recorded with T_3 under normal (I_0) and severe water deficit (I_4) condition, while the glucosinolates and erucic acid declined the same treatment (Table 5). It is summarized that PGRs (T_2) is a good rehabilitation technique to improve quality of canola followed by chemical fertilizers (T_1) to address water shortage issues of canola.

3.4. Nitrogen, Phosphorus and Potassium (NPK) contents and their Uptake in canola

The mean data of two consecutive years showed that combined effect of fertilizer and PGRs (T_2) significantly ($p < 0.01$) influence the NPK contents and their uptake in canola plant. Provision of chemical fertilizer (T_1) individually or in combination with PGRs (T_3) improved the NPK contents as well as their uptake under mild (I_0) irrigation stress (Table 6).

3.5. Biochemical performance of canola with fertilizers and PGRs under drought

The results obtained from two-year trails illustrated that the osmolytes considerably ($p < 0.01$) influenced by the applied elicitors under different irrigation regimes. The level of proline and soluble sugar elevated in response to decreasing water supply to canola plant as an innate response mechanism. Further, the NPK and PGRs application showed a negative impact on the proline accumulation but positively enhanced soluble sugar contents under all level of irrigation (Table 7). The maximum osmolyte accumulation was recorded with the application of NPK (T_1) followed by PGRs (T_2) and combined treatment (T_3) under all levels of irrigation (Table 7).

The antioxidant enzymes and MDA activities significantly ($p < 0.01$) influenced by the irrigation (I) and elicitor treatments (T) in canola (Table 7). The canola plant enhanced the activities of various antioxidants and enzymes including SOD, CAT, PPO, POX and MDA contents in response to water stress. The canola behavior with fertilizers did not show significant effect during normal (I_1) and mild irrigation (I_2) stress except lipid peroxidation and POX activity. The T_1 treatment exhibited non-significant difference on enzyme activities but the MDA content influenced significantly under all irrigation levels (I_0-I_4). The treatment T_2 (PGRs) and T_3 (NPK and PGRs) elevated the activities SOD, PPO, POX and SOD but reduced the MDA contents as compared to T_0 under severe water deficit (I_4) condition. (Table 7).

The interaction of irrigation intervals (I) and elicitors (T) significantly affect the membrane stability index (MSI) of canola ($p < 0.01$). The water stress significantly reduced the membrane stability index. Only chemical fertilizer did not effect on the MSI while the combined fertilizers and plant growth regulators significantly improve the MSI under moderate and severe water stress. The chemical fertilizer only showed superiority under irrigation level of I_3 (Table 7).

4. Discussion

The findings of the current work highlights the comparison of combined and individual application of PGR and NPK to enhance canola growth, nutritional quality and yield under water scarcity. Drought imparts drastic effect on the plant growth by causing injury at all growth stages. It influences the various morphological (reduced leaf growth, leaf area, plant height, number of nodes per plant), physiological traits (chlorophyll content, leaf water contents, leaf temperature and stomatal conductance) at the onset of water scarcity (Table 3, 4). Reduction of plant height was recorded in canola under different irrigation regimes compared to exogenously applied PGRs and NPK (Table 3). Growth retardation due to excessive evaporation, can be related to disruption

of photosynthetic machinery and decline in carbon reserves for relocation to growing parts of plant [42,43]. It seems that the decrease in plant height also interferes with leaf area. This reduction is particularly noticeable during post vegetative stage, flowering stage or abscission [43]. Growth regulators and chemical fertilizers were significantly effective in mitigating the drastic effects of drought by maintaining the water efficiency of canola plants and augmenting the accretion of osmolytes. This may also favors the improvement of photosynthetic and gas exchange attributes [15].

In the current experiment, drought led to reduction in chlorophyll content, and this loss could be due to some devastating effects on photosynthetic apparatus (Table 3). Stomatal conductance (g_s) was severely hampered when plants were exposed to a water deficit condition. This may have been resulted for enhanced production of ABA under drought stress that leads to stomata closure. This suggested that the ABA signaling mechanism tries to prevent the loss of tissue turgor by closing the stomata [44,45]. The optimal use of chemical fertilizers and PGR appreciably enhanced stomatal conductance under drought stress (Table 4). Yan et al. [46] Reported that diffusional restrictions of CO_2 by stomata (52%) could explain loss of Chlorophyll contents (31%) induced by drought. The fertilizer application especially urea increase the N supply at flowering and pod filling stage, delay leaf aging, enhanced chlorophyll contents and photo assimilates [47]. The total amount of chlorophyll contents increased by the availability of nitro compounds in the rhizosphere and consequently to the plants, ultimately produced more assimilates via photosynthesis which directly related to improve growth and yield [9,48].

The NPK treatment increases the flow of nutrient to the aerial part of plants and reduced the impact of water stress (Table 4,6). Due to water scarcity and loss of ionic balance, nutrients remained bond to the soil particles that are critical for the normal growth and development [49,50]. Same findings have been reported in the present work in the form of decreased biological yield (40%) with non-availability of nutrients (5-10%).

Canola oil is the commercial commodity, while its content, profile and composition are affected by drought stress as reported in the current work (Table 5). Seed oil stems mostly from photosynthesis and green silique walls, later carbon is routed through different metabolic pathways into triacylglycerol occurring in the chloroplast, cytosol, and endoplasmic reticula [51]. The

current experiment suggests 3–11% decrease in oil content and oil yield in the *B. napus* when the plants were exposed to irrigation stress, but [52] reported a mere 3.2% reduction.

The grain yield reduced due to water stress as reported in pervious study [53]. The grain yield and quality improved in bio fertilizer treated plants under water stress because it increases the activities of CAT, POX, PPO and SOD, assimilation rate of CO_2, MSI, RWC, Stomatal conductance, Ci, N, K and Ca concentration in leaf tissue (Table 5,7) [9]. The percentage of NPK uptake enhanced with the combined application of NPK and PGRs (Table 5). The bio fertilizers improve the soil textures and bacterial colonization with the modification of physio-chemical properties of rhizosphere. On the other hand, the PGRs increased the photo assimilates translocation to sink (root tissue) and also improved the nutrient uptake and absorption power under water deficit and adverse environment condition. The PGRs can enhanced the activity of some vital N, P and K metabolizing enzymes in plant which enhanced NPK contents under different irrigation regimes [48,54].

All plants have been equipped with innate antioxidant enzyme mechanisms for the detoxification of reactive oxygen species. CAT decomposes H_2O_2 into water and molecular O_2. POX dismutases H_2O_2 by oxidizing co-substrates such as phenolic compounds and/or antioxidants and PPO in urn oxidizes phenols to chinone [55].

Membrane lipid peroxidation is a frequently used indictor to test the degree of plant sensitivity to oxidative damage caused by stress [56]. Our data revealed sensitivity of canola towards drought by elevation MDA content compared to F_2, F_3 treatments (Table 7). A lower level of lipid peroxidation presented high membrane stability (Table 7). It seems that the cell membrane integrity was maintained with chemical fertilizers and PGRs against the oxidative stress induced by water stress (Table 7). Mamnabi et al.[9] also suggested an amplification of antioxidant enzymes, total soluble sugars, photosynthetic attributes, leaf water content, membrane stability index and stomatal conductance but decreased the leaf temperature under different irrigation regimes.

The affirmative role of fertilizers and PGRs increase the antioxidant enzyme activities under water deficient condition in canola [57]. The PGRs treated plant showed more activities of antioxidant enzymes like POD, CAT and PPO as compared to untreated plants under moderate and severe water stress. The highest antioxidant activities were observed in T_3 treatments under

severe stress as compared to T_1 and T_2 (Table 7). This supremacy was attained by the additive effect of fertilizer and PGRS on canola plants.

Conclusion

Canola is an emerging and unique oil seed crop among the other oil producing plants due to low erucic acid and glucosinolate contents. Drought is negatively affecting growth, chlorophyll contents and availability of nutrients to the canola which directly reducing seed yield and seed oil. The oxidative injuries caused by water stress resulted in diminution of chlorophyll content, MSI, and stomatal conductance of rapeseed plants. Application of growth promoting substances in canola enhanced growth, physiological response under different irrigation regimes. The harmful effects of stress were minimized considerably by the combined application of fertilizers and PGRs, thus improved growth attributes, chlorophyll content, MSI, stomatal conductance, antioxidants, osmoprotectants, grain yield and importantly, leading to a reduction in lipid peroxidation, particularly under moderate and severe rate of evaporation. These supremacies were attained by additive effects of NPK and PGPR, reducing the impact of drought.

Acknowledgements

Mohammad Aquil Siddiqui & Muhammad Mahran Aslam conceive the ideal, conducted the experiment, statistical analysis and wrote the first draft of paper. Fozia Farhat conducted the analysis of physiological and biochemical parameters at Government College Women University Faisalabad .wrote first draft of the manuscript and finalized it. Shafquat Yasmeen and Muhammad Tahir Khan helped in collecting the agronomic data of the crop and formatting the paper. Mahboob Ali Sial & Imtiaz Ahmad Khan review the draft and provided technical inputs.

References

1. Sha W, Enli W, Fei W, Liang T (2012) Phenological development and grain yield of canola as affected by sowing date and climate variation in the Yangtze River Basin of China. Crop and Pasture Science 63: 478-488.
2. Raza A, Razzaq A, Mehmood SS, Zou X, Zhang X, et al. (2019) Impact of Climate Change on Crops Adaptation and Strategies to Tackle Its Outcome: A Review. Plants (Basel, Switzerland) 8: 34.
3. Mitrović PM, Stamenković OS, Banković-Ilić I, Djalović IG, Nježić ZB, et al. (2020) White Mustard (Sinapis alba L.) Oil in Biodiesel Production: A Review. Frontiers in plant science 11: 299-299.
4. Selim MM (2020) Introduction to the Integrated Nutrient Management Strategies and Their Contribution to Yield and Soil Properties. International Journal of Agronomy 2020: 2821678.
5. Fahad S, Bajwa AA, Nazir U, Anjum SA, Farooq A, et al. (2017) Crop Production under Drought and Heat Stress: Plant Responses and Management Options. Frontiers in Plant Science 8.
6. Hussain HA, Hussain S, Khaliq A, Ashraf U, Anjum SA, et al. (2018) Chilling and Drought Stresses in Crop Plants: Implications, Cross Talk, and Potential Management Opportunities. Frontiers in plant science 9: 393-393.
7. Gupta B, Huang B (2014) Mechanism of Salinity Tolerance in Plants: Physiological, Biochemical, and Molecular Characterization. International Journal of Genomics 2014: 701596.
8. Urban L, Aarrouf J, Bidel LPR (2017) Assessing the Effects of Water Deficit on Photosynthesis Using Parameters Derived from Measurements of Leaf Gas Exchange and of Chlorophyll a Fluorescence. Frontiers in plant science 8: 2068-2068.
9. Mamnabi S, Nasrollahzadeh S, Ghassemi-Golezani K, Raei Y (2020) Improving yield-related physiological characteristics of spring rapeseed by integrated fertilizer management under water deficit conditions. Saudi Journal of Biological Sciences 27: 797-804.
10. Yin Z, Lu J, Meng S, Liu Y, Mostafa I, et al. (2019) Exogenous melatonin improves salt tolerance in tomato by regulating photosynthetic electron flux and the ascorbate–glutathione cycle. Journal of Plant Interactions 14: 453-463.
11. Das K, Roychoudhury A (2014) Reactive oxygen species (ROS) and response of antioxidants as ROS-scavengers during environmental stress in plants. Frontiers in Environmental Science 2.
12. Rezayian M, Ebrahimzadeh H, Niknam V (2020) Nitric Oxide Stimulates Antioxidant System and Osmotic Adjustment in Soybean Under Drought Stress. Journal of Soil Science and Plant Nutrition 20: 1122-1132.
13. Khaleghi A, Naderi R, Brunetti C, Maserti BE, Salami SA, et al. (2019) Morphological, physiochemical and antioxidant responses of Maclura pomifera to drought stress. Scientific Reports 9: 19250.
14. Laxa M, Liebthal M, Telman W, Chibani K, Dietz K-J (2019) The Role of the Plant Antioxidant System in Drought Tolerance. Antioxidants (Basel, Switzerland) 8: 94.
15. Sharma A, Shahzad B, Kumar V, Kohli SK, Sidhu GPS, et al. (2019) Phytohormones Regulate Accumulation of Osmolytes Under Abiotic Stress. Biomolecules 9: 285.

16. Hasanuzzaman M, Bhuyan MHMB, Zulfiqar F, Raza A, Mohsin SM, et al. (2020) Reactive Oxygen Species and Antioxidant Defense in Plants under Abiotic Stress: Revisiting the Crucial Role of a Universal Defense Regulator. Antioxidants 9: 681.
17. Bindraban PS, Dimkpa C, Nagarajan L, Roy A, Rabbinge R (2015) Revisiting fertilisers and fertilisation strategies for improved nutrient uptake by plants. Biology and Fertility of Soils 51: 897-911.
18. Khattab EA, Afifi MH, Amin GA (2019) Significance of nitrogen, phosphorus, and boron foliar spray on jojoba plants. Bulletin of the National Research Centre 43: 66.
19. Anjum NA, Gill SS, Umar S, Ahmad I, Duarte AC, et al. (2012) Improving Growth and Productivity of Oleiferous Brassicas under Changing Environment: Significance of Nitrogen and Sulphur Nutrition, and Underlying Mechanisms. The Scientific World Journal 2012: 657808.
20. Yagoub SO, Ahmed WMA, Mariod AA (2012) Effect of Urea, NPK and Compost on Growth and Yield of Soybean (<i>Glycine max L.</i>), in Semi-Arid Region of Sudan. ISRN Agronomy 2012: 678124.
21. Shirazi SM, Yusop Z, Zardari NH, Ismail Z (2014) Effect of Irrigation Regimes and Nitrogen Levels on the Growth and Yield of Wheat. Advances in Agriculture 2014: 250874.
22. Manik SMN, Pengilley G, Dean G, Field B, Shabala S, et al. (2019) Soil and Crop Management Practices to Minimize the Impact of Waterlogging on Crop Productivity. Frontiers in plant science 10: 140-140.
23. Rezaei R, Valadabadi SAR, Shirani Rad AH, Sayfzadeh S, Hadidi Masouleh E (2018) Effects of biological fertilizers application and various amounts of urea fertilizers under water stress conditions on yield, nitrogen use efficiency and effective ingredients of Calendula officinalis L. Iranian Journal of Medicinal and Aromatic Plants Research 34: 547-564.
24. Bhardwaj D, Ansari MW, Sahoo RK, Tuteja N (2014) Biofertilizers function as key player in sustainable agriculture by improving soil fertility, plant tolerance and crop productivity. Microbial cell factories 13: 66-66.
25. Mondal T, Datta J, Mondal N (2017) Chemical fertilizer in conjunction with biofertilizer and vermicompost induced changes in morpho-physiological and bio-chemical traits of mustard crop. Journal of the Saudi Society of Agricultural Sciences 16: 135-144.
26. Sevinç K (2018) Effects of Vermicompost on Some Morphological, Physiological and Biochemical Parameters of Lettuce (Lactuca sativa var. crispa) under Drought Stress. Notulae Botanicae Horti Agrobotanici Cluj-Napoca 47.
27. Mukhongo RW, Tumuhairwe JB, Ebanyat P, AbdelGadir AH, Thuita M, et al. (2017) Combined Application of Biofertilizers and Inorganic Nutrients Improves Sweet Potato Yields. Frontiers in Plant Science 8.
28. Stalikas CD (2007) Extraction, separation, and detection methods for phenolic acids and flavonoids. J Sep Sci 30: 3268-3295.
29. Rathje (1959) Jackson, M. L.: Soil chemical analysis. Verlag: Prentice Hall, Inc., Englewood Cliffs, NJ. 1958, 498 S. DM 39.40. Zeitschrift für Pflanzenernährung, Düngung, Bodenkunde 85: 251-252.
30. Tandon HLS, Cescas, M.P., Tyner, E.H., (1968) An acid-free vanadate-molybdate reagent for the determination of total phosphorus in Soils Soil Sci Soc Am J 1: 48-51.
31. Pimstein A, Karnieli A, Bansal SK, Bonfil DJ (2011) Exploring remotely sensed technologies for monitoring wheat potassium and phosphorus using field spectroscopy. Field Crops Research 121: 125-135.

32. Tammam AA (2008) Study of salt tolerance in wheat (Triticum aestium L.) cultivar Banysoif 1. Australian journal of crop science v. 1: pp. 115-125-2008 v.2001 no.2003.
33. Arnon DI (1949) COPPER ENZYMES IN ISOLATED CHLOROPLASTS. POLYPHENOLOXIDASE IN BETA VULGARIS. Plant physiology 24: 1-15.
34. Ghassemi-Golezani K, Ghassemi, S., Yaghoubian, I. (2016) Salicylic Acid regulate Physiological Performance of milk thistle (Silybum marianum L.) under water stress. Adv Biores: 34-40.
35. Kochert G (1978) Carbohydrate determination by the phenol-sulfuric acid method. Handbook. Phycol Meth: 95-97.
36. Bates LS, Waldren RP, Teare ID (1973) Rapid determination of free proline for water-stress studies. Plant and Soil 39: 205-207.
37. Kumar KB, Khan, P.A. (1982) Peroxidase and polyphenoloxidase in excisedragi (Eleusine coracana cv. PR 202) leaves during senescence. . The Indian Journal of Experimental Biology: 412–416.
38. Singh BK, Sharma SR, Singh B (2010) Antioxidant enzymes in cabbage: Variability and inheritance of superoxide dismutase, peroxidase and catalase. Scientia Horticulturae 124: 9-13.
39. Gueta-Dahan Y, Yaniv Z, Zilinskas BA, Ben-Hayyim G (1997) Salt and oxidative stress: similar and specific responses and their relation to salt tolerance in citrus. Planta 203: 460-469.
40. Janero DR (1990) Malondialdehyde and thiobarbituric acid-reactivity as diagnostic indices of lipid peroxidation and peroxidative tissue injury. Free Radic Biol Med 9: 515-540.
41. Gomez KAaG, A. (1984) Statistical Procedure for Agricultural Research—Hand Book. John Wiley & Sons New york.
42. Shahid MA, Sarkhosh A, Khan N, Balal RM, Ali S, et al. (2020) Insights into the Physiological and Biochemical Impacts of Salt Stress on Plant Growth and Development. Agronomy 10: 938.
43. Sharif P, Seyedsalehi M, Paladino O, Van Damme P, Sillanpää M, et al. (2018) Effect of drought and salinity stresses on morphological and physiological characteristics of canola. International Journal of Environmental Science and Technology 15: 1859-1866.
44. Wilkinson S, Davies WJ (2010) Drought, ozone, ABA and ethylene: new insights from cell to plant to community. Plant Cell Environ 33: 510-525.
45. Pantin F, Monnet F, Jannaud D, Costa JM, Renaud J, et al. (2013) The dual effect of abscisic acid on stomata. New Phytologist 197: 65-72.
46. Yan W, Zhong Y, Shangguan Z (2016) A meta-analysis of leaf gas exchange and water status responses to drought. Scientific Reports 6: 20917.
47. Gai Z, Zhang J, Li C (2017) Effects of starter nitrogen fertilizer on soybean root activity, leaf photosynthesis and grain yield. PLOS ONE 12: e0174841.
48. Razaq M, Zhang P, Shen H-l, Salahuddin (2017) Influence of nitrogen and phosphorous on the growth and root morphology of Acer mono. PLOS ONE 12: e0171321.
49. Elferjani R, Soolanayakanahally R (2018) Canola Responses to Drought, Heat, and Combined Stress: Shared and Specific Effects on Carbon Assimilation, Seed Yield, and Oil Composition. Frontiers in Plant Science 9.
50. Barnabás B, Jäger K, Fehér A (2008) The effect of drought and heat stress on reproductive processes in cereals. Plant Cell Environ 31: 11-38.

51. Baud S, Lepiniec L (2010) Physiological and developmental regulation of seed oil production. Prog Lipid Res 49: 235-249.
52. Aslam MN, Nelson MN, Kailis SG, Bayliss KL, Speijers J, et al. (2009) Canola oil increases in polyunsaturated fatty acids and decreases in oleic acid in drought-stressed Mediterranean-type environments. Plant Breeding 128: 348-355.
53. Flexas J, Medrano H (2002) Drought-inhibition of photosynthesis in C3 plants: stomatal and non-stomatal limitations revisited. Ann Bot 89: 183-189.
54. Waqas MA, Khan I, Akhter MJ, Noor MA, Ashraf U (2017) Exogenous application of plant growth regulators (PGRs) induces chilling tolerance in short-duration hybrid maize. Environmental Science and Pollution Research 24: 11459-11471.
55. Apel K, Hirt H (2004) Reactive oxygen species: metabolism, oxidative stress, and signal transduction. Annu Rev Plant Biol 55: 373-399.
56. Blokhina O, Virolainen E, Fagerstedt KV (2003) Antioxidants, oxidative damage and oxygen deprivation stress: a review. Ann Bot 91 Spec No: 179-194.
57. Khan N, Bano A, Babar MDA (2020) Impacts of plant growth promoters and plant growth regulators on rainfed agriculture. PloS one 15: e0231426-e0231426.

Manuscript Click here to access/download;Manuscript;Manuscript.docx

Physiological and Biochemical Exploration of Canola for Overcoming Yield Limitation by Integrated Fertilizer management under different water regimes

Muhammad Mahran Aslam[a], Fozia Farhat[b], Mohammad Aquil Siddiqui[a*], Shafquat Yasmeen[a], Muhammad Tahir Khan[a], Mahboob Ali Sial[a] and Imtiaz Ahmad Khan[a]

[a]Nuclear Institute of Agriculture (NIA), Tando Jam, 70060, Sindh, Pakistan

[b]Department of Botany, Government College Women University, Faisalabad, Pakistan

[*]Corresponding author: Mohammad Aquil Siddiqui[a]

Corresponding author email: Siddiqui_aquil@yahoo.com

ABSTRACT

Environmental stressors may modify the nutritional profile and economic value of crops. Chemical fertilizers and phyto-hormones are major natural sources which can enhance the canola production under stressful conditions. Physio-biochemical responses of canola altered remarkably with the use of nitrogen/phosphorus/potassium (N/P/K) fertilizers and plant growth regulators (PGRs) under drought stress. The key interest of current study was to evaluate nutritional quality and physio-biochemical modulation in canola (*Brassica napus* L.) from early growth to seed stage with chemical fertilizers (NPK) and plant growth regulators (PGRs) in different water regimes. To monitor responses in canola (Surhan-2012), two separate field experiments were conducted as spilt plot under randomized complete block design (RCBD) with four treatments (No fertilizer, Chemical fertilizers, PGRs and the combination of chemical fertilizers and PGRs) under different irrigations regimes (60, 100, 120, 150 mm evaporations). Water stress enhanced activity of peroxidase (POD), catalase (CAT), superoxide dismutase (SOD), polyphenol oxidase (PPO), soluble sugar, leaf temperature, malondialdehyde (MDA) and proline contents while substantially reduced leaf water contents, stomatal conductance, chlorophyll contents, membrane stability index and grain yield of canola. However, the combined application of fertilizers and plant growth regulator (PGR) further increased the enzymatic antioxidant pool, soluble sugars, along with recovery of leaf water contents, chlorophyll contents, stomatal conductance and membrane stability index but decreased the proline contents and leaf temperature at different rate of evaporation. There is positive interaction of applied elicitors to the water stress in canola except leaf area. The outcomes depicted that the combination of NPK with PGRs improved the various morpho-physiological as well as biochemical parameters and reduced the pressure of chemical fertilizers cost about 60%. It had also reduced the deleterious effect of water limitation on the physiology and grain yield and oil contents of canola in field experiments.

Keywords: antioxidants, elicitors, mitigation strategies, photosynthesis, water shortage, yield

1. Introduction

Among oilseed crops, canola (*Brassica napus* L.) withhold a special rank worldwide as the second highly significant oil yielding crop [1] with over ~40–50% oil and 40% protein (in rapeseed meal) contents after soybean [2]. With constant efforts of scientists, the production of canola had reached a milestone by enhancing its yield and area upto 68.9 million metric tons and 33.7 million

hectares, respectively [3]. The leaves of this plant are imperative source of animal feed due to encompassing a balanced ratio of protein and fibrous [4]. Oil extracted from oilseed rape contains a very high concentration of unsaturated fatty acids (such as oleic acid and linoleic acid (C18:3, ~10% v/v)) making it a fatty acid-rich diet source [5]. Pakistan facing the low yield of canola due to the inefficient management of macro and micronutrient. Globally, it is emerging oil seed crop among the other oil seeds due to low erucic acid and glucosinolates in oil and seed cake respectively [6].

Like many other major temperate field crops, canola is particularly susceptible to environmental stress alone and in combinations of multiple stresses, such as heat and drought [7, 8, 9]. Reduced biomass and chlorophyll contents due to deterioration of chloroplast structure along with reduced production of seed oil and protein contents are usual symptoms of water stress in *B. napus* [10]. The plant growth and development mainly restricted by the drought stress via declining turgor pressure of plant cells which caused hindrance in the biochemical and physiological intriguing mechanisms [11]. The water deficit condition cause the reduced concentration of intracellular CO_2, chlorophyll destruction, photochemical system disorder and stomatal closure [12]. The plant showed physiological response in the form of reactive oxygen species (ROS) production under drought stress. The excess amount of ROS can damage the cell membrane by elevating the lipid peroxidation [13,14]. The plants mitigate the ROS harmful effect by the activities of enzymatic and non- enzymatic antioxidants [15]. Superoxide dismutase (SOD), peroxidase (POX), catalase (CAT) and polyphenol peroxidase (PPO) are the antioxidant enzymes which diminished the ROS concentration during drought stress [13,16]. The accumulation of osmolytes, proline, soluble sugar, soluble proteins are the non-enzymatic reaction that caused osmatic regulation under water deficient condition stress [17]. The antioxidant activities elevated under stress condition in ajowan and canola plant against ROS [18]. The plants showed increase level of proline, antioxidants including POX. PPO and SOD but reduced MDA level under stress condition [19, 20].

Nitrogen play a pivotal role in plant tissue growth and development, being an integral part of protein, chlorophyll, nucleotides, protein, amino acid which directly influence the quality and quantity of crop production [21]. The adequate nitrogen supply is important attribute to boost up the canola productivity [22]. Thus, any fluctuation in the soil profile, texture, and moisture content

at various critical stage of growth and development may sway the nitrogen use efficiency in canola. Canola crop is very responsive to fertilizer application especially nitrogen, phosphorus and potassium which significantly effects the growth and yield per ha [23]. It also stimulate the leaf area (LA) development after flowering in canola [24,25].

The management of soil fertility is limiting factor for the sustainable agriculture production [23]. This challenges of soil fertility can be overcome by the optimal application of fertilizers and plant growth regulators [26]. The chemical and bio-fertilizer are very effective in improving the micro and macro nutrient via organic compound degradation and nitrogen fixation [27]. This lead to improve nutrient uptake and reduce impact of drought in crops. This would increase the activities of PPO, CAT and POX which ultimately improve the grain yield under drought stress in response to fertilizer and PGRs [13]. The combined application of chemical fertilizers, bio fertilizers and PGRs enhanced the accumulation of proline, sugar contents and chlorophyll contents [28]. The fertilizers along with plant growth regulators elevated the stomatal conductance, water contents and total chlorophyll content under water limiting conditions [29]. These combined application of nutrients improve the soil fertility under water stress condition [30]. Although genetic manipulation has a promising effects to mitigate this problem and also stated mid- and long-term results, but current world situations required some immediate response methods for the growing demands of food and feed. In spite of numerous scientific efforts, information related to biochemical, physiological and yield dynamics with respect to combined application of chemical fertilizers and PGRs is very limited for canola production. Thus, this manuscript comprised to gauge the morphological, physiological and biochemical rejoinders of canola to assimilate fertilizers management and PGRs with limited supply of water.

2. Materials and Methods

2.1. Experimental design and treatments

The two-year (2018-19 & 2019-2020) field experiment was conducted at experimental field of Nuclear Institute of Agriculture (NIA), TandoJam, Sindh to investigate the variability in physio-biochemical parameters and yield of canola (Surhan-2012) in reaction to fertilization under excessive rate of water evaporation. The experiment was performed in split plots as random complete block design (RCBD) in triplicate with four irrigation levels.

**Irrigation Levels**	**Treatment with Elicitors**
1_0= 60mm evaporation	T_0 = No treatment
	T_1= Chemical Fertilizers [N (90 kg/ha), P (45 kg/ha) and K (45 kg/ha)]
	T_2 = PGRs [IAA (15g/ha) and GA_3 (15g/ha)]
	$T_3 = T_1 + T_2$
1_1= 100mm evaporation	T_0 = No treatment
	T_1 = Chemical Fertilizers [N (90 kg/ha), P (45 kg/ha) and K (45 kg/ha)]
	T_2 = PGRs [IAA (15g/ha) and GA_3 (15g/ha)]
	$T_3 = T_1 + T_2$
1_2= 120mm evaporation	T_0 = No treatment
	T_1 = Chemical Fertilizers [N (90 kg/ha), P (45 kg/ha) and K (45 kg/ha)]
	T_2 = PGRs [IAA (15g/ha) and GA_3 (15g/ha)]
	$T_3 = T_1 + T_2$
1_3= 150mm evaporation	T_0 = No treatment
	T_1 = Chemical Fertilizers [N (90 kg/ha), P (45 kg/ha) and K (45 kg/ha)]
	T_2 = PGRs [IAA (15g/ha) and GA_3 (15g/ha)]
	$T_3 = T_1 + T_2$

Weeding operations was done frequently during growth and development of surhan-2012 plants in both years.

2.2. Determination of Agronomic and yield related parameters

At vegetative stage, height of ten randomly selected canola plants of each treatment were measured with measuring tape (cm) from ground to the tip of flag leaf and counted number of branches per plant while number of siliqua plant^{-1}, siliqua length (cm) and number of seeds siliqua^{-1} were counted after harvesting. Similarly, recorded the data related to days to flower when 50% flowers had been appeared on plants and days to maturity at discoloration stage. Grains of canola plants were dried in the sun and recorded 1000 grain weight by using digital balance (Model-Explorer OHAUS). Biological yield was determined after harvesting from 4 rows and calculated as kg ha^{-1}.

2.3. Determination of Quality traits

Oil content were extracted with petroleum ether using soxhlet apparatus. All the dried seed samples were coarsely ground and packed carefully into the thimble for oil extraction. The extraction was performed continuously for three cycles (90-120 min.) and oil productivity was

drawn through standard formula. Two significant fatty acids, erucic acid (%) and glusionlate (µmol g^{-1}) were detected through High Performance Liquid Chromatography (HLPC). Oil contents were analysed using Gradient HPLC (Shimadzu, Japan) having LC-10AT, SCTL 10A system controller, SPD-10AR UV-VIS detector at 280 nm with C18 stationary column (Shim-Pack CLC-ODS). Elution was done for 60 min with a flow rate of 1ml/min in a gradient system of two mobile phases A (H_2O_2: AA-94:6, pH 2.27), B (ACN100%)[31]. Moisture contents (%) was determined by the weight of water in a seed.

2.4. Determination of NPK uptake by the Canola Grain

For determination of phosphorus (P) and potassium (K) contents, seed samples of each treatment were dried at 70°C for 48h. Dried and powdered grain sample (0.5g) was digested with 20mL concentrated nitric acid (HNO_3) by adopting method of Rathje and Jackson [32]. The samples were placed for 3 hours at room temperature. After 3 hours, samples were laid on the digestion block at 250° C until the solution became tinted yellow in appearance. The digested solution was diluted with 50mL of distilled water and filtered with whatman No. 42 filter paper. The phosphorus (P) contents from the digested plant samples was determined by recording optical density at 430 nm with spectrophotometer (Model-Spectronic-21) by Primson et al. [33]. The potassium (K) content in gains was resolute by flame photometer (Model-Flame photometer-400) according to the method suggested by Tammam [34]. Nitrogen content was examined by Kjeldahl apparatus [35]. Following formulas were applied to determined NPK uptake in grains of canola as kg ha^{-1}.

N uptake (kg ha^{-1}) in grain: N (%) x grain yield (kg ha^{-1}) /100

P uptake (kg ha^{-1}) in grain: P (%) x grain yield (kg ha^{-1}) /100

K uptake (kg ha^{-1}) in grain: K (%) x grain yield (kg ha^{-1}) /100

2.5. Determination of Physiological and Biochemical parameters

2.5.1. Chlorophyll Contents

Fresh leaf samples were collected from each treatment and subjected to grinding with 80% acetone. Semi-liquid extract was filtered and centrifuged at 10000rpm for 5minutes [36]. The supernatant was then subjected to spectrophotometer (Model Analytikjena Spekol 1500 Germany)

2.5.2. Leaf water content

Leaf water content was measured by harvesting three leaves per plant from every plot after 45 days of sowing (DAS). Fresh leaf sample was weighed in gram (g) as fresh weight (FW) and let them dry at high temperature (80^0C) and reweighed as dry weight (DW). Leaf water content (LWC) was calculated by following formula.

$$LWC = [(FW – DW) / (FW)]$$

2.5.3. Leaf temperature

The leaf temperature (LT) was measured at flowering stage with the help of infrared thermometer (TES- 1327). The leave temperature (^{0}C) was measured by randomly selecting 3 plants of every treatment and replicate. Later, the mean LT was carefully recorded.

2.5.4. Stomatal conductance (g_s)

Portable photosystem (Porometer AP4, Delta-T Devices Ltd., Cambridge, U.K.) was used to measure the stomatal conductance. This data was carefully recorded 60 days after sowing (DAS).

2.5.5. Membrane stability index (MSI)

The previously reported method of Ghassemi-Golezani et al. [37] with slight modification was used to calculate membrane stability index. ˜0.1 g leaf samples was mixed with double distilled water (10 ml) in falcon tube and incubated at 40^0C for 30 min and electrical conductivity was measured (EC_1). Thereafter conductivity of these sample were assessed after placing water bath at 100^0C for 10 min (EC_2). The MSI was measured by the following formulas:

$$MSI = (EC_1 / EC_2) \times 100$$

2.6. Determination of osmolytes

The total soluble sugar content was estimated from the dried leaves of all the replicates of respective canola treatments [38]. The standard calibration curve of pure glucose was used to determine total soluble sugars of leaves and expressed as mg/g DW. To determine proline contents in canola, leaf sample was thoroughly grinded in 3% sulfosalicylic acid. The extracted sample was filitered, mixed with glacial acetic acid and ninhydrin in a test tube with a ratio of 1:1:1. This mixture was heated at 100^0C for 60 min in a Bain Marie oven. Then reaction mixture was cooled at room temperature and the toluene used for the extraction of mixture, vortexed for 30sec. The

absorbance of the upper organic phase was recorded at 520 nm. Calibration curve of pure proline was used to compare the proline content of canola leaves and expressed as mg/g FW [39].

2.7. Determination of Antioxidants

Young leaves were collected from each treatment at 60 DAS and assayed the activity of polyphenol oxidase (PPO) by Kumar and Khan (1982) method [40]. The reaction mixture contains 0.1 M phosphate buffer, 1 ml catechol and 5 ml enzyme extract. The reaction mixture was incubated at 25^0C for 5 min, later, the reaction was terminated by dissolving 1 ml of 3 ml NH_2SO_4. The PPO activity was determined in the form of absorbance of resultant purpurogallin at 495 nm and expressed as Umg^{-1} (U= change in 0.1 absorbance min^{-1}, mg^{-1} protein. The CAT activity was determined with an interval of 20 seconds for 2 minutes at 240 nm (Ug^{-1} FW) according to the devised method of Singh and Sharma[41]. The POX activity was observed with an interval 0f 30 sec for 2 minutes at 470 nm due to guaiacol oxidation. The activity was determined from reaction solution consisted 1 ml of 1% guaiacol, 0.3 ml of enzyme extract, 2.5 ml of 50 mM potassium buffer (pH= 7.0) and 1 ml of 1% H_2O_2 for 2 min in reaction mix [42]. The SOD activity was assessed by the estimation of volume of enzyme effected as 50% inhibition of nitro blue tetrazolium [42].

2.8. Determination of Lipid peroxidation

Malondialdehyde content (mmol g^{-1} FW) from canola leaves was determined 60 DAS to estimate rate of lipid peroxidation [43]. ˜0.5 g of fresh leaves was homogenized in 5% trichloroacetic acid (5 ml), heated at 25^0C for 10 minutes and centrifuged at 1800g. The 2-thiobarbituric acid (TBA) was added in supernatant, placed at 98^0C for 10 min and cooled at room temperature. Finally, recorded the absorbance at 532 nm with spectrophotometer.

2.9. Statistical analysis

All the experimental data was recorded and subjected to analysis of variance (ANOVA) with linear models of statistics to observe statistical significant/non-significant differences among different traits of *Brassica napus* through computer program, Student Edition of Statistix (SWX), Version 8.1 (Analytical Software, 2005). Moreover, least significant difference (LSD) test was applied to verify the level of significance (5%) among different combination means[44].

3. Results

The results of canola presented in this manuscript was recorded for two consecutive years i.e. 2018-2019 and 2019-2020. The mean of all attributes have been tabulated and described in the result section (Table 1-7). Weather data was carefully monitored from October to April for both growing years (2018-19/2019-20). The maximum and minimum temperature decreases from October to March and then increases marginally in April for both growing seasons (Table 1). The soil of experimental area was analyzed and found 2% organic matter (OM) along with sodium (0.08), potassium (0.11), magnesium (3.2), sulpher (16.90), zinc (1.40), boron (0.34), phosphorus (10.83) and calcium (2.5) as major nutrients. The soil mineral contents were calculated as µg/g soil (Table 2).

3.1. Agronomic and yield performance of canola with fertilizers and PGRs under drought

The mean data of two consecutive years of agronomic as well as yield attributes (plant height, days to flower, number of branches per plant, number of seed per plant, biological yield per plot, 1000 seed weight and seed yield) presented a significant ($p < 0.01$) interaction of irrigation to that of NPK and PGR (Table 3). The plant growth was affected by the severe water stress (I_4), when no elicitor was provided to the canola seedlings. The combination of NPK (T_1) and PGRs (T_2) enhanced the agronomic performance under severe water deficit (I_4=150mm evaporation) condition by improving number of seeds/plant (1.76), biological yield/plant (5.32Kg), 1000 seed weight (4.32g) and seed yield/hectare (2318Kg/ha) (Table 3**).** The low water supply during critical growth stage reduced the yield of canola (Table 3). Moreover, PGR showed non-significant difference among all levels of evaporation for plant height but significantly vary for other agronomic and yield traits. The fertilizer applications significant enhanced the biological seed yield/plant (5.43Kg) of canola and the highest yield (2450Kg/ha) was recorded with NPK (T_1) under normal irrigation (I_0). The T_1 and T_2 treatment presented a non- significant difference under normal irrigation condition. However, the T_3 treatment significantly increased the grain yield of canola under mild (I_3) and severe water stress (I_4), compared to other treatments. The maximum grain yield was recorded with $T_{1,}$ compared to T_1 under normal irrigation and declined with the severity of water stress. Moreover, data displayed a strong and significant interaction between different rate of evaporation (I) and applied elicitors (T) in all studied features of canola except leaf area (Table 3).

3.2. Physiological performance of canola with fertilizers and PGRs under drought

The mean data of two-year field experiment of canola revealed a highly significant ($p < 0.01$) response of NPK and PGR application to chlorophyll contents under water deficit condition (Table 4). The chlorophyll contents decline significantly in canola with increasing rate of evaporation (I_0-I_4). However, the exogenous application of NPK (T_1) significantly enhanced *Chl a, Chl b* and total chlorophyll contents under control but combination of NPK and PGRs (T_3) progressively recovered the chlorophyll contents from least to severe water stress (I2-I). The *Chl b* contents decreased significantly with progression of water stress, even fortification of NPK and PGRs also failed to mitigate adverse effects of over evaporation. A highly significant relation was observed between different irrigation regimes (I) and treatments with elicitors (T) for chlorophyll contents and also showed a recovery mechanism by promoting photosynthetic activity (Table 4).

The average of two season's data for water contents and temperature of canola leaves showed a significant ($p < 0.01$) interaction of irrigation and elicitors (Table 4). Both these traits worked antagonistically, with the decrease in leaf water content (LWC), leaf temperature (LT) increased during severe drought (I_4) condition. The irrigation regimes I_1 (Control) and I_2 (MRE) showed non-significant difference on water contents and leaf temperature and same effects were observed with chemical fertilizer (T_1) under mild (I_1) and moderate (I_2) rate of evaporation. However, the T_3 treatment increased the LWC by reducing LT of canola under mild (I_0) and severe stressful (I_4) condition (Table 4).

The NPK application significantly ($p < 0.01$) influenced the stomatal conductance of canola plant with different rate of evaporation (Table 4). The water stress reduced the stomatal conductance under severe rate of evaporation (I_4) but exhibited non-significant change under the mild (I_1) and moderate (I_2) rate of evaporation. The combined effect of PGRs and chemical fertilizers (T_3) significantly enhanced the stomatal conductance under normal (I_0) and mild (I_1) water stress. A drastic reduction of stomatal conductance was recorded with increasing water stress. A significant interaction of stomatal conductance (143.4**) was recorded for evaporation and applied treatments (Table 4).

3.3. Qualitative performance of canola with fertilizers and PGRs under drought

The mean of two years statistical data depicted that the interactive effect of irrigation and fertilizers significantly influenced the quality related traits (oil contents, oil yield, protein, glucosinolates, and moisture and erucic acid contents) of canola (Table 5). These features declined with the progression of water deficit condition. The experimental results defined the positive influence of chemical and PGRs (T) on oil yield, oil, protein and moisture contents under normal (I_0) and severe water deficit (I_4) condition, while reduced glucosinolates and erucic acid contents (Table 5). It is summarized that T_3 treatment is a good rehabilitation strategy to improve quality of canola followed by T_1 and T_2 to address water scarcity issues of canola.

3.4. Nitrogen, Phosphorus and Potassium (NPK) contents and their Uptake in canola

The mean data of two consecutive years (2018-19/2019-20) showed that combined effect of fertilizer and PGRs (T_3) significantly ($p < 0.01$) influence the NPK contents and their uptake in canola plant. Provision of chemical fertilizer (T_1) individually or in combination with PGRs (T_3) improved the NPK contents as well as their uptake under mild (I_0) irrigation stress (Table 6).

3.5. Biochemical performance of canola with fertilizers and PGRs under drought

The results obtained from two-year trails illustrated that the osmolytes considerably ($p < 0.01$) influenced by the applied elicitors under different irrigation regimes. The level of proline and soluble sugar elevated in response to mild to severe rate of evaporation canola plant as an innate response mechanism. Further, the NPK and PGRs (T_3) showed a negative impact on the proline accumulation but positively enhanced soluble sugar contents under all level of irrigation (Table 7). The proline and total soluble sugar contents increased and decreased respectively with the application of NPK (T_1) followed by PGRs (T_2) and combined treatment (T_3) under all levels of irrigation (Table 7).

The antioxidant enzymes and MDA activities significantly ($p < 0.01$) influenced by the irrigation (I) and elicitors (T) in canola (Table 7). The canola plant enhanced the activities of various antioxidants and enzymes including SOD, CAT, PPO, POX and MDA contents in response to water stress. The canola behavior with fertilizers did not show significant effect during normal (I_1) and mild irrigation (I_2) stress except lipid peroxidation and POX activity. The T_1 treatment exhibited non-significant difference on enzyme activities but the MDA content influenced significantly under all irrigation levels (I_0-I_4). The treatment T_2 (PGRs) and T_3 (NPK and PGRs)

elevated the activities SOD, PPO, POX and SOD but reduced the MDA contents as compared to T_0 under severe water deficit (I_4) condition. (Table 7).

The interaction of irrigation intervals (I) and elicitors (T) significantly affect the membrane stability index (MSI) of canola ($p < 0.01$). The water stress significantly reduced the membrane stability index. Only chemical fertilizer did not effect on the MSI while the combined fertilizers and plant growth regulators significantly improve the MSI under moderate and severe water stress. The chemical fertilizer only showed superiority under irrigation level of I_3 (Table 7).

4. Discussion

The findings of the current work highlighted the comparison of combined and individual application of PGR and NPK to enhance canola growth, nutritional quality and yield for growing concerns of water scarcity. Particularly moderate to severe drought stress (I_3-I_4) imparts drastic effect on the canola growth by causing injuries at all growth stages. It influenced the various morphological (reduced leaf growth, leaf area, plant height, number of nodes per plant), physiological traits (chlorophyll content, leaf water contents, leaf temperature and stomatal conductance) at the onset of water scarcity (Table 3, 4). Reduction of plant height was recorded in canola under different irrigation regimes compared to exogenously applied PGRs and NPK (Table 3). Growth retardation due to excessive evaporation, can be related to disruption of photosynthetic machinery and decline in carbon reserves for relocation to growing parts of plant [45, 46]. It seems that the decrease in plant height also interferes with leaf area. This reduction is particularly noticeable during post vegetative stage, flowering stage or abscission [46]. The grain yield reduced due to water stress as reported in pervious study [47]. Seed quality and 100 seed weight are commercial as well as economic traits, significantly compromised with ongoing scenario of water scarcity, and so is in the present experiment (Table 3). Seed filling is particularly influenced by drought stress by modulating of various metabolic activities occurring in the leaves, such as synthesis and translocation of photoassimilates, essential substrates for biosynthesis of seed storage reserves, mineral nutrients and many more functional constituents [48].

In the current experiment, drought led to reduction in chlorophyll content, and this loss could be due to some devastating effects on photosynthetic apparatus (Table 4). Stomatal conductance (g_s) was severely hampered when plants were exposed to severe rate of evaporation (I_4). The resistance in stomatal conductance (g_s) may be correlated to enhanced production of ABA

under drought stress, whichleads to stomata closure. ABA signaling mechanism tries to prevent the loss of tissue turgor by closing the stomata [49, 50]. The optimal use of chemical fertilizers and PGR appreciably enhanced stomatal conductance under drought stress (Table 4). Yan et al. [51] also reported diffusional restrictions of CO_2 by stomata (52%), which directly caused a reduction of chlorophyll contents (31%) induced by drought. The fertilizer application especially urea increase the N supply at flowering and pod filling stage, delay leaf aging, enhanced chlorophyll contents and photo assimilates [52]. The total amount of chlorophyll contents increased by the availability of nitro compounds in the rhizosphere and consequently to the plants, ultimately produced more assimilates via photosynthesis which directly related to improve growth and yield [13, 53]. Growth regulators and chemical fertilizers were significantly effective in mitigating the drastic effects of drought by maintaining the water efficiency of canola plants and augmenting the accretion of osmolytes. Accumulation of osmolytes may also favors the improvement of photosynthetic and gas exchange attributes [19].

Canola oil is the commercial commodity, while its content, profile and composition are affected by drought stress as reported in the current work (Table 5). Seed oil stems mostly from photosynthesis and green silique walls, later carbon is routed through different metabolic pathways into triacylglycerol occurring in the chloroplast, cytosol, and endoplasmic reticula [54]. The current experiment suggests 3–11% decrease in oil content and oil yield in the *B. napus* when the plants were exposed to irrigation stress, but Aslam et al. [55] reported a mere reduction of 3.2%.

The NPK treatment increases the flow of nutrient to the aerial part of plants and reduced the impact of water stress (Table 6). Due to water scarcity and loss of ionic balance, nutrients remained bond to the soil particles that are critical for the normal growth and development [56, 57]. Same findings have been reported in the present work in the form of decreased biological yield (40%) with non-availability of nutrients (5-10%). Particularly, Nitrogen (N), phosphorus (P) and potassium (K) contents increased with foliarly applied PGRs followed by chemical fertilizers in canola. This might be accredited to the role of K in biochemical pathways in plants. Potassium has a positive effects on metabolic processes of nucleic acids and proteins [58]. Phosphorus as a constituent of cell nuclei is essential for cell division and development of meristematic tissue of cotton. Further, P has a well-known impact in photosynthesis as well as synthesis of nucleic acids, proteins, lipids and other essential compounds [59]. The percentage of NPK uptake enhanced with the combined

application of NPK and PGRs (Table 5). The bio fertilizers improve the soil textures and bacterial colonization with the modification of physio-chemical properties of rhizosphere. On the other hand, the PGRs increased the photo assimilates translocation to sink (root tissue) and also improved the nutrient uptake and absorption power under water deficit and adverse environment condition. The PGRs can enhanced the activity of some vital N, P and K metabolizing enzymes in plant which enhanced NPK contents under different irrigation regimes [53, 60].

All plants have been equipped with innate antioxidant enzyme mechanisms for the detoxification of reactive oxygen species. CAT decomposes H_2O_2 into water and molecular O_2. POX dismutases H_2O_2 by oxidizing co-substrates such as phenolic compounds and/or antioxidants and PPO in urn oxidizes phenols to chinone [61].

Membrane lipid peroxidation is a frequently used indictor to test the degree of plant sensitivity to oxidative damage caused by stress [62]. Our data revealed sensitivity of canola towards drought by elevation MDA content compared to F_2, F_3 treatments (Table 7). A lower level of lipid peroxidation presented high membrane stability (Table 7). It seems that the cell membrane integrity was maintained with chemical fertilizers and PGRs against the oxidative stress induced by water stress (Table 7). Mamnabi et al. [13] also suggested an amplification of antioxidant enzymes, total soluble sugars, photosynthetic attributes, leaf water content, membrane stability index and stomatal conductance but decreased the leaf temperature under different irrigation regimes.

The affirmative role of fertilizers and PGRs increase the antioxidant enzyme activities under water deficient condition in canola [63]. The PGRs treated plant showed more activities of antioxidant enzymes like POD, CAT and PPO as compared to untreated plants under moderate and severe water stress. The highest antioxidant activities were observed in T_3 treatments under severe stress as compared to T_1 and T_2 (Table 7). This supremacy was attained by the additive effect of fertilizer and PGRS on canola plants.

Conclusion

Canola is an emerging and unique oil seed crop among the other oil producing plants due to low erucic acid and glucosinolates contents. Here, we initially illustrate how NPK and PGRs, either individually or in combination, impact canola growth, photosynthetic and antioxidant activities

and later seed yield and quality, and also attempt to explain its interaction to water scarcity for addressing these vital challenges. From the outcomes of current study, it appears rational to recommend chemical fertilizers (NPK) and PGRs (IAA and GA_3), that brought about better impact on canola seed yield, seed protein content, oil, oil composition with low glucosinolate and euracic acid contents, even under severe rate of evaporation (150mm). The harmful effects of stress were minimized considerably by the combined application of fertilizers and PGRs, thus improved growth attributes, chlorophyll content, MSI, stomatal conductance, antioxidants, osmoprotectants, grain yield and importantly, leading to a reduction in lipid peroxidation, particularly under moderate and severe rate of evaporation. These supremacies were attained by additive effects of NPK and PGPR, reducing the impact of drought. Such models can improve the probability of forecasting canola aptitude in challenging climates with an immediate response, but will also broadly help to select traits that can be further exploited through gene mining to produce sustainable and climate-resilient canola genotypes with considerable yield under high rate of water evaporation.

Author Contribution/s

Mohammad Aquil Siddiqui & Muhammad Mahran Aslam conceive the ideal, conducted the experiment, statistical analysis. Fozia Farhat wrote first draft of the manuscript and finalized it. Shafquat Yasmeen and Muhammad Tahir Khan helped in collecting the agronomic data of the crop and formatting the paper. Mahboob Ali Sial & Imtiaz Ahmad Khan review the draft and provided technical inputs.

References

1. Anonymous. Food Outlook; Global Information and Early Warning System. FAO. Rome, Italy, 2015.
2. Mohtashami R, Movahhedi Dehnavi M, Balouchi H, Faraji H. Improving yield, oil content and water productivity of dryland canola by supplementary irrigation and selenium spraying. Agric. Water Manag. 2020; 232: 106046.
3.Anonymous. Rapeseed crop production. FAO. Rome, Italy, 2017. http://www. fao.org/ faostat/en/#data/QC.2014;Crops/Regions/Worldlist/ProductionQuantity
4. Walker KC, Booth EJ. Agricultural aspects of rape and other Brassica products. Eur. J. Lipid Sci. Technol. 2001; 103(7): 441–446.
5. Jahangir M, Abdel-Farid IB, Simoh S, Kim HK, Verpoorte R, Choi YH. Investigation of Brassica biochemical status by NMR-based metabolomics. *XXVIII International Horticultural Congress on Science and Horticulture for People (IHC2010): Int. Symposium* . 2010; 936*:* 163–172.
6. Mitrović PM, Stamenković OS, Banković-Ilić I, Djalović IG, Nježić ZB, et al. White Mustard (Sinapis alba L.) Oil in Biodiesel Production: A Review. Front. plant sci. 2020; 11: 299-299.
7. Elferjani R, Soolanayakanahally R. Canola responses to drought, heat, and combined stress: shared and specific effects on carbon assimilation, seed yield, and oil composition. Front. Plant Sci. 2018: 1–17. https://doi.org/10.3389/fpls.2018.01224
8. Champolivier L, Merrien A. Effects of water stress applied at different growth stages to Brassica napus L. var. oleifera on yield, yield components and seed quality. Eur. J. Agron.. 1996; 5(3–4): 153–160.
9. Aksouh-Harradj NM, Campbell LC, Mailer RJ. Canola response to high and moderately high temperature stresses during seed maturation. Canadian Journal of Plant Science. 2006; 86(4): 967–980.
10. Ahmar S, Liaqat N, Hussain M, Salim MA, Shabbir MA, Ali MY, Noushahi HA, Bilal M, Atta B, Rizwan M. Effect of abiotic stresses on Brassica species and role of transgenic breeding for adaptation. Asian J. Res. Crop Sci. 2019: 1–10.
11. Hussain HA, Hussain S, Khaliq A, Ashraf U, Anjum SA, et al. Chilling and Drought Stresses in Crop Plants: Implications, Cross Talk, and Potential Management Opportunities. Front. plant sci. 2018; 9: 393-393.
12. Urban L, Aarrouf J, Bidel LPR. Assessing the Effects of Water Deficit on Photosynthesis Using Parameters Derived from Measurements of Leaf Gas Exchange and of Chlorophyll a Fluorescence. Front. plant sci. 2017; 8: 2068-2068.
13. Mamnabi S, Nasrollahzadeh S, Ghassemi-Golezani K, Raei Y. Improving yield-related physiological characteristics of spring rapeseed by integrated fertilizer management under water deficit conditions. Saudi J. Biol. Sci. 2020; 27: 797-804.
14. Yin Z, Lu J, Meng S, Liu Y, Mostafa I, et al. Exogenous melatonin improves salt tolerance in tomato by regulating photosynthetic electron flux and the ascorbate–glutathione cycle. J. Plant. Interact. 2019; 14: 453-463.
15. Das K, Roychoudhury A. Reactive oxygen species (ROS) and response of antioxidants as ROS-scavengers during environmental stress in plants. Front. Environ. Sci. 2014: 2.

16. Rezayian M, Ebrahimzadeh H, Niknam V. Nitric Oxide Stimulates Antioxidant System and Osmotic Adjustment in Soybean Under Drought Stress. J. Soil Sci. Plant. Nutr. 2020; 20: 1122-1132.
17. Khaleghi A, Naderi R, Brunetti C, Maserti BE, Salami SA, et al. Morphological, physiochemical and antioxidant responses of Maclura pomifera to drought stress. Sci. Rep. 2019; 9: 19250.
18. Laxa M, Liebthal M, Telman W, Chibani K, Dietz K-J. The Role of the Plant Antioxidant System in Drought Tolerance. Antioxidants (Basel, Switzerland). 2019; 8: 94.
19. Sharma A, Shahzad B, Kumar V, Kohli SK, Sidhu GPS, et al. Phytohormones Regulate Accumulation of Osmolytes Under Abiotic Stress. Biomolecules. 2019; 9: 285.
20. Hasanuzzaman M, Bhuyan MHMB, Zulfiqar F, Raza A, Mohsin SM, et al. Reactive Oxygen Species and Antioxidant Defense in Plants under Abiotic Stress: Revisiting the Crucial Role of a Universal Defense Regulator. Antioxidants. 2020; 9: 681.
21. Khattab EA, Afifi MH, Amin GA. Significance of nitrogen, phosphorus, and boron foliar spray on jojoba plants. Bull. Nati Res. Cen. 2019; 43: 66.
22. Anjum NA, Gill SS, Umar S, Ahmad I, Duarte AC, et al. Improving Growth and Productivity of Oleiferous Brassicas under Changing Environment: Significance of Nitrogen and Sulphur Nutrition, and Underlying Mechanisms. Sci. World J. 2012: 657808.
23. Yagoub SO, Ahmed WMA, Mariod. Effect of Urea, NPK and Compost on Growth and Yield of Soybean (Glycine max L.), in Semi-Arid Region of Sudan. ISRN Agronomy 2012: 678124.
24. Shirazi SM, Yusop Z, Zardari NH, Ismail Z. Effect of Irrigation Regimes and Nitrogen Levels on the Growth and Yield of Wheat. Adv. Agric. 2014: 250874.
25. Manik SMN, Pengilley G, Dean G, Field B, Shabala S, et al. Soil and Crop Management Practices to Minimize the Impact of Waterlogging on Crop Productivity. Front. plant sci. 2019; 10: 140-140.
26. Rezaei R, Valadabadi SAR, Shirani Rad AH, Sayfzadeh S, Hadidi Masouleh E. Effects of biological fertilizers application and various amounts of urea fertilizers under water stress conditions on yield, nitrogen use efficiency and effective ingredients of Calendula officinalis L. Iranian. J. Med. Aromat. Plants Res. 2018; 34: 547-564.
27. Bhardwaj D, Ansari MW, Sahoo RK, Tuteja N. Biofertilizers function as key player in sustainable agriculture by improving soil fertility, plant tolerance and crop productivity. Microb. cell. fact. 2014; 13: 66-66.
28. Mondal T, Datta J, Mondal N. Chemical fertilizer in conjunction with biofertilizer and vermicompost induced changes in morpho-physiological and bio-chemical traits of mustard crop. J. Saudi Soc. Agric. Sci. 2017; 16: 135-144.
29. Sevinç K. Effects of Vermicompost on Some Morphological, Physiological and Biochemical Parameters of Lettuce (Lactuca sativa var. crispa) under Drought Stress. Not Bot Horti Agrobot Cluj Napoca. 2018: 47.
30. Mukhongo RW, Tumuhairwe JB, Ebanyat P, AbdelGadir AH, Thuita M, et al. Combined Application of Biofertilizers and Inorganic Nutrients Improves Sweet Potato Yields. Front. Plant. Sci. 2017: 8.
31. Stalikas CD. Extraction, separation, and detection methods for phenolic acids and flavonoids. J. Sep. Sci. 2017; 30: 3268-3295.

32. Rathje. Jackson, M. L.: Soil chemical analysis. Verlag: Prentice Hall, Inc., Englewood Cliffs, NJ. 1958, 498 S. DM 39.40. Zeitschrift für Pflanzenernährung, Düngung, Bodenkunde. 1959; 85: 251-252.
33. Pimstein A, Karnieli A, Bansal SK, Bonfil DJ. Exploring remotely sensed technologies for monitoring wheat potassium and phosphorus using field spectroscopy. Field. Crops. Res. 2011; 121: 125-135.
34. Tammam AA. Study of salt tolerance in wheat (*Triticum aestium* L.) cultivar Banysoif 1. Australian journal of crop science. 2008; 1:115-125.
35.Bremner JM, Mulvaney CS. Nitrogen total. P.595-62. In: Methods of Soil Analysis. Part 2: 2nd edition. A.L. Page, R.H. Miller and D.R. Keeney (eds.) American Society of Agronomy, Madison, WI. USA. 1982.
36. Arnon DI. Copper enzymes in isolated chloroplasts. Polyphenoloxidase in beta vulgaris. Plant physiol. 1949; 24: 1-15.
37. Ghassemi-Golezani K, Ghassemi, S., Yaghoubian, I. Salicylic Acid regulate Physiological Performance of milk thistle (Silybum marianum L.) under water stress. Adv Biores. 2016; 7(4): 34-40.
38. Kochert G. Carbohydrate determination by the phenol-sulfuric acid method. Handbook. Phycol Meth. 1978: 95-97.
39. Bates LS, Waldren RP, Teare ID. Rapid determination of free proline for water-stress studies. Plant and Soil. 1973; 39: 205-207.
40. Kumar KB, Khan, P.A. Peroxidase and polyphenoloxidase in excisedragi (Eleusine coracana cv. PR 202) leaves during senescence. Indian J. Exp. Biol. 1982: 412–416.
41. Singh BK, Sharma SR, Singh B. Antioxidant enzymes in cabbage: Variability and inheritance of superoxide dismutase, peroxidase and catalase. Scientia Horticulturae. 2010; 124: 9-13.
42. Gueta-Dahan Y, Yaniv Z, Zilinskas BA, Ben-Hayyim G. Salt and oxidative stress: similar and specific responses and their relation to salt tolerance in citrus. Planta. 1997; 203: 460-469.
43. Janero DR. Malondialdehyde and thiobarbituric acid-reactivity as diagnostic indices of lipid peroxidation and peroxidative tissue injury. Free Radic Biol Med. 1990; 9: 515-540.
44. Gomez KAaG, A. Statistical Procedure for Agricultural Research—Hand Book. John Wiley & Sons New york. 1984.
45. Shahid MA, Sarkhosh A, Khan N, Balal RM, Ali S, et al. Insights into the Physiological and Biochemical Impacts of Salt Stress on Plant Growth and Development. Agronomy. 2020; 10: 938.
46. Sharif P, Seyedsalehi M, Paladino O, Van Damme P, Sillanpää M, et al. Effect of drought and salinity stresses on morphological and physiological characteristics of canola. Int. J. Environ. Sci. Technol. 2018; 15: 1859-1866.
47. Flexas J, Medrano H. Drought-inhibition of photosynthesis in C3 plants: stomatal and non-stomatal limitations revisited. Ann. Bot. 2002; 89: 183-189.
48. Sehgal, A., Sita, K., Siddique, K. H. M., Kumar, R., Bhogireddy, S., Varshney, R. K., HanumanthaRao, B., Nair, R. M., Prasad, P. V. V., & Nayyar, H. (2018). Drought or/and heat-stress effects on seed filling in food crops: Impacts on functional biochemistry, seed yields, and nutritional quality. *Frontiers in Plant Science*, *871*(November), 1–19. https://doi.org/10.3389/fpls.2018.01705.

49. Wilkinson S, Davies WJ. Drought, ozone, ABA and ethylene: new insights from cell to plant to community. Plant Cell Environ. 2010; 33: 510-525.

50. Pantin F, Monnet F, Jannaud D, Costa JM, Renaud J, et al. The dual effect of abscisic acid on stomata. New Phytol. 2013; 197: 65-72.
51. Yan W, Zhong Y, Shangguan Z. A meta-analysis of leaf gas exchange and water status responses to drought. Sci. Rep. 2016; 6: 20917.
52. Gai Z, Zhang J, Li C. Effects of starter nitrogen fertilizer on soybean root activity, leaf photosynthesis and grain yield. PLoS ONE. 2017. 12: 0174841.
53. Razaq M, Zhang P, Shen H-l, Salahuddin. Influence of nitrogen and phosphorous on the growth and root morphology of Acer mono. PLoS ONE. 2017; 12: 0171321.
54. Baud S, Lepiniec L. Physiological and developmental regulation of seed oil production. Prog. Lipid Res. 2010; 49: 235-249.
55. Aslam MN, Nelson MN, Kailis SG, Bayliss KL, Speijers J, et al. Canola oil increases in polyunsaturated fatty acids and decreases in oleic acid in drought-stressed Mediterranean-type environments. Plant Breed. 2009; 128: 348-355.
56. Elferjani R, Soolanayakanahally R. Canola Responses to Drought, Heat, and Combined Stress: Shared and Specific Effects on Carbon Assimilation, Seed Yield, and Oil Composition. Front. Plant Sci. 2018; 30(9):1224.
57. Barnabás B, Jäger K, Fehér A. The effect of drought and heat stress on reproductive processes in cereals. Plant Cell Environ. 2008; 31: 11-38.
58. Bednarz CW, Oosterhuis DM. Physiological changes associated with potassium deficiency in cotton. J. Plant Nutr. 1999; 22(2): 303–313.
59. Sawana ZM, Hafez SA, Alkassas AR . Nitrogen, potassium and plant growth retardant effects on oil content and quality of cotton seed. Grasas Y Aceites. 2007; 58(3): 243–251.
60. Waqas MA, Khan I, Akhter MJ, Noor MA, Ashraf U. Exogenous application of plant growth regulators (PGRs) induces chilling tolerance in short-duration hybrid maize. Environ. Sci. Pollut. Res. 2017; 24: 11459-11471.
61. Apel K, Hirt H. Reactive oxygen species: metabolism, oxidative stress, and signal transduction. Annu. Rev. Plant Biol. 2004; 55: 373-399.
62. Blokhina O, Virolainen E, Fagerstedt KV. Antioxidants, oxidative damage and oxygen deprivation stress: a review. Ann. Bot. 2004; 91: 179-194.
63. Khan N, Bano A, Babar MDA. Impacts of plant growth promoters and plant growth regulators on rainfed agriculture. PloS one. 2020; 15: e0231426-e0231426.

Table 1: Weather data of experimental location during 2016-17

**Month**	**T_{max} ^{0}C**		**T_{min} ^{0}C**		**Total Rain fall (mm)**		**Relative Humidity (%)**	
	2018-19	2019-20	2018-19	2019-20	2018-19	2019-20	2018-19	2019-20
**October**	37.1	38.4	20.6	19.5	0	0	57	53
**November**	32.6	31.20	12.9	12.6	0	0	52	51
**December**	28.5	25.6	11.2	8.0	0	0	56	54
**January**	25.4	22.1	10.3	7.3	0	2.0	60	61
**February**	21.4	28.2	9.2	10.0	0	0	44	49
**March**	34.1	34.4	17.1	14.5	0	0	47	47
**April**	39.0	40.4	20.9	19.9	0	0	43	42

Table 2: **Soil properties of experimental Field**

**Site**	**OM (%)**	**pH**	**meq/100g soil**				**µg/g soil**			
**NIA, Tandojam Exp. Farm**	2	6.5-7.0	Na	K	Mg	S	Zn	B	P	Ca
			0.08	0.11	3.2	16.90	1.40	0.34	10.83	2.5

Table 3: Variation in agronomical and yield attributes of Canola with fertilizers and Plant growth regulators under different water regimes

Irrigation	Treatment with Elicitors	Plant Height (cm)	Number of branches/plant	Leaf Area (cm^2)	Days to Flower	Number of seeds/plant	Biological yield/Plant (Kg)	1000 seed weight(g)	Seed yield (Kg/ha)	Grain Yield (gr/m)
**I_1 Normal**	T_0	114^{c}	5.60^{d}	90.5^{d}	68^{b}	160^{d}	5.30^{d}	3.98^{d}	1804^{d}	188^{g}
	T_1	114.5^{b}	5.73^{c}	91.3^{c}	68^{b}	171^{c}	5.38^{c}	4.28^{c}	2073^{b}	264^{a}
	T_2	115^{a}	5.78^{b}	92.4^{b}	69^{a}	176^{b}	5.36^{b}	4.33^{b}	2060^{c}	220^{d}
	T_3	113^{d}	5.85^{a}	94.0^{a}	66^{c}	188^{a}	5.43^{a}	4.96^{a}	2450^{a}	249^{b}
**I_2 Mild evaporation**	T_0	113^{b}	5.66^{d}	89.0^{d}	70^{a}	164^{d}	5.6^{a}	4.0^{d}	1800^{d}	176.4^{h}
	T_1	114^{a}	5.81^{b}	90.0^{c}	67^{c}	170^{b}	5.20^{b}	4.15^{c}	2096^{c}	232.42^{c}
	T_2	114^{a}	5.80^{c}	90.5^{b}	68^{b}	169^{c}	5.16^{d}	4.19^{b}	2118^{b}	207.24^{e}
	T_3	113^{b}	5.83^{a}	91.0^{a}	65^{d}	178^{a}	5.29^{c}	4.23^{a}	2340^{a}	221.18^{bc}
**I_3 Moderate evaporation**	T_0	112^{b}	5.58^{d}	86.4^{d}	70^{a}	165^{d}	4.94^{d}	3.90^{d}	1650^{e}	131.06^{k}
	T_1	112^{b}	5.78^{c}	89.5^{c}	65^{c}	169^{c}	5.14^{c}	4.18^{b}	1943^{b}	165.97^{i}
	T_2	113^{a}	5.80^{b}	90.0^{b}	67^{b}	172^{b}	5.19^{b}	4.09^{c}	1940^{c}	172.03^{h}
	T_3	112^{b}	5.82^{a}	93.0^{a}	64^{d}	178^{a}	5.27^{a}	4.26^{a}	2353^{a}	198.21^{f}
**I_4 Severe evaporation**	T_0	113^{a}	5.52^{d}	85.8^{d}	69^{a}	150^{d}	3.89^{d}	3.67^{d}	1538^{f}	170.52^{h}
	T_1	112^{b}	5.68^{c}	88.4^{b}	67^{c}	168^{b}	5.16^{b}	4.13^{c}	1923^{c}	173.23^{h}
	T_2	113^{a}	5.70^{b}	87.0^{c}	68^{b}	163^{c}	5.14^{c}	4.15^{b}	1975^{b}	133.05^{k}
	T_3	111^{c}	5.79^{a}	92.3^{a}	62^{d}	176^{a}	5.32^{a}	4.26^{a}	2318^{a}	156.84^{j}
**F_{test}**	I × T	1.23*	2.65**	0.023ns	7.75*	68.16**	1.27**	0.53*	30.64**	1803.26**

Note: I_1= 60mm evaporation, I_2= 100mm evaporation, I_3= 120mm evaporation, I_4= 150mm evaporation, T_0= No treatment, T_1= NPK, T_2= PGRs, T_3= (T_1+T_2). The alphabetical superscript in a column present significant difference among the treatments to different rate of evaporation from highest to lowest value (a=highest value). *= least significant, **significant, *** highly significant

Table 4: Variation in Physiological attributes of Canola with chemical fertilizers and Plant growth regulators under different water regimes

Irrigation	Treatments	Chl a	Chl b	Total Chlorophyll	LWC	LT	Stomatal Conductance
		(mg/g FW)			(%)	(^{0}C)	(mmol m-^{2} s^{-1})
**I_1 Normal**	T_0	1.43^{f}	0.834^{c}	2.264^{d}	80.0^{ab}	21.8^{i}	142.3^{c}
	T_1	2.18^{a}	0.87^{a}	3.05^{a}	83.0^{a}	19.7^{l}	146.2^{a}
	T_2	1.63^{e}	0.84^{b}	2.47^{d}	82.0^{a}	21.6^{j}	$144.\ 1^{b}$
	T_3	1.95^{b}	0.85^{b}	2.8^{c}	81.9^{a}	19.8^{k}	146.42^{a}
**I_2 Mild evaporation**	T_0	1.28^{h}	0.78^{d}	2.06^{f}	80.82^{ab}	25.6^{f}	140.73^{d}
	T_1	1.88^{c}	0.82^{c}	2.7^{c}	81.80^{a}	22.3^{h}	145.12^{a}
	T_2	1.48^{f}	0.80^{c}	2.28^{d}	81.17^{a}	23.5^{g}	144.95^{b}
	T_3	1.72^{d}	0.80^{c}	2.52^{b}	82.0^{a}	21.8^{i}	143.91^{b}
**I_3 Moderate evaporation**	T_0	0.73^{k}	0.72^{f}	1.45^{i}	74.17^{de}	30.6^{c}	101.0^{i}
	T_1	1.18^{i}	0.75^{e}	1.93^{g}	76.16^{cd}	28.5^{d}	107.9^{h}
	T_2	1.18^{i}	0.75^{e}	1.93^{g}	78.18^{bc}	27.6^{e}	117.26^{f}
	T_3	1.39^{g}	0.76^{e}	2.15^{e}	78.84^{bc}	25.91^{f}	119.47^{e}
**I_4 Severe evaporation**	T_0	0.47^{m}	0.68^{g}	1.15^{l}	62.49^{f}	35.0^{a}	70.94^{k}
	T_1	0.66^{l}	0.71^{f}	1.37^{k}	65.18^{f}	33.4^{b}	78.61^{j}
	T_2	0.85^{k}	0.70^{f}	1.55^{j}	71.80^{e}	28.92^{d}	97.67^{i}
	T_3	1.13^{j}	0.72^{f}	1.85^{h}	73.6^{de}	27.9^{e}	110.4^{g}
**F_{test}**	I × T	0.134**	0.00032**	1.982**	0.139**	21.99**	143.4**

Note: I_1= 60mm evaporation, I_2= 100mm evaporation, I_3= 120mm evaporation, I_4= 150mm evaporation, T_0= No treatment, T_1= NPK, T_2= PGRs, T_3= (T_1+T_2). The alphabetical superscript in a column present significant difference among the treatments to different rate of evaporation from highest to lowest value (a=highest value). *= least significant, **significant, *** highly significant

Table 5: Variation in quality traits of Canola with chemical fertilizers and Plant growth regulators under different water regimes

Irrigation	Treatments	Oil content (%)	Oil yield (kg ha^{1})	Protein (%)	Moisture (%)	Glucosionalate (µmol/g)	Erucic acid (%)
**I_1 Normal**	T_0	38.0^{d}	238.0^{d}	20.77^{d}	5.23^{d}	26.2^{a}	4.44^{a}
	T_1	38.5^{c}	324.5^{c}	24.82^{c}	5.72^{c}	22.0^{c}	4.20^{b}
	T_2	39.0^{b}	328.9^{b}	24.97^{b}	5.96^{b}	18.7^{d}	3.74^{c}
	T_3	42.8^{a}	468.0^{a}	25.07^{a}	6.77^{a}	14.6^{f}	3.22^{d}
**I_2 Mild evaporation**	T_0	37.0^{d}	234.6^{d}	20.0^{d}	5.0^{d}	24.0^{b}	5.21^{a}
	T_1	37.8^{c}	340.0^{c}	24.82^{c}	5.36^{b}	20.0^{c}	4.37^{b}
	T_2	38.2^{b}	355.0^{b}	24.79^{b}	5.30^{c}	19.0^{d}	3.98^{c}
	T_3	42.5^{a}	437.8^{a}	25.0^{a}	5.98^{a}	16.0^{e}	3.41^{d}
**I_3 Moderate evaporation**	T_0	36.0^{d}	229.0^{d}	18.5^{d}	4.93^{c}	23.5^{b}	4.98^{a}
	T_1	37.3^{c}	324.5^{c}	23.0^{c}	5.11^{c}	17.0^{e}	3.75^{c}
	T_2	37.9^{b}	329.38^{b}	23.5^{b}	5.19^{b}	18.2^{d}	3.82^{b}
	T_3	41.6^{a}	398.0^{a}	24.8^{a}	5.75^{a}	14.0^{f}	3.45^{d}
**I_4 Severe evaporation**	T_0	34.8^{d}	220.0^{d}	18.0^{d}	4.81^{d}	20.0^{c}	4.99^{a}
	T_1	36.0^{c}	378.3^{c}	23.0^{b}	5.25^{c}	18.0^{d}	3.68^{c}
	T_2	36.5^{b}	382.0^{b}	22.5^{c}	5.29^{b}	16.5^{e}	3.71^{b}
	T_3	41.0^{a}	437.2^{a}	24.2^{a}	5.78^{a}	11.6^{g}	3.50^{d}
**F_{test}**	I × T	13.68**	6.34*	3.36**	0.985**	4.28*	12.78**

Note: I_1= 60mm evaporation, I_2= 100mm evaporation, I_3= 120mm evaporation, I_4= 150mm evaporation, T_0= No treatment, T_1= NPK, T_2= PGRs, T_3= (T_1+T_2). The alphabetical superscript in a column present significant difference among the treatments to different rate of evaporation from highest to lowest value (a=highest value). *= least significant, **significant, *** highly significant

Table 6: Variation in Nitrogen (N), Phosphorus (P) and Potassium (K) of Canola with chemical fertilizers and Plant growth regulators under different water regimes

Irrigation	Treatments	N (%)	P (%)	K (%)	N uptake (kg ha^{1})	P uptake (kg ha^{1})	K uptake (kg ha^{1})
**I_1 Normal**	T_0	2.70^{d}	0.18^{d}	1.21^{d}	22.81^{d}	17.43^{d}	16.34^{d}
	T_1	3.72^{b}	0.21^{b}	1.68^{b}	38.45^{b}	21.09^{b}	18.23^{b}
	T_2	3.59^{c}	0.20^{c}	1.55^{c}	34.23^{c}	19.94^{c}	17.78^{c}
	T_3	3.88^{a}	0.24^{a}	1.88^{a}	51.84^{a}	23.29^{a}	22.67^{a}
**I_2 Mild evaporation**	T_0	2.69^{d}	0.20^{c}	1.20^{d}	22.86^{d}	18.43^{d}	16.0^{d}
	T_1	3.35^{c}	0.20^{c}	1.55^{b}	38.98^{b}	20.45^{b}	18.45^{b}
	T_2	3.54^{b}	0.21^{b}	1.43^{c}	29.74^{c}	19.90^{c}	18.21^{c}
	T_3	3.75^{a}	0.22^{a}	1.78^{a}	50.95^{a}	22.45^{a}	21.45^{a}
**I_3 Moderate evaporation**	T_0	2.71^{d}	0.19^{c}	1.16^{d}	20.09^{d}	17.09^{d}	14.76^{d}
	T_1	3.15^{c}	0.22^{a}	1.61^{b}	30.56^{b}	19.90^{b}	17.85^{c}
	T_2	3.19^{b}	0.21^{b}	1.34^{c}	26.64^{c}	18.64^{c}	17.93^{b}
	T_3	3.84^{a}	0.21^{b}	1.72^{a}	49.80^{a}	21.98^{a}	20.06^{a}
**I_4 Severe evaporation**	T_0	2.55^{d}	0.17^{c}	1.03^{d}	17.65^{d}	15.43^{d}	13.49^{d}
	T_1	3.38^{c}	0.21^{b}	1.45^{c}	30.32^{c}	18.45^{c}	17.83^{c}
	T_2	3.45^{b}	0.21^{b}	1.54^{b}	33.90^{b}	19.05^{b}	16.98^{b}
	T_3	3.78^{a}	0.24^{a}	1.69^{a}	48.73^{a}	20.97^{a}	19.56^{a}
**F_{test}**	I × T	3.05**	0.168***	0.98**	16.24*	9.34***	3.44**

Note: I_1= 60mm evaporation, I_2= 100mm evaporation, I_3= 120mm evaporation, I_4= 150mm evaporation, T_0= No treatment, T_1= NPK, T_2= PGRs, T_3= (T_1+T_2). The alphabetical superscript in a column present significant difference among the treatments to different rate of evaporation from highest to lowest value (a=highest value). *= least significant, **significant, *** highly significant

Table 7: Variation in Biochemical attributes of Canola with chemical fertilizers and Plant growth regulators under different water regimes

Irrigation	Treatments	PPO	CAT	POX ($U\ g^{-1}$ FW)	SOD	MDA ($mmol\ g^{-1}$ FW)	Soluble Sugar (mg /g DW)	Proline ($mmol\ g^{-1}$ FW)	MSI
I_1 Normal	T_0	0.47^{f}	0.21^{h}	0.15^{f}	0.26^{g}	2.4^{j}	31.4^{ijk}	15.4^{ij}	86.71^{b}
	T_1	0.54^{f}	0.23^{h}	0.17^{f}	0.31^{fg}	2.29^{j}	30.0^{k}	16^{ij}	87.92^{a}
	T_2	0.52^{f}	0.26^{h}	0.16^{f}	0.33^{fg}	2.28^{j}	31.0^{ijk}	15.03^{ij}	87.81^{a}
	T_3	0.57^{f}	0.24^{h}	0.18^{f}	0.32^{fg}	2.27^{j}	30.2^{jk}	15.07^{ij}	87.45^{a}
I_2 Mild evaporation	T_0	0.79^{f}	0.6^{gh}	o.36^{ef}	0.59^{efg}	3.5^{gh}	32.8^{ij}	16.36^{hi}	85.0^{c}
	T_1	0.80^{f}	0.64^{g}	0.88^{e}	0.63^{efg}	3.07^{hi}	31.63^{ijk}	16.1^{ij}	87. 25^{ab}
	T_2	1.02^{f}	0.65^{g}	0.95^{e}	0.74^{e}	2.66^{ij}	32.24^{ijk}	15.8^{ij}	88.0^{a}
	T_3	1.08^{f}	1.38^{fg}	1.57^{d}	o.79^{e}	2.44^{j}	32.87^{i}	15.57^{ij}	88.07^{a}
I_3 Moderate evaporation	T_0	1.88^{e}	2.0^{f}	1.67^{d}	1.28^{d}	5.9^{bc}	38.56^{h}	22.8^{c}	75.62^{g}
	T_1	2.03^{e}	2.08^{f}	1.86^{d}	1.31^{d}	5.29^{ed}	43.6^{f}	21.9^{bc}	78.45^{f}
	T_2	2.82^{cd}	3.15^{d}	2.69^{c}	2.10^{c}	4.34^{f}	45.64^{ef}	18.14^{fg}	80.05^{e}
	T_3	3.38^{bc}	3.56^{c}	2.87^{c}	2.18^{c}	3.72^{g}	49.07^{d}	17.34^{gh}	81.98^{d}
I_4 Severe evaporation	T_0	1.9^{e}	2.75^{e}	2.84^{c}	1.96^{c}	7.30^{a}	41.0^{g}	32.68^{a}	64.09^{i}
	T_1	2.15^{e}	2.85^{e}	3.12^{bc}	1.90^{c}	6.34^{b}	43.52^{f}	27.44^{b}	65.32^{i}
	T_2	3.63^{b}	4.07^{b}	3.85^{b}	2.95^{b}	5.57^{cd}	63.36^{b}	22.08^{cd}	70.29^{h}
	T_3	4.19^{a}	4.39^{a}	4.44^{a}	3.44^{a}	5.72^{cd}	67.62^{a}	21.4^{de}	74.49^{g}
F_{test}	I × T	0.612**	0.49**	0.317**	0.34**	0.509**	99.01**	14.49**	14.22**

Note: I_1= 60mm evaporation, I_2= 100mm evaporation, I_3= 120mm evaporation, I_4= 150mm evaporation, T_0= No treatment, T_1= NPK, T_2= PGRs, T_3= (T_1+T_2). The alphabetical superscript in a column present significant difference among the treatments to different rate of evaporation from highest to lowest value (a=highest value). *= least significant, **significant, *** highly significant

Figure
Click here to access/download;Figure;Table_Figures_Canola.docx

Physiological and Biochemical Exploration of Canola for Overcoming Yield Limitation by Integrated Fertilizer management under different water regimes

Table 1: Weather data of experimental location during 2016-17

**Month**	**T_{max} ^0C**		**T_{min} ^0C**		**Total Rain fall (mm)**		**R.H (%)**	
	2016	2017	2016	2017	2016	2017	2016	2017
**October**	37.1	38.4	20.6	19.5	0	0	57	53
**November**	32.6	31.20	12.9	12.6	0	0	52	51
**December**	28.5	25.6	11.2	8.0	0	0	56	54
**January**	25.4	22.1	10.3	7.3	0	2.0	60	61
**February**	21.4	28.2	9.2	10.0	0	0	44	49
**March**	34.1	34.4	17.1	14.5	0	0	47	47
**April**	39.0	40.4	20.9	19.9	0	0	43	42

Table 2: **Soil properties of experimental Field**

**Site**	**OM (%)**	**pH**	**meq/100g soil**				**ug/g soil**			
**NIA, Tandojam Exp. Farm**	2	5.42	Na	K	Mg	S	Zn	B	P	Ca
			0.08	0.11	3.2	16.90	1.40	0.34	10.83	2.5

Table 3: Variation in agronomical and yield attributes of Canola with fertilizers and Plant growth regulators under different water regimes

Irrigation	Treatment with Elicitors	Plant Height (cm)	Number of branches/plant	Leaf Area	Days to Flower	Number of seeds/plant	Biological yield/Plant (Kg)	1000 seed weight(g)	Seed yield (Kg/ha)
**I_1**	T_0	114^{c}	5.60^{d}	90.5^{d}	68^{b}	160^{d}	5.30^{d}	3.98^{d}	1804^{d}
	T_1	114.5^{b}	5.73^{c}	91.3^{c}	68^{b}	171^{c}	5.38^{c}	4.28^{c}	2073^{b}
	T_2	115^{a}	5.78^{b}	92.4^{b}	69^{a}	176^{b}	5.36^{b}	4.33^{b}	2060^{c}
	T_1+T_2	113^{d}	5.85^{a}	94.0^{a}	66^{c}	188^{a}	5.43^{a}	4.96^{a}	2450^{a}
**I_2**	T_0	113^{b}	5.66^{d}	89.0^{d}	70^{a}	164^{d}	5.6^{a}	4.0^{d}	1800^{d}
	T_1	114^{a}	5.81^{b}	90.0^{c}	67^{c}	170^{b}	5.20^{b}	4.15^{c}	2096^{c}
	T_2	114^{a}	5.80^{c}	90.5^{b}	68^{b}	169^{c}	5.16^{d}	4.19^{b}	2118^{b}
	T_1+T_2	113^{b}	5.83^{a}	91.0^{a}	65^{d}	178^{a}	5.29^{c}	4.23^{a}	2340^{a}
**I_3**	T_0	112^{b}	5.58^{d}	86.4^{d}	70^{a}	165^{d}	4.94^{d}	3.90^{d}	1650^{d}
	T_1	112^{b}	5.78^{c}	89.5^{c}	65^{c}	169^{c}	5.14^{c}	4.18^{b}	1943^{b}
	T_2	113^{a}	5.80^{b}	90.0^{b}	67^{b}	172^{b}	5.19^{b}	4.09^{c}	1940^{c}
	T_1+T_2	112^{b}	5.82^{a}	93.0^{a}	64^{d}	178^{a}	5.27^{a}	4.26^{a}	2353^{a}
**I_4**	T_0	113^{a}	5.52^{d}	85.8^{d}	69^{a}	150^{d}	3.89^{d}	3.67^{d}	1538^{d}
	T_1	112^{b}	5.68^{c}	88.4^{b}	67^{c}	168^{b}	5.16^{b}	4.13^{c}	1923^{c}
	T_2	113^{a}	5.70^{b}	87.0^{c}	68^{b}	163^{c}	5.14^{c}	4.15^{b}	1975^{b}
	T_1+T_2	111^{c}	5.79^{a}	92.3^{a}	62^{d}	176^{a}	5.32^{a}	4.26^{a}	2318^{a}
**F_{test}**	I × F	1.23*	2.65**	0.023ns	7.75*	68.16**	1.27**	0.53*	30.64**

Note: $p < 0.05$, **=0.01,***= 0.00, I_1= 70mm evaporation, I_2= 100mm evaporation, I_3= 120mm evaporation, I_4= 150mm evaporation, F_0= No fertilizer/PGRs, F_1= Chemical fertilizers, F_2= PGRs, F_3= Chemical fertlizers and PGRs

Table 4: Variation in Physiological attributes of Canola with chemical fertilizers and Plant growth regulators under different water regimes

Irrigation	Treatments	Chl a	Chl b	Total Chlorophyll	LWC	LT	Stomatal Conductance	Grain Yield
		(Ng/g DW)			(%)	(^{0}C)	(mmol m-^{2} s^{-1})	(gr/m)
**I_1**	T_0	1.43^{de}	0.834^{abc}	2.264^{d}	80.0^{ab}	21.8^{ef}	142.3^{a}	188^{g}
	T_1	2.18^{a}	0.87^{a}	3.05^{a}	83.0^{a}	19.7^{fg}	146.2^{a}	264^{a}
	T_2	1.63^{d}	0.84^{ab}	2.47^{c}	82.0^{a}	21.6^{fg}	144. 1^{a}	220^{d}
	T_1+T_2	1.95^{b}	0.85^{ab}	2.8^{b}	81.9^{a}	19.8^{fg}	146.42^{a}	249^{b}
**I_2**	T_0	1.28^{fgh}	0.78^{def}	2.06^{d}	80.82^{ab}	25.6^{f}	140.73^{a}	176.4^{h}
	T_1	1.88^{bc}	0.82^{bcd}	2.7^{b}	81.80^{a}	22.3^{ef}	145.12^{a}	232.42^{c}
	T_2	1.48^{def}	o.8^{cde}	2.28^{d}	81.17^{a}	23.5^{ef}	144.95^{a}	207.24^{e}
	T_1+T_2	1.72^{cd}	0.8^{cde}	2.52^{c}	82.0^{a}	21.8^{fg}	143.91^{a}	221.18^{bc}
**I_3**	T_0	0.73^{ij}	0.72^{h}	1.45^{f}	74.17^{de}	30.6^{b}	101.0^{c}d	131.06^{k}
	T_1	1.18^{gh}	0.75^{fgh}	1.93^{e}	76.16^{cd}	28.5^{cd}	107.9^{bcd}	165.97^{i}
	T_2	1.18^{gh}	0.75^{gh}	1.93^{e}	78.18^{bc}	27.6^{de}	117.26^{b}	172.03^{h}
	T_1+T_2	1.39^{ef}	0.76^{efg}	2.15^{h}	78.84^{bc}	25.91^{f}	119.47^{b}	198.21^{f}
**I_4**	T_0	0.47^{k}	0.68^{j}	1.15^{h}	62.49^{f}	35.0^{a}	70.94^{e}	170.52^{h}
	T_1	0.66^{j}	0.71^{ij}	1.37^{g}	65.18^{f}	33.4^{ab}	78.61^{e}	173.23^{h}
	T_2	0.85^{i}	0.70^{ij}	1.55^{f}	71.80^{e}	28.92^{c}	97.67^{d}	133.05^{k}
	T_1+T_2	1.13^{h}	0.72^{hi}	1.85^{e}	73.6^{de}	27.9^{cd}	110.4^{bc}	156.84^{j}
**F_{test}**	I × F	0.134**	0.00032**		0.139**	21.99**	143.4**	1803.26**

Note: $p < 0.05$, **=0.01,***= 0.00, I_1= 70mm evaporation, I_2= 100mm evaporation, I_3= 120mm evaporation, I_4= 150mm evaporation, F_0= No fertilizer/PGRs, F_1= Chemical fertilizers, F_2= PGRs, F_3= Chemical fertlizers and PGRs

Table 5: Variation in quality traits of Canola with chemical fertilizers and Plant growth regulators under different water regimes

Irrigation	Treatments	Oil content (%)	Oil yield (kg ha^{1})	Protein (%)	Glucosionalate (µmol/g)	Moisture (%)	Erucic acid (%)
**I_1**	T_0	38.0^d	238.0^d	20.77^d	26.2^a	5.23^d	4.44^a
	T_1	38.5^c	324.5^c	24.82^c	22.0^c	5.72^c	4.20^b
	T_2	39.0^b	328.9^b	24.97^b	18.7^d	5.96^b	3.74^c
	T_1+T_2	42.8^a	468.0^a	25.07^a	14.6^f	6.77^a	3.22^d
**I_2**	T_0	37.0^d	234.6^d	20.0^d	24.0^b	5.0^d	5.21^a
	T_1	37.8^c	340.0^c	24.82^c	20.0^c	5.36^b	4.37^b
	T_2	38.2^b	355.0^b	24.79^b	19.0^d	5.30^c	3.98^c
	T_1+T_2	42.5^a	437.8^a	25.0^a	16.0^e	5.98^a	3.41^d
**I_3**	T_0	36.0^d	229.0^d	18.5^d	23.5^b	4.93^c	4.98^a
	T_1	37.3^c	324.5^c	23.0^c	17.0^e	5.11^c	3.75^c
	T_2	37.9^b	329.38^b	23.5^b	18.2^d	5.19^b	3.82^b
	T_1+T_2	41.6^a	398.0^a	24.8^a	14.0^f	5.75^a	3.45^d
**I_4**	T_0	34.8^d	220.0^d	18.0^d	20.0^c	4.81^d	4.99^a
	T_1	36.0^c	378.3^c	23.0^b	18.0^d	5.25^c	3.68^c
	T_2	36.5^b	382.0^b	22.5^c	16.5^e	5.29^b	3.71^b
	T_1+T_2	41.0^a	437.2^a	24.2^a	11.6^g	5.78^a	3.50^d
**F_{test}**	I × F	13.68**	6.34*	3.36**	4.28*	0.985**	12.78**

Note: $p < 0.05$, **=0.01,***= 0.00, I_1= 70mm evaporation, I_2= 100mm evaporation, I_3= 120mm evaporation, I_4= 150mm evaporation, F_0= No fertilizer/PGRs, F_1= Chemical fertilizers, F_2= PGRs, F_3= Chemical fertlizers and PGRs

Table 6: Variation in Nitrogen (N), Phosphorus (P) and Potassium (K) of Canola with chemical fertilizers and Plant growth regulators under different water regimes

Irrigation	Treatments	N (%)	P (%)	K (%)	N uptake (kg ha^{1})	P uptake (kg ha^{1})	K uptake (kg ha^{1})
**I_1**	T_0	2.70^{d}	0.18^{d}	1.21^{d}	22.81^{d}	17.43^{d}	16.34^{d}
	T_1	3.72^{b}	0.21^{b}	1.68^{b}	38.45^{b}	21.09^{b}	18.23^{b}
	T_2	3.59^{c}	0.20^{c}	1.55^{c}	34.23^{c}	19.94^{c}	17.78^{c}
	T_1+T_2	3.88^{a}	0.24^{a}	1.88^{a}	51.84^{a}	23.29^{a}	22.67^{a}
**I_2**	T_0	2.69^{d}	0.20^{c}	1.20^{d}	22.86^{d}	18.43^{d}	16.0^{d}
	T_1	3.35^{c}	0.20^{c}	1.55^{b}	38.98^{b}	20.45^{b}	18.45^{b}
	T_2	3.54^{b}	0.21^{b}	1.43^{c}	29.74^{c}	19.90^{c}	18.21^{c}
	T_1+T_2	3.75^{a}	0.22^{a}	1.78^{a}	50.95^{a}	22.45^{a}	21.45^{a}
**I_3**	T_0	2.71^{d}	0.19^{c}	1.16^{d}	20.09^{d}	17.09^{d}	14.76^{d}
	T_1	3.15^{c}	0.22^{a}	1.61^{b}	30.56^{b}	19.90^{b}	17.85^{c}
	T_2	3.19^{b}	0.21^{b}	1.34^{c}	26.64^{c}	18.64^{c}	17.93^{b}
	T_1+T_2	3.84^{a}	0.21^{b}	1.72^{a}	49.80^{a}	21.98^{a}	20.06^{a}
**I_4**	T_0	2.55^{d}	0.17^{c}	1.03^{d}	17.65^{d}	15.43^{d}	13.49^{d}
	T_1	3.38^{c}	0.21^{b}	1.45^{c}	30.32^{c}	18.45^{c}	17.83^{c}
	T_2	3.45^{b}	0.21^{b}	1.54^{b}	33.90^{b}	19.05^{b}	16.98^{b}
	T_1+T_2	3.78^{a}	0.24^{a}	1.69^{a}	48.73^{a}	20.97^{a}	19.56^{a}
**F_{test}**	I × F	3.05**	0.168***	0.98**	16.24*	9.34***	3.44**

Note: $p < 0.05$, **=0.01,***= 0.00, I_1= 70mm evaporation, I_2= 100mm evaporation, I_3= 120mm evaporation, I_4= 150mm evaporation, F_0= No fertilizer/PGRs, F_1= Chemical fertilizers, F_2= PGRs, F_3= Chemical fertlizers and PGRs

Table 7: Variation in Biochemical attributes of Canola with chemical fertilizers and Plant growth regulators under different water regimes

Irrigation	Treatments MSI	PPO	CAT	POX	SOD		MDA	Soluble sugar	Proline
				(U g^{-1} FW)			(mmol g^{-1} FW)	(mg /g DW)	(mmol g^{-1} FW)
**I_1**	T_0	0.47^{f}	0.21^{h}	0.15^{f}	0.26^{g}	2.4^{j}	31.4^{ijk}	15.4^{ij}	86.71^{ab}
	T_1	0.54^{f}	0.23^{h}	0.17^{f}	0.31^{fg}	2.29^{j}	30.0^{k}	16^{ij}	87.92^{a}
	T_2	0.52^{f}	0.26^{h}	0.16^{f}	0.33^{fg}	2.28^{j}	31.0^{ijk}	15.03^{ij}	87.81^{ab}
	T_1+T_2	o.57^{f}	0.24^{h}	0.18^{f}	0.32^{fg}	2.27^{j}	30.2^{jk}	15.07^{ij}	87.45^{ab}
**I_2**	T_0	0.79^{f}	0.6^{gh}	o.36^{ef}	0.59^{efg}	3.5^{gh}	32.8^{ij}	16.36^{hi}	85.0^{abc}
	T_1	0.80^{f}	0.64^{g}	0.88^{e}	0.63^{efg}	3.07^{hi}	31.63^{ijk}	16.1^{ij}	87. 25^{ab}
	T_2	1.02^{f}	0.65^{g}	0.95^{e}	0.74^{e}	2.66^{ij}	32.24^{ijk}	15.8^{ij}	88.0^{a}
	T_1+T_2	1.08^{f}	1.38^{fg}	1.57^{d}	o.79^{e}	2.44^{j}	32.87^{i}	15.57^{ij}	88.07^{a}
**I_3**	T_0	1.88^{e}	2.0^{f}	1.67^{d}	1.28^{d}	5.9^{bc}	38.56^{h}	22.8^{c}	75.62^{e}
	T_1	2.03^{e}	2.08^{f}	1.86^{d}	1.31^{d}	5.29^{ed}	43.6^{f}	21.9^{bc}	78.45^{d}
	T_2	2.82^{cd}	3.15^{d}	2.69^{c}	2.10^{c}	4.34^{f}	45.64^{ef}	18.14^{fg}	80.05^{cd}
	T_1+T_2	3.38^{bc}	3.56^{c}	2.87^{c}	2.18^{c}	3.72^{g}	49.07^{d}	17.34^{gh}	81.98^{bc}
**I_4**	T_0	1.9^{e}	2.75^{e}	2.84^{c}	1.96^{c}	7.30^{a}	41.0^{g}	32.68^{a}	64.09^{g}
	T_1	2.15^{e}	2.85^{e}	3.12^{bc}	1.90^{c}	6.34^{b}	43.52^{f}	27.44^{b}	65.32^{g}
	T_2	3.63^{b}	4.07^{b}	3.85^{b}	2.95^{b}	5.57^{cd}	63.36^{b}	22.08^{cd}	70.29^{f}
	T_1+T_2	4.19^{a}	4.39^{a}	4.44^{a}	3.44^{a}	5.72^{cd}	67.62^{a}	21.4^{de}	74.49^{e}
**F_{test}**	I × F	0.612**	0.49**	0.317**	0.34**	0.509**	99.01**	14.49**	14.22**

Note: $p < 0.05$, **=0.01,***= 0.00, I_1= 70mm evaporation, I_2= 100mm evaporation, I_3= 120mm evaporation, I_4= 150mm evaporation, F_0= No fertilizer/PGRs, F_1= Chemical fertilizers, F_2= PGRs, F_3= Chemical fertlizers and PGRs

Click here to access/download
Supporting Information
Minimal data set_Plosone (1).xlsx

Physiological and Biochemical Exploration of Canola for Overcoming Yield Limitation by Integrated Fertilizer management under different water regimes

Muhammad Mahran Aslam[a], Fozia Farhat[b], Mohammad Aquil Siddiqui[a*], Shafquat Yasmeen[a], Muhammad Tahir Khan[a], Mahboob Ali Sial[a] and Imtiaz Ahmad Khan[a]

[a]Nuclear Institute of Agriculture (NIA), Tando Jam, 70060, Sindh, Pakistan

[b]Department of Botany, Government College Women University, Faisalabad, Pakistan

[*]Corresponding author: Mohammad Aquil Siddiqui[a]

Corresponding author email: Siddiqui_aquil@yahoo.com

ABSTRACT

Environmental stressors may modify the nutritional profile and economic value of crops. Chemical fertilizers and phyto-hormones are major natural sources which can enhance the canola production under stressful conditions. Physio-biochemical responses of canola altered remarkably with the use of nitrogen/phosphorus/potassium (N/P/K) fertilizers and plant growth regulators (PGRs) under drought stress. The key interest of current study was to evaluate nutritional quality and physio-biochemical modulation in canola (*Brassica napus* L.) from early growth to seed stage with chemical fertilizers (NPK) and plant growth regulators (PGRs) in different water regimes. To monitor responses in canola (Surhan-2012), two separate field experiments were conducted as spilt plot under randomized complete block design (RCBD) with four treatments (No fertilizer, Chemical fertilizers, PGRs and the combination of chemical fertilizers and PGRs) under different irrigations regimes (60, 100, 120, 150 mm evaporations). Water stress enhanced activity of peroxidase (POD), catalase (CAT), superoxide dismutase (SOD), polyphenol oxidase (PPO), soluble sugar, leaf temperature, malondialdehyde (MDA) and proline contents while substantially reduced leaf water contents, stomatal conductance, chlorophyll contents, membrane stability index and grain yield of canola were reduced. However, The the combined application of fertilizers and plant growth regulator (PGR) further increased the enzymatic antioxidant poolactivities, soluble sugars, along with recovery of leaf water contents, chlorophyll contents, stomatal conductance and membrane stability index but decreased the proline contents and leaf temperature at different rate of evaporation. There is positive interaction of applied elicitors to the water stress in canola except leaf area. The outcomes depicted that the combination of NPK with PGRs improved the various morpho-physiological as well as biochemical parameters and reduced the pressure of chemical fertilizers cost about 60%. It had also reduced the deleterious effect of water limitation on the physiology and grain yield and oil contents of canola in field experiments.

Keywords: antioxidants, elicitors, mitigation strategies, photosynthesis, water shortage, yield

1. Introduction

Among oilseed crops, canola (*Brassica napus* L.) withhold a special rank worldwide as the second highly significant oil yielding crop [1](FAO, 2015) with over ~40–50% oil and 40% protein (in rapeseed meal) contents after soybean [2]. With constant efforts of scientists, the production of canola had reached a milestone by enhancing its yield and area upto 68.9 million metric tons

and 33.7 million hectares, respectively [3].The leaves of this plant are imperative source of animal feed due to encompassing a balanced ratio of protein and fibrous [4]. Oil extracted from oilseed rape contains a very high concentration of unsaturated fatty acids (such as oleic acid and linoleic acid (C18:3, ~10% v/v)) making it a fatty acid-rich diet source [5]. Pakistan facing the low yield of canola due to the inefficient management of macro and micronutrient. Globally, it is emerging oil seed crop among the other oil seeds due to low erucic acid and glucosinolates in oil and seed cake respectively [3].Thus, the nutrient management strategy is quite useful source to optimize the canola yield in the arid and semi-arid region of the country [4]. The drastic change in climate and rapid increase of world population is the threat of sustainable food security. These climate change favors the drought and heat stress that limit the crop productivity and effecting future food security [2]. The rain pattern has also been transformed due to climate shift which boosted the drought stress all over the world which decline the crop productivity by effecting physiology, plant growth and reproduction of plant severely[5].

Field Code Changed

Like many other major temperate field crops, canola is particularly susceptible to environmental stress alone and in combinations of multiple stresses, such as heat and drought (Elferjani & Soolanayakanahally, 2018) (Champolivier & Merrien, 1996)(Aksouh-Harradj et al., 2006). Reduced biomass and chlorophyll contents due to deterioration of chloroplast structure along with reduced production of seed oil and protein contents are usual symptoms of water stress in *B. napus* (Ahmar et al., 2019). The plant growth and development mainly restricted by the drought stress via declining turgor pressure of plant cells which caused hindrance in the biochemical and physiological intriguing mechanisms of plants [6]. The drought stress also drastically effect the photosynthesis of the plants [7]. The water deficit condition cause the reduced concentration of intracellular CO_2, chlorophyll destruction, photochemical system disorder and stomatal closure [8]. The plant showed physiological response in the form of reactive oxygen species (ROS) production under drought stress. The excess amount of ROS can damage the cell membrane by elevating the lipid peroxidation[9,10]. The plants mitigate the ROS harmful effect by the activities of enzymatic and non- enzymatic antioxidants[11]. Superoxide dismutase (SOD), peroxidase (POX), catalase (CAT) and polyphenol peroxidase (PPO) are the antioxidant enzymes which diminished the ROS concentration during drought stress [9,12]. The accumulation of osmolytes, proline, soluble sugar, soluble proteins are the non-enzymatic reaction that caused osmatic regulation under water deficient condition stress [13]. The antioxidant activities elevated

under stress condition in ajowan and canola plant against ROS [14]. The plants showed increase level of proline, antioxidants including POX. PPO and SOD but reduced MDA level under stress condition [15,16]. ~~The performance of plant declined due to water deficit stress by reducing Chl a, Chl b, Membrane stability index, chlorophyll content index and increasing leaf temperature [9].~~

~~Balance application of fertilizer influence the crop yield, quality with soil sustainability [17] .~~ Nitrogen play a pivotal role in plant tissue growth and development, being an integral part of protein, chlorophyll, nucleotides, protein, amino acid which directly influence the quality and quantity of crop production [18]. The adequate nitrogen supply is important attribute to boost up the canola productivity [19]. Thus, any fluctuation in the soil profile, texture, and moisture content at various critical stage of growth and development may sway the nitrogen use efficiency in canola. Canola crop is very responsive to fertilizer application especially nitrogen, phosphorus and potassium which significantly effects the growth and yield per ha [20]. It also stimulate the leaf area (LA) development after flowering in canola [21,22].

The management of soil fertility is limiting factor for the sustainable agriculture production [20]. This challenges of soil fertility can be overcome by the optimal application of fertilizers and plant growth regulators [23]. The chemical and bio- fertilizer are very effective in improving the micro and macro nutrient via organic compound degradation and nitrogen fixation [24]. This lead to improve nutrient uptake and reduce impact of drought in crops. This would increase the activities of PPO, CAT and POX which ultimately improve the grain yield under drought stress in response to fertilizer and PGRs [9]. The combined application of chemical fertilizers, bio fertilizers and PGRs enhanced the accumulation of proline, sugar contents and chlorophyll contents [25]. The fertilizers along with plant growth regulators elevated the stomatal conductance, water contents and total chlorophyll content under water limiting conditions [26]. These combined application of nutrients improve the soil fertility under water stress condition [27]. Although genetic manipulation has a promising effects to mitigate this problem and also stated mid- and long-term results, but current world situations required some immediate response methods for the the growing demands of food and feed. In spite of numerous scientific efforts, information related to biochemical, physiological and yield dynamics with respect to combined application of chemical fertilizers and PGRs is very limited for canola production. Thus, this manuscript

Formatted: Pattern: Clear (White)

comprised to gauge the morphological, physiological and biochemical rejoinders of canola to assimilate fertilizers management and PGRs with limited supply of water.

2. Materials and Methods

2.1. Experimental design and treatments

The two-year (2018-19 & 2019-2020) field experiment was conducted at experimental field of Nuclear Institute of Agriculture (NIA), TandoJam, Sindh to investigate the variability in physio-biochemical parameters and yield of canola (Surhan-2012) in reaction to fertilization under excessive rate of water evaporation. The experiment was performed in split plots as random complete block design (RCBD) in triplicate with four irrigation levels.

**Irrigation Levels**	**Treatment with Elicitors**
1_0= 60mm evaporation	T_0 = No treatment
	T_1= Chemical Fertilizers [N (90 kg/ha), P (45 kg/ha) and K (45 kg/ha)]
	T_2 = PGRs [IAA (15g/ha) and GA_3 (15g/ha)]
	$T_3 = T_1 + T_2$
1_1= 100mm evaporation	T_0 = No treatment
	T_1 = Chemical Fertilizers [N (90 kg/ha), P (45 kg/ha) and K (45 kg/ha)]
	T_2 = PGRs [IAA (15g/ha) and GA_3 (15g/ha)]
	$T_3 = T_1 + T_2$
1_2= 120mm evaporation	T_0 = No treatment
	T_1 = Chemical Fertilizers [N (90 kg/ha), P (45 kg/ha) and K (45 kg/ha)]
	T_2 = PGRs [IAA (15g/ha) and GA_3 (15g/ha)]
	$T_3 = T_1 + T_2$
1_3= 150mm evaporation	T_0 = No treatment
	T_1 = Chemical Fertilizers [N (90 kg/ha), P (45 kg/ha) and K (45 kg/ha)]
	T_2 = PGRs [IAA (15g/ha) and GA_3 (15g/ha)]
	$T_3 = T_1 + T_2$

Weeding operations was done ~~continued~~ frequently ~~when needed~~ during ~~crop~~ growth and development of surhan-2012 plants in both years.

2.2. Determination of Agronomic and yield related parameters

At vegetative stage, height of ten randomly selected canola plants of each treatment were measured with measuring tape (cm) from ground to the tip of flag leaf and counted number of branches per plant while number of siliqua plant^{-1}, siliqua length (cm) and number of seeds siliqua^{-1} were counted after harvesting. Similarly, recorded the data related to days to flower when 50% plants had 50% flowers and days to maturity at discoloration stage. Grains of canola plants were dried in the sun and recorded 1000 grain weight by using digital balance (Model- Explorer OHAUS). Biological yield was determined after harvesting from 4 rows and calculated as kg ha^{-1}.

2.3. Determination of Quality traits

Oil content were extracted with petroleum ether using soxhlet apparatus. All the dried seed samples were coarsely ground and packed carefully into the thimble for oil extraction. The extraction was performed continuously for three cycles (90-120 min.) and oil productivity was drawn through standard formula. Two significant fatty acids, erucic acid (%) and glusionlate (µmol g^{-1}) were detected through High Performance Liquid Chromatography (HLPC). Oil contents were analysed using Gradient HPLC (Shimadzu, Japan) having LC-10AT, SCTL 10A system controller, SPD-10AR UV-VIS detector at 280 nm with C18 stationary column (Shim-Pack CLC-ODS). Elution was done for 60 min with a flow rate of 1ml/min in a gradient system of two mobile phases A (H_2O_2: AA-94:6, pH 2.27), B (ACN100%)[28]. Moisture contents (%) was determined by the weight of water in a seed.

2.4. Determination of NPK uptake by the Canola GrainPlant nutrient content and uptake (grain)

For determination of phosphorus (P) and potassium (K) contents, seed samples of each treatment were dried at 70°C for 48h. Dried and powdered grain sample (0.5g) was digested with 20mL concentrated nitric acid (HNO_3) by adopting method of Rathje and Jackson [29]. The samples were placed for 3 hours at room temperature. After 3 hours, samples were laidput on the digestion block at 250° C until the solution became tinted yellow in appearance. The digested solution was diluted with 50mL of distilled water and filtered with whatman No. 42 filter paper. The phosphorus (P) contents from the digested plant samples was determined by recording optical density at 430 nm with spectrophotometer (Model-Spectronic-21) by Primson et al. [31]. The potassium (K) content in gains was resolute by flame photometer (Model-Flame photometer-400) according to the method suggested by Tammam [32]. Nitrogen content was examined by kjeldahl apparatus (Jones, 1991). Following formulas were applied to determined NPK uptake in grains of canola as kg ha^{-1}.

Formatted: Indent: First line: 0"

N uptake (kg ha^{-1}) in grain: N (%) x grain yield (kg ha^{-1}) /100

P uptake (kg ha^{-1}) in grain: P (%) x grain yield (kg ha^{-1}) /100

K uptake (kg ha^{-1}) in grain: K (%) x grain yield (kg ha^{-1}) /100

2.5. Determination of Physiological and Biochemical parameters

2.5.1. Chlorophyll Contents

Fresh leaf samples were collected from each treatment and subjected to grinding with 80% acetone. Semi-liquid extract was filtered and centrifuged at 10000rpm for 5minutes [33]. The supernatant was then subjected to spectrophotometer (Model Analytikjena Spekol 1500 Germany)

2.5.2. Leaf water content

Leaf water content was measured by harvesting three leaves per plant from every plot after 45 days of sowing (DAS). Fresh leaf sample- was weighed in gram (g) as fresh weight (FW) and let them dry at high temperature (80^{0}C) and reweighed as dry weight (DW). Leaf water content (LWC) was calculated by following formula.

$$LWC = [(FW – DW) / (FW)]$$

2.5.3. Leaf temperature

The leaf temperature (LT) was measured at flowering stage with the help of infrared thermometer (TES- 1327). The leave temperature (^{0}C) was measured by randomly selecting 3 plants of every treatment and replicate. Later, the mean LT was carefully recorded.

2.5.4. Stomatal conductance (g_s)

Portable photosystem (Porometer AP4, Delta-T Devices Ltd., Cambridge, U.K.) was used to measure the stomatal conductance. This data was carefully recorded 60 days after sowing (DAS).

2.5.5. Membrane stability index (MSI)

The previously reported method of Ghassemi-Golezani et al. [34] with slight modification was used to calculate membrane stability index. ~About 0.1 g leaf samples was mixed with double distilled water (10 ml) in falcon tube and incubated at 40^{0}C for 30 min and electrical conductivity

was measured (EC_1). Thereafter, that conductivity of these sample were assessed after placing water bath at 100^{0}C for 10 min (EC_2). The MSI was measured by the following formulas:

$$\text{Membrane Stability Index (MSI)} = (EC_1 / EC_2) \times 100$$

2.6. Determination of osmolytes

The total soluble sugar content was estimated from the dried leaves of all the replicates of respective canola treatments in canola leaves[35]. The standard calibration curve of pure glucose was used to determine total soluble sugars of leaves and expressed as mg/g DW. To determine proline contents in canola, leaf sample was thoroughly grinded in 3% sulfosalicylic acid. The extracted sample was filitered,poured and mixed with glacial acetic acid and ninhydrin in a test tube with a ratio of 1:1:1. Thisese mixture was heated at 100^{0}C for 60 min in a Bain Marie oven. Then reaction mixture was cooled at room temperature and the toluene used for the extraction of mixture, vortexed for 30sec. The absorbance of the upper organic phase was recorded at 520 nm. Calibration curve of pure proline was used to compare the proline content of canola leaves and expressed as mg/g FW [36].

2.7. Determination of Antioxidants

Young leaves were collected from each treatment at 60 DAS and assayed the activity of polyphenol oxidase (PPO) by Kumar and Khan (1982) method [37]. The reaction mixture contains 0.1 M phosphate buffer, 1 ml catechol and 5 ml enzyme extract. The reaction mixture was incubated at 25^{0}C for 5 min, later, the reaction was terminated by dissolving 1 ml of 3 ml NH_2SO_4. The PPO activity was determined in the form of absorbance of resultant purpurogallin at 495 nm and expressed as Umg^{-1} (U= change in 0.1 absorbance min^{-1}, mg^{-1} protein. The CAT activity was determined with an interval of 20 seconds for 2 minutes at 240 nm (Ug^{-1} FW) according to the devised method of Singh and Sharma[38]. The POX activity was observed with an interval 0f 30 sec for 2 minutes at 470 nm due to guaiacol oxidation. The activity was determined from reaction solution consisted 1 ml of 1% guaiacol, 0.3 ml of enzyme extract, 2.5 ml of 50 mM potassium buffer (pH= 7.0) and 1 ml of 1% H_2O_2 for 2 min in reaction mix [39]. The SOD activity was assessed by the estimation of volume of enzyme effected as 50% inhibition of nitro blue tetrazolium [39].

2.8. Determination of Lipid peroxidation

Malondialdehyde content (mmol g^{-1} FW) from canola leaves was determined 60 DAS [40] to estimate rate of lipid peroxidation [40]. ˜0.5 g of fresh leaves was homogenized in 5% trichloroacetic acid (5 ml), heated at 25 ^{0}C for 10 minutes and centrifuged at 1800 g. The 2-thiobarbituric acid (TBA) was added in supernatant, placed at 98^{0}C for 10 min and cooled at room temperature. Finally, recorded the absorbance at 532 nm with spectrophotometer.

2.9. Statistical analysis

All the experimental data was recorded and subjected to analysis of variance (ANOVA) with linear models of statistics to observe statistical significant/non-significant differences among different traits of *Brassica napus* through computer program, Student Edition of Statistix (SWX), Version 8.1 (Analytical Software, 2005). Moreover, least significant difference (LSD) test was applied to verify the level of significance (5%) among different combination means[41].

3. Results

The results of canola presented in this manuscript was recorded for two consecutive years i.e. 2018-2019 and 2019-2020. The mean of all attributes have been tabulated and described in the result section (Table 1-7). Weather data was carefully monitored from October to April for both growing years (2018-19/2019-20). The maximum and minimum temperature decreases from October to March and then increases marginally in April for both growing seasons (Table 1). The soil of experimental area was analyzed and found 2% organic matter (OM) along with sodium (0.08), potassium (0.11), magnesium (3.2), sulpher (16.90), zinc (1.40), boron (0.34), phosphorus (10.83) and calcium (2.5) as major nutrients. The soil mineral contents were calculated as µg/g soil (Table 2).

3.1. Agronomic and yield performance of canola with fertilizers and PGRs under drought

~~Weather data from October, 2018 to April, 2020 was recorded very carefully (Table 1) and soil profile was also checked by using dry sample of soil from the experimental area (Table 2).~~

The mean data of two consecutive years of agronomic as well as yield attributes (plant height, days to flower, number of branches per plant, number of seed per plant, biological yield per plot, 1000 seed weight and seed yield) presented a significant ($p < 0.01$) interaction of irrigation to that of NPK and PGR (Table 3). The plant growth was affected by the severe water stress (I_4), when no elicitor was provided to the canola seedlings. The combination of ~~chemical fertilizers~~NPK (T_1)

and PGRshormone (T_2) enhanced the agronomic performance under severe water deficit (I_4=150mm evaporation) condition by improving number of seeds/plant (1.76), biological yield/plant (5.32Kg), 1000 seed weight (4.32g) and seed yield/hectare (2318Kg/ha) (Table 3**).** The low water supply during critical growth stage reduced the yield of canola (Table 3). Moreover, PGR showed non-significant difference among all levels of evaporation for plant height but significantly vary for other agronomic and yield traits. The fertilizer applications significant enhanced the biological seed yield/plant (5.43Kg) of canola and the highest yield (2450Kg/ha) was recorded with NPK (T_1) under normal irrigation (I_0). The T_1 and T_2 treatment presented a non-significant difference under normal irrigation condition. However, the T_3 treatment significantly increased the grain yield of canola under mild (I_3) and severe water stress (I_4), compared to other treatments. The maximum grain yield was recorded with $T_{1,}$ compared to T_1 under normal irrigation and declined with the severity of water stress. Moreover, data displayed a strong and significant interaction between different rate of evaporation (I) and applied elicitors (T) in all studied features of canola except leaf area (Table 3).

3.2. Physiological performance of canola with fertilizers and PGRs under drought

The mean data of two-year field experiment of canola revealed a highly significant ($p < 0.01$) response of NPK and PGR application to chlorophyll contents under data related to *Chl a, Chl b* and *total chlorophyll* contents presented a significant ($p < 0.01$) effect of chemical fertilizer (T_1) under water deficit conditionstress (Table 4). The chlorophyll contents decline significantly in canola with increasing rate of evaporation (I_0-I_4). However, the exogenous application of NPK treatment (T_1) significantly enhanced *Chl a*, *Chl b* and total chlorophyll contents under control but combination of NPK and PGRs (T_3) progressively recovered the chlorophyll contents from least to severe water stressall level of irrigation except (I2-I_4I). The *Chl b* contents decreased significantly with progression of water stress, even and applied treatmentsfortification of NPK and PGRs also failed to mitigate adverse effects of over evaporation. The T_1 and T_2 treatment of chemicals to mitigate water stress showed non-significant difference for all studied features of photosynthesis. A highly significant relation was observed between different irrigation regimes (I) and treatments with elicitors (T) for chlorophyll contents and also showed a recovery mechanism by promoting photosynthetic activity (Table 4).

Formatted: Not Superscript/ Subscript

The average of two season's data for water contents and temperature of canola leaves showed a significant ($p < 0.01$) interaction of irrigation and elicitorsfertilizer (Table 4). Both these tratisfeatures behave worked antagonistically to each other, with the decrease in leaf water content (LWC), leaf temperature (LT) increased during severe drought (I_4) condition. The irrigation regimes I_1 (Control) and I_2 (MRE) showed non-significant difference on water contents and leaf temperature and same effects were observed with chemical fertilizer (T_1) under mild (I_1) and moderate (I_2) rate of evaporation. However, the T_3 treatment increased the LWC by reducing LT of canola under mild (I_0) and severe stressful (I_4) condition (Table 4).

The NPK application significantly ($p < 0.01$) influenced the stomatal conductance of canola plant withunder different rate of evaporation (Table 4). The water stress reduced the stomatal conductance under severe rate of evaporation (I_4) butand showed exhibited non--significant change under the mild (I_1) and moderate (I_2) rate of irrigation evaporationstress. The combined effect of PGRs (T_2) and chemical fertilizers (T_{32}) significantly enhanced the stomatal conductance under normal (I_0) and mild (I_1) water stress. A drastic reduction of stomatal conductance was recorded with increasing water stress. A significant interaction of stomatal conductance (143.4**) was recorded for evaporation and applied treatments (Table 4).

Formatted: Subscript

3.3. Qualitative performance of canola with fertilizers and PGRs under drought

The mean of two years statistical data depicted that the interactive effect of irrigation and fertilizers significantly influenced the quality related traits (oil contents, oil yield, protein, glucosinolates, and moisture and erucic acid contents) of canola (Table 5). These features declined with the progression of water deficit condition. The experimental results defined the positive influence of chemical and PGRs (T) onThe maximum oil yield, oil contents, oil yield, protein and moisture contents were recorded with T_3 under normal (I_0) and severe water deficit (I_4) condition, while reduced the glucosinolates and erucic acid declined the same treatmentcontents (Table 5). It is summarized that PGRs (T_{32} tretament) is a good rehabilitation strategytechnique to improve quality of canola followed by chemical fertilizers ($T_1$$T_1$ and T_2 to address water scarcity issues of canola.

3.4. Nitrogen, Phosphorus and Potassium (NPK) contents and their Uptake in canola

The mean data of two consecutive years(2018-19/2019-20) showed that combined effect of fertilizer and PGRs (T_3~~$_2$~~) significantly ($p < 0.01$) influence the NPK contents and their uptake in canola plant. Provision of chemical fertilizer (T_1) individually or in combination with PGRs (T_3) improved the NPK contents as well as their uptake under mild (I_0) irrigation stress (Table 6).

3.5. Biochemical performance of canola with fertilizers and PGRs under drought

The results obtained from two-year trails illustrated that the osmolytes considerably ($p < 0.01$) influenced by the applied elicitors under different irrigation regimes. The level of proline and soluble sugar elevated in response to ~~decreasing water supply~~mild to severe rate of evaporation ~~to~~ canola plant as an innate response mechanism. Further, the NPK and PGRs (T_3) ~~application~~ showed a negative impact on the proline accumulation but positively enhanced soluble sugar contents under all level of irrigation (Table 7). The ~~maximum osmolyte accumulation~~proline and total soluble sugar contents increased and decreased respectively ~~was recorded~~ with the application of NPK (T_1) followed by PGRs (T_2) and combined treatment (T_3) under all levels of irrigation (Table 7).

The antioxidant enzymes and MDA activities significantly ($p < 0.01$) influenced by the irrigation (I) and elicitors ~~treatments~~ (T) in canola (Table 7). The canola plant enhanced the activities of various antioxidants and enzymes including SOD, CAT, PPO, POX and MDA contents in response to water stress. The canola behavior with fertilizers did not show significant effect during normal (I_1) and mild irrigation (I_2) stress except lipid peroxidation and POX activity. The T_1 treatment exhibited non-significant difference on enzyme activities but the MDA content influenced significantly under all irrigation levels (I_0-I_4). The treatment T_2 (PGRs) and T_3 (NPK and PGRs) elevated the activities SOD, PPO, POX and SOD but reduced the MDA contents as compared to T_0 under severe water deficit (I_4) condition. (Table 7).

The interaction of irrigation intervals (I) and elicitors (T) significantly affect the membrane stability index (MSI) of canola ($p < 0.01$). The water stress significantly reduced the membrane stability index. Only chemical fertilizer did not effect on the MSI while the combined fertilizers and plant growth regulators significantly improve the MSI under moderate and severe water stress. The chemical fertilizer only showed superiority under irrigation level of I_3 (Table 7).

4. Discussion

The findings of the current work highlighted~~s~~ the comparison of combined and individual application of PGR and NPK to enhance canola growth, nutritional quality and yield ~~under~~ for growing concerns of water scarcity. Particularly moderate to severe d~~D~~rought stress (I_3-I_4) imparts drastic effect on the ~~plant~~ canola growth by causing injuries~~y~~ at all growth stages. It influenced~~s~~ the various morphological (reduced leaf growth, leaf area, plant height, number of nodes per plant), physiological traits (chlorophyll content, leaf water contents, leaf temperature and stomatal conductance) at the onset of water scarcity (Table 3, 4). Reduction of plant height was recorded in canola under different irrigation regimes compared to exogenously applied PGRs and NPK (Table 3). Growth retardation due to excessive evaporation, can be related to disruption of photosynthetic machinery and decline in carbon reserves for relocation to growing parts of plant [42,43]. It seems that the decrease in plant height also interferes with leaf area. This reduction is particularly noticeable during post vegetative stage, flowering stage or abscission [43]. The grain yield reduced due to water stress as reported in pervious study [53]. Seed quality and 100 seed weight are commercial as well as economic traits, significantly compromised with ongoing scenario of water scarcity, so is in the present experiment (Table 3). Seed filling is particularly influenced by drought stress by modulating of various metabolic activities occurring in the leaves, such as synthesis and translocation of photoassimilates, essential substrates for biosynthesis of seed storage reserves, mineral nutrients and many more functional constituents (Sehgal et al., 2018). ~~Growth regulators and chemical fertilizers were significantly effective in mitigating the drastic effects of drought by maintaining the water efficiency of canola plants and augmenting the accretion of osmolytes. This may also favors the improvement of photosynthetic and gas exchange attributes .~~

In the current experiment, drought led to reduction in chlorophyll content, and this loss could be due to some devastating effects on photosynthetic apparatus (Table 4~~3~~). Stomatal conductance (g_s) was severely hampered when plants were exposed to severe~~a~~ ~~water deficit condition~~rate of evaporation (I_4). ~~This may have been resulted~~The resistance in stomatal conductance (g_s) may be correlated to ~~for~~ enhanced production of ABA under drought stress, which ~~that~~ leads to stomata closure. ~~This suggested that the~~ ABA signaling mechanism tries to prevent the loss of tissue turgor by closing the stomata [44,45]. The optimal use of chemical fertilizers and PGR appreciably enhanced stomatal conductance under drought stress (Table 4). Yan et al. [46] also r~~R~~eported ~~that~~ diffusional restrictions of CO_2 by stomata (52%), which directly caused a reduction of ~~could explain loss of~~ c~~C~~hlorophyll contents (31%) induced by drought. The

Formatted: Subscript

fertilizer application especially urea increase the N supply at flowering and pod filling stage, delay leaf aging, enhanced chlorophyll contents and photo assimilates [47]. The total amount of chlorophyll contents increased by the availability of nitro compounds in the rhizosphere and consequently to the plants, ultimately produced more assimilates via photosynthesis which directly related to improve growth and yield [9,48]. Growth regulators and chemical fertilizers were significantly effective in mitigating the drastic effects of drought by maintaining the water efficiency of canola plants and augmenting the accretion of osmolytes. Accumulation of osmolytes may also favors the improvement of photosynthetic and gas exchange attributes [15]. ~~The grain yield reduced due to water stress as reported in pervious study [53].~~

Field Code Changed

T~~he NPK treatment increases the flow of nutrient to the aerial part of plants and reduced the impact of water stress (Table 4,6). Due to water scarcity and loss of ionic balance, nutrients remained bond to the soil particles that are critical for the normal growth and development [49,50]. Same findings have been reported in the present work in the form of decreased biological yield (40%) with non-availability of nutrients (5-10%).~~

Field Code Changed

Canola oil is the commercial commodity, while its content, profile and composition are affected by drought stress as reported in the current work (Table 5). Seed oil stems mostly from photosynthesis and green silique walls, later carbon is routed through different metabolic pathways into triacylglycerol occurring in the chloroplast, cytosol, and endoplasmic reticula [51]. The current experiment suggests 3–11% decrease in oil content and oil yield in the *B. napus* when the plants were exposed to irrigation stress, but Aslam et al. [52] reported a mere ~~3.2%~~ reduction of 3.2%.

~~The grain yield reduced due to water stress as reported in pervious study [53].~~ The NPK treatment increases the flow of nutrient to the aerial part of plants and reduced the impact of water stress (Table ~~4,~~6). Due to water scarcity and loss of ionic balance, nutrients remained bond to the soil particles that are critical for the normal growth and development [49,50]. Same findings have been reported in the present work in the form of decreased biological yield (40%) with non-availability of nutrients (5-10%). Particularly, Nitrogen (N), phosphorus (P) and potassium (K) contents increased with foliarly applied PGRs followed by chemical fertlizers in canola. This might be accredited to the role of K in biochemical pathways in plants. Potassium has a positive effects on metabolic processes of nucleic acids and proteins (Bednarz & Oosterhuis, 1999). Phosphorus as a

Field Code Changed

Field Code Changed

constituent of cell nuclei is essential for cell division and development of meristematic tissue of cotton. Further, P has a well-known impact in photosynthesis as well as synthesis of nucleic acids, proteins, lipids and other essential compounds (Sawana et al., 2007). The percentage of NPK uptake enhanced with the combined application of NPK and PGRs (Table 5). The bio fertilizers improve the soil textures and bacterial colonization with the modification of physio-chemical properties of rhizosphere. On the other hand, the PGRs increased the photo assimilates translocation to sink (root tissue) and also improved the nutrient uptake and absorption power under water deficit and adverse environment condition. The PGRs can enhanced the activity of some vital N, P and K metabolizing enzymes in plant which enhanced NPK contents under different irrigation regimes [48,54].

All plants have been equipped with innate antioxidant enzyme mechanisms for the detoxification of reactive oxygen species. CAT decomposes H_2O_2 into water and molecular O_2. POX dismutases H_2O_2 by oxidizing co-substrates such as phenolic compounds and/or antioxidants and PPO in urn oxidizes phenols to chinone [55].

Membrane lipid peroxidation is a frequently used indictor to test the degree of plant sensitivity to oxidative damage caused by stress [56]. Our data revealed sensitivity of canola towards drought by elevation MDA content compared to F_2, F_3 treatments (Table 7). A lower level of lipid peroxidation presented high membrane stability (Table 7). It seems that the cell membrane integrity was maintained with chemical fertilizers and PGRs against the oxidative stress induced by water stress (Table 7). Mamnabi et al. [9] also suggested an amplification of antioxidant enzymes, total soluble sugars, photosynthetic attributes, leaf water content, membrane stability index and stomatal conductance but decreased the leaf temperature under different irrigation regimes.

The affirmative role of fertilizers and PGRs increase the antioxidant enzyme activities under water deficient condition in canola [57]. The PGRs treated plant showed more activities of antioxidant enzymes like POD, CAT and PPO as compared to untreated plants under moderate and severe water stress. The highest antioxidant activities were observed in T_3 treatments under severe stress as compared to T_1 and T_2 (Table 7). This supremacy was attained by the additive effect of fertilizer and PGRS on canola plants.

Conclusion

Canola is an emerging and unique oil seed crop among the other oil producing plants due to low erucic acid and glucosinolate contents. Here, we initially illustrate how NPK and PGRs, either individually or in combination, impact canola growth, photosynthetic and antioxidant activities and later seed yield and quality, and also attempt to explain its interaction to water scarcity for addressing these vital challenges. From the outcomes of current study, it appears rational to recommend chemical fertilizers (NPK) and PGRs (IAA and GA_3), that brought about better impact on canola seed yield, seed protein content, oil, oil composition with low glucosinolate and euracic acid contents, even under severe rate of evaporation (150mm).Drought is negatively affecting growth, chlorophyll contents and availability of nutrients to the canola which directly reducing seed yield and seed oil. The oxidative injuries caused by water stress resulted in diminution of chlorophyll content, MSI, and stomatal conductance of rapeseed plants. Application of growth promoting substances in canola enhanced growth, physiological response under different irrigation regimes. The harmful effects of stress were minimized considerably by the combined application of fertilizers and PGRs, thus improved growth attributes, chlorophyll content, MSI, stomatal conductance, antioxidants, osmoprotectants, grain yield and importantly, leading to a reduction in lipid peroxidation, particularly under moderate and severe rate of evaporation. These supremacies were attained by additive effects of NPK and PGPR, reducing the impact of drought. Such models can improve the probability of forecasting canola aptitude in challenging climates with an immediate response, but will also broadly help to select traits that can be further exploited through gene mining to produce sustainable and climate-resilient canola genotypes with considerable yield under high rate of water evaporation.

Author Contributions

Mohammad Aquil Siddiqui & Muhammad Mahran Aslam conceive the ideal, conducted the experiment, statistical analysis. and wrote the first draft of paper. Fozia Farhat conducted the analysis of physiological and biochemical parameters at Government College Women University Faisalabad .wrote first draft of the manuscript and finalized it. Shafquat Yasmeen and Muhammad Tahir Khan helped in collecting the agronomic data of the crop and formatting the paper. Mahboob Ali Sial & Imtiaz Ahmad Khan review the draft and provided technical inputs.

References

1. Anonymous. Food Outlook; Global Information and Early Warning System. FAO. Rome, Italy, 2015.
2. Mohtashami R, Movahhedi Dehnavi M, Balouchi H, Faraji H. Improving yield, oil content and water productivity of dryland canola by supplementary irrigation and selenium spraying. Agric. Water Manag. 2020; 232: 106046.
3.Anonymous. Rapeseed crop production. FAO. Rome, Italy, 2017. http://www. fao.org/ faostat/en/#data/QC.2014;Crops/Regions/Worldlist/ProductionQuantity

FAO (2017). FAOSTAT. Available online at: http://www.fao.org/faostat/en/#data/QC. 2014; Crops/Regions/World list/Production Quantity (pick lists) (Accessed December 22, 2017)

4. Walker KC, Booth EJ. Agricultural aspects of rape and other Brassica products. Eur. J. Lipid Sci. Technol. 2001; 103(7): 441–446.
5. Jahangir M, Abdel-Farid IB, Simoh S, Kim HK, Verpoorte R, Choi YH. Investigation of Brassica biochemical status by NMR-based metabolomics. *XXVIII International Horticultural Congress on Science and Horticulture for People (IHC2010): Int. Symposium* . 2010; 936*:* 163–172.
6. Mitrović PM, Stamenković OS, Banković-Ilić I, Djalović IG, Nježić ZB, et al. White Mustard (Sinapis alba L.) Oil in Biodiesel Production: A Review. Front. plant sci. 2020; 11: 299-299.
7. Elferjani R, Soolanayakanahally R. Canola responses to drought, heat, and combined stress: shared and specific effects on carbon assimilation, seed yield, and oil composition. Front. Plant Sci. 2018: 1–17. https://doi.org/10.3389/fpls.2018.01224
8. Champolivier L, Merrien A. Effects of water stress applied at different growth stages to Brassica napus L. var. oleifera on yield, yield components and seed quality. Eur. J. Agron.. 1996; 5(3–4): 153–160.
9. Aksouh-Harradj NM, Campbell LC, Mailer RJ. Canola response to high and moderately high temperature stresses during seed maturation. Canadian Journal of Plant Science. 2006; 86(4): 967–980.
10. Ahmar S, Liaqat N, Hussain M, Salim MA, Shabbir MA, Ali MY, Noushahi HA, Bilal M, Atta B, Rizwan M. Effect of abiotic stresses on Brassica species and role of transgenic breeding for adaptation. Asian J. Res. Crop Sci. 2019: 1–10.
11. Hussain HA, Hussain S, Khaliq A, Ashraf U, Anjum SA, et al. Chilling and Drought Stresses in Crop Plants: Implications, Cross Talk, and Potential Management Opportunities. Front. plant sci. 2018; 9: 393-393.
12. Urban L, Aarrouf J, Bidel LPR. Assessing the Effects of Water Deficit on Photosynthesis Using Parameters Derived from Measurements of Leaf Gas Exchange and of Chlorophyll a Fluorescence. Front. plant sci. 2017; 8: 2068-2068.
13. Mamnabi S, Nasrollahzadeh S, Ghassemi-Golezani K, Raei Y. Improving yield-related physiological characteristics of spring rapeseed by integrated fertilizer management under water deficit conditions. Saudi J. Biol. Sci. 2020; 27: 797-804.
14. Yin Z, Lu J, Meng S, Liu Y, Mostafa I, et al. Exogenous melatonin improves salt tolerance in tomato by regulating photosynthetic electron flux and the ascorbate–glutathione cycle. J. Plant. Interact. 2019; 14: 453-463.
15. Das K, Roychoudhury A. Reactive oxygen species (ROS) and response of antioxidants as ROS-scavengers during environmental stress in plants. Front. Environ. Sci. 2014: 2.

16. Rezayian M, Ebrahimzadeh H, Niknam V. Nitric Oxide Stimulates Antioxidant System and Osmotic Adjustment in Soybean Under Drought Stress. J.~~ournal of~~ Soil Sc~~ience~~i. ~~and~~ Plant. Nutr~~ition~~. 2020; 20: 1122-1132.

17. Khaleghi A, Naderi R, Brunetti C, Maserti BE, Salami SA, et al. ~~(2019)~~ Morphological, physiochemical and antioxidant responses of Maclura pomifera to drought stress. Sci.~~entific~~ Rep~~orts~~. 2019; 9: 19250.

18. Laxa M, Liebthal M, Telman W, Chibani K, Dietz K-J. ~~(2019)~~ The Role of the Plant Antioxidant System in Drought Tolerance. Antioxidants (Basel, Switzerland). 2019; 8: 94.

19. Sharma A, Shahzad B, Kumar V, Kohli SK, Sidhu GPS, et al. ~~(2019)~~ Phytohormones Regulate Accumulation of Osmolytes Under Abiotic Stress. Biomolecules. 2019; 9: 285.

20. Hasanuzzaman M, Bhuyan MHMB, Zulfiqar F, Raza A, Mohsin SM, et al. ~~(2020)~~ Reactive Oxygen Species and Antioxidant Defense in Plants under Abiotic Stress: Revisiting the Crucial Role of a Universal Defense Regulator. Antioxidants. 2020; 9: 681.

21. Khattab EA, Afifi MH, Amin GA. ~~(2019)~~ Significance of nitrogen, phosphorus, and boron foliar spray on jojoba plants. Bull~~etin~~. ~~of the~~ Nati~~onal~~ Res~~earch~~. Cen~~tre~~. 2019; ~~-~~43: 66.

22. Anjum NA, Gill SS, Umar S, Ahmad I, Duarte AC, et al. ~~(2012)~~ Improving Growth and Productivity of Oleiferous Brassicas under Changing Environment: Significance of Nitrogen and Sulphur Nutrition, and Underlying Mechanisms. ~~The~~ Sci~~entific~~. World J.~~ournal~~ 2012: 657808.

23. Yagoub SO, Ahmed WMA, Mariod. ~~AA (2012)~~ Effect of Urea, NPK and Compost on Growth and Yield of Soybean (Glycine max L.), in Semi-Arid Region of Sudan. ISRN Agronomy 2012: 678124.

24. Shirazi SM, Yusop Z, Zardari NH, Ismail Z. ~~(2014)~~ Effect of Irrigation Regimes and Nitrogen Levels on the Growth and Yield of Wheat. Adv~~ances in~~. Agric~~ulture~~. 2014: 250874.

25. Manik SMN, Pengilley G, Dean G, Field B, Shabala S, et al. ~~(2019)~~ Soil and Crop Management Practices to Minimize the Impact of Waterlogging on Crop Productivity. Front~~iers in~~. plant sci~~ence~~. 2019; 10: 140-140.

26. Rezaei R, Valadabadi SAR, Shirani Rad AH, Sayfzadeh S, Hadidi Masouleh E. ~~(2018)~~ Effects of biological fertilizers application and various amounts of urea fertilizers under water stress conditions on yield, nitrogen use efficiency and effective ingredients of Calendula officinalis L. Iranian. J.~~ournal of~~ Med.~~icinal and~~ Aromat~~ic~~. Plants Res~~earch~~. 2018; 34: 547-564.

27. Bhardwaj D, Ansari MW, Sahoo RK, Tuteja N. ~~(2014)~~ Biofertilizers function as key player in sustainable agriculture by improving soil fertility, plant tolerance and crop productivity. Microb~~ial~~. cell. fact~~ories~~. 2014; ~~-~~13: 66-66.

28. Mondal T, Datta J, Mondal N. ~~(2017)~~ Chemical fertilizer in conjunction with biofertilizer and vermicompost induced changes in morpho-physiological and bio-chemical traits of mustard crop. J~~ournal of th~~.e Saudi Soc~~iety~~. ~~of~~ Agric~~ultural~~. Sci~~ences~~. 2017; 16: 135-144.

29. Sevinç K. ~~(2018)~~ Effects of Vermicompost on Some Morphological, Physiological and Biochemical Parameters of Lettuce (Lactuca sativa var. crispa) under Drought Stress. Not Bot Horti Agrobot Cluj Napoca. 2018:~~Notulae Botanicae Horti Agrobotanici Cluj-Napoca~~ 47.

30. Mukhongo RW, Tumuhairwe JB, Ebanyat P, AbdelGadir AH, Thuita M, et al. ~~(2017)~~ Combined Application of Biofertilizers and Inorganic Nutrients Improves Sweet Potato Yields. Front.~~iers in~~ Plant. Sc~~i~~i~~ence~~. 2017; 8.

Formatted: Font: (Default) Times New Roman, 12 pt, Font color: Auto, Pattern: Clear

31. Stalikas CD. ~~(2007)~~ Extraction, separation, and detection methods for phenolic acids and flavonoids. J. Sep. Sci. 2017; 30: 3268-3295.
32. Rathje. ~~(1959)~~ Jackson, M. L.: Soil chemical analysis. Verlag: Prentice Hall, Inc., Englewood Cliffs, NJ. 1958, 498 S. DM 39.40. Zeitschrift für Pflanzenernährung, Düngung, Bodenkunde. 1959; ~~-~~85: 251-252.
33. Pimstein A, Karnieli A, Bansal SK, Bonfil DJ. ~~(2011)~~ Exploring remotely sensed technologies for monitoring wheat potassium and phosphorus using field spectroscopy. Field. Crops. Res~~earch~~. 2011; 121: 125-135.
34. Tammam AA. ~~(2008)~~ Study of salt tolerance in wheat (*Triticum aestium* L.) cultivar Banysoif 1. Australian journal of crop science. 2008; 1: ~~v. 1: pp.~~ 115-125 ~~2008 v.2001 no.2003~~.
35. Bremner JM, Mulvaney CS. Nitrogen total. P.595-62. In: Methods of Soil Analysis. Part 2: 2nd edition. A.L. Page, R.H. Miller and D.R. Keeney (eds.) American Society of Agronomy, Madison, WI. USA. 1982.
36. Arnon DI. ~~(1949)~~ C~~c~~opper enzymes in isolated chloroplasts. Polyphenoloxidase in beta vulgaris. Plant physiol~~ogy~~. 1949; 24: 1-15.
37. Ghassemi-Golezani K, Ghassemi, S., Yaghoubian, I. ~~(2016)~~ Salicylic Acid regulate Physiological Performance of milk thistle (Silybum marianum L.) under water stress. Adv Biores. 2016; 7(4):~~:~~ 34-40.
38. Kochert G. ~~(1978)~~ Carbohydrate determination by the phenol-sulfuric acid method. Handbook. Phycol Meth. 1978: 95-97.
39. Bates LS, Waldren RP, Teare ID. ~~(1973)~~ Rapid determination of free proline for water-stress studies. Plant and Soil. 1973; 39: 205-207.
40. Kumar KB, Khan, P.A. ~~(1982)~~ Peroxidase and polyphenoloxidase in excisedragi (Eleusine coracana cv. PR 202) leaves during senescence~~.~~. ~~The~~ Indian J.~~ournal of~~ Exp.~~erimental~~ Biol.~~ogy:~~ 1982: 412–416.
41. Singh BK, Sharma SR, Singh B. ~~(2010)~~ Antioxidant enzymes in cabbage: Variability and inheritance of superoxide dismutase, peroxidase and catalase. Scientia Horticulturae. 2010; 124: 9-13.
42. Gueta-Dahan Y, Yaniv Z, Zilinskas BA, Ben-Hayyim G. ~~(1997)~~ Salt and oxidative stress: similar and specific responses and their relation to salt tolerance in citrus. Planta. 1997; 203: 460-469.
43. Janero DR. ~~(1990)~~ Malondialdehyde and thiobarbituric acid-reactivity as diagnostic indices of lipid peroxidation and peroxidative tissue injury. Free Radic Biol Med. 1990; ~~-~~9: 515-540.
44. Gomez KAaG, A. ~~(1984)~~ Statistical Procedure for Agricultural Research—Hand Book. John Wiley & Sons New york. 1984.
45. Shahid MA, Sarkhosh A, Khan N, Balal RM, Ali S, et al. ~~(2020)~~ Insights into the Physiological and Biochemical Impacts of Salt Stress on Plant Growth and Development. Agronomy. 2020; ~~-~~10: 938.
46. Sharif P, Seyedsalehi M, Paladino O, Van Damme P, Sillanpää M, et al. ~~(2018)~~ Effect of drought and salinity stresses on morphological and physiological characteristics of canola. Int~~ernational~~. J.~~ournal of~~ Environ.~~mental~~ Sci~~ence and~~. Technol~~ogy~~. 2018; 15: 1859-1866.
47. Flexas J, Medrano H. ~~(2002)~~ Drought-inhibition of photosynthesis in C3 plants: stomatal and non-stomatal limitations revisited. Ann. Bot. 2002; 89: 183-189.
48. Sehgal, A., Sita, K., Siddique, K. H. M., Kumar, R., Bhogireddy, S., Varshney, R. K., HanumanthaRao, B., Nair, R. M., Prasad, P. V. V., & Nayyar, H. (2018). Drought or/and heat-stress effects on seed filling in food crops: Impacts on functional biochemistry, seed

Formatted: Font: Italic

yields, and nutritional quality. *Frontiers in Plant Science*, *871*(November), 1–19. https://doi.org/10.3389/fpls.2018.01705.

49. Wilkinson S, Davies WJ. ~~(2010)~~ Drought, ozone, ABA and ethylene: new insights from cell to plant to community. Plant Cell Environ. 2010; 33: 510-525.
50. Pantin F, Monnet F, Jannaud D, Costa JM, Renaud J, et al. ~~(2013)~~ The dual effect of abscisic acid on stomata. New Phytol~~ogist~~. 2013; 197: 65-72.
51. Yan W, Zhong Y, Shangguan Z. ~~(2016)~~ A meta-analysis of leaf gas exchange and water status responses to drought. Sci.~~entific~~ Rep~~orts~~. 2016; 6: 20917.
52. Gai Z, Zhang J, Li C. ~~(2017)~~ Effects of starter nitrogen fertilizer on soybean root activity, leaf photosynthesis and grain yield. PLo~~O~~S ONE. 2017. 12: ~~e~~0174841.
53. Razaq M, Zhang P, Shen H-l, Salahuddin. ~~(2017)~~ Influence of nitrogen and phosphorous on the growth and root morphology of Acer mono. PLo~~O~~S ONE. 2017; 12: ~~e~~0171321.
54. Baud S, Lepiniec L. ~~(2010)~~ Physiological and developmental regulation of seed oil production. Prog. Lipid Res. 2010; 49: 235-249.
55. Aslam MN, Nelson MN, Kailis SG, Bayliss KL, Speijers J, et al. ~~(2009)~~ Canola oil increases in polyunsaturated fatty acids and decreases in oleic acid in drought-stressed Mediterranean-type environments. Plant Breed~~ing~~. 2009; 128: 348-355.
56. Elferjani R, Soolanayakanahally R. ~~(2018)~~ Canola Responses to Drought, Heat, and Combined Stress: Shared and Specific Effects on Carbon Assimilation, Seed Yield, and Oil Composition. Front~~iers~~. ~~in~~ Plant Sci~~ence~~. 2018; 30(9):1224. ~~9.~~
57. Barnabás B, Jäger K, Fehér A. ~~(2008)~~ The effect of drought and heat stress on reproductive processes in cereals. Plant Cell Environ. 2008; 31: 11-38.
58. Bednarz CW, Oosterhuis DM. Physiological changes associated with potassium deficiency in cotton. J. Plant Nutr. 1999; 22(2): 303–313.
59. Sawana ZM, Hafez SA, Alkassas AR . Nitrogen, potassium and plant growth retardant effects on oil content and quality of cotton seed. Grasas Y Aceites. 2007; 58(3): 243–251.
60. Waqas MA, Khan I, Akhter MJ, Noor MA, Ashraf U. ~~(2017)~~ Exogenous application of plant growth regulators (PGRs) induces chilling tolerance in short-duration hybrid maize. Environ~~mental~~. Sci~~ence~~. ~~and~~ Pollut~~ution~~. Res~~earch~~. 2017; ~~-~~24: 11459-11471.

61. Apel K, Hirt H. ~~(2004)~~ Reactive oxygen species: metabolism, oxidative stress, and signal transduction. Annu. Rev. Plant Biol. 2004; 55: 373-399.
62. Blokhina O, Virolainen E, Fagerstedt KV. ~~(2003)~~ Antioxidants, oxidative damage and oxygen deprivation stress: a review. Ann. Bot. 2004; 91 ~~Spec No~~: 179-194.
63. Khan N, Bano A, Babar MDA. ~~(2020)~~ Impacts of plant growth promoters and plant growth regulators on rainfed agriculture. PloS one. 2020; 15: e0231426-e0231426.

Formatted: Font: (Default) Times New Roman, 12 pt, Font color: Auto, Pattern: Clear

Commented [MA1]: Add new refernces

Physiological and Biochemical Exploration of Canola for Overcoming Yield Limitation by Integrated Fertilizer management under different water regimes

Table 1: Weather data of experimental location during 2016-17

**Month**	**T_{max} ^0C**		**T_{min} ^0C**		**Total Rain fall (mm)**		**R.H (%)**	
	2018-19	2019-20	2018-19	2019-20	2018-19	2019-20	2018-19	2019-20
**October**	37.1	38.4	20.6	19.5	0	0	57	53
**November**	32.6	31.20	12.9	12.6	0	0	52	51
**December**	28.5	25.6	11.2	8.0	0	0	56	54
**January**	25.4	22.1	10.3	7.3	0	2.0	60	61
**February**	21.4	28.2	9.2	10.0	0	0	44	49
**March**	34.1	34.4	17.1	14.5	0	0	47	47
**April**	39.0	40.4	20.9	19.9	0	0	43	42

Table 2: **Soil properties of experimental Field**

**Site**	**OM (%)**	**pH**	**meq/100g soil**				**µg/g soil**			
			Na	K	Mg	S	Zn	B	P	Ca
**NIA, Tandojam Exp. Farm**	2	6.5-7.0	0.08	0.11	3.2	16.90	1.40	0.34	10.83	2.5

Table 3: Variation in agronomical and yield attributes of Canola with fertilizers and Plant growth regulators under different water regimes

Irrigation	Treatment with Elicitors	Plant Height (cm)	Number of branches/plant	Leaf Area (cm^2)	Days to Flower	Number of seeds/plant	Biological yield/Plant (Kg)	1000 seed weight(g)	Seed yield (Kg/ha)	Grain Yield (gr/m)
**I_1 Normal**	T_0	114^{c}	5.60^{d}	90.5^{d}	68^{b}	160^{d}	5.30^{d}	3.98^{d}	1804^{d}	188^{g}
	T_1	114.5^{b}	5.73^{c}	91.3^{c}	68^{b}	171^{c}	5.38^{c}	4.28^{c}	2073^{b}	264^{a}
	T_2	115^{a}	5.78^{b}	92.4^{b}	69^{a}	176^{b}	5.36^{b}	4.33^{b}	2060^{c}	220^{d}
	T_3	113^{d}	5.85^{a}	94.0^{a}	66^{c}	188^{a}	5.43^{a}	4.96^{a}	2450^{a}	249^{b}
**I_2 Mild evaporation**	T_0	113^{b}	5.66^{d}	89.0^{d}	70^{a}	164^{d}	5.6^{a}	4.0^{d}	1800^{d}	176.4^{h}
	T_1	114^{a}	5.81^{b}	90.0^{c}	67^{c}	170^{b}	5.20^{b}	4.15^{c}	2096^{c}	232.42^{c}
	T_2	114^{a}	5.80^{c}	90.5^{b}	68^{b}	169^{c}	5.16^{d}	4.19^{b}	2118^{b}	207.24^{e}
	T_3	113^{b}	5.83^{a}	91.0^{a}	65^{d}	178^{a}	5.29^{c}	4.23^{a}	2340^{a}	221.18^{bc}
**I_3 Moderate evaporation**	T_0	112^{b}	5.58^{d}	86.4^{d}	70^{a}	165^{d}	4.94^{d}	3.90^{d}	1650^{e}	131.06^{k}
	T_1	112^{b}	5.78^{c}	89.5^{c}	65^{c}	169^{c}	5.14^{c}	4.18^{b}	1943^{b}	165.97^{i}
	T_2	113^{a}	5.80^{b}	90.0^{b}	67^{b}	172^{b}	5.19^{b}	4.09^{c}	1940^{c}	172.03^{h}
	T_3	112^{b}	5.82^{a}	93.0^{a}	64^{d}	178^{a}	5.27^{a}	4.26^{a}	2353^{a}	198.21^{f}
**I_4 Severe evaporation**	T_0	113^{a}	5.52^{d}	85.8^{d}	69^{a}	150^{d}	3.89^{d}	3.67^{d}	1538^{f}	170.52^{h}
	T_1	112^{b}	5.68^{c}	88.4^{b}	67^{c}	168^{b}	5.16^{b}	4.13^{c}	1923^{c}	173.23^{h}
	T_2	113^{a}	5.70^{b}	87.0^{c}	68^{b}	163^{c}	5.14^{c}	4.15^{b}	1975^{b}	133.05^{k}
	T_3	111^{c}	5.79^{a}	92.3^{a}	62^{d}	176^{a}	5.32^{a}	4.26^{a}	2318^{a}	156.84^{j}
**F $_{test}$**	I × T Interaction	1.23*	2.65**	0.023ns	7.75*	68.16**	1.27**	0.53*	30.64**	1803.26**

Note: I_1= 60mm evaporation, I_2= 100mm evaporation, I_3= 120mm evaporation, I_4= 150mm evaporation, T_0= No treatment, T_1= NPK, T_2= PGRs, T_3= (T_1+T_2). The alphabetical superscript in a column present significant difference among the treatments to different rate of evaporation from highest to lowest value (a=highest value). *= least significant, **significant, *** highly significant

Formatted: Font color: Blue

Table 4: Variation in Physiological attributes of Canola with chemical fertilizers and Plant growth regulators under different water regimes

Irrigation	Treatments	Chl a	Chl b	Total Chlorophyll	LWC	LT	Stomatal Conductance
		(mg/g FW)			(%)	(^{0}C)	(mmol m-^{2} s^{-1})
**I_1 Normal**	T_0	1.43^{f}	0.834^{c}	2.264^{d}	80.0^{ab}	21.8^{i}	142.3^{c}
	T_1	2.18^{a}	0.87^{a}	3.05^{a}	83.0^{a}	19.7^{l}	146.2^{a}
	T_2	1.63^{e}	0.84^{b}	2.47^{d}	82.0^{a}	21.6^{j}	$144. 1^{b}$
	T_3	1.95^{b}	0.85^{b}	2.8^{c}	81.9^{a}	19.8^{k}	146.42^{a}
**I_2 Mild evaporation**	T_0	1.28^{h}	0.78^{d}	2.06^{f}	80.82^{ab}	25.6^{f}	140.73^{d}
	T_1	1.88^{c}	0.82^{c}	2.7^{c}	81.80^{a}	22.3^{h}	145.12^{a}
	T_2	1.48^{f}	0.80^{c}	2.28^{d}	81.17^{a}	23.5^{g}	144.95^{b}
	T_3	1.72^{d}	0.80^{c}	2.52^{b}	82.0^{a}	21.8^{i}	143.91^{b}
**I_3 Moderate evaporation**	T_0	0.73^{k}	0.72^{f}	1.45^{i}	74.17^{de}	30.6^{c}	101.0^{i}
	T_1	1.18^{i}	0.75^{e}	1.93^{g}	76.16^{cd}	28.5^{d}	107.9^{h}
	T_2	1.18^{i}	0.75^{e}	1.93^{g}	78.18^{bc}	27.6^{e}	117.26^{f}
	T_3	1.39^{g}	0.76^{e}	2.15^{e}	78.84^{bc}	25.91^{f}	119.47^{e}
**I_4 Severe evaporation**	T_0	0.47^{m}	0.68^{g}	1.15^{l}	62.49^{f}	35.0^{a}	70.94^{k}
	T_1	0.66^{l}	0.71^{f}	1.37^{k}	65.18^{f}	33.4^{b}	78.61^{j}
	T_2	0.85^{k}	0.70^{f}	1.55^{j}	71.80^{e}	28.92^{d}	97.67^{i}
	T_3	1.13^{j}	0.72^{f}	1.85^{h}	73.6^{de}	27.9^{e}	110.4^{g}
**F_{test}**	I × T interaction	0.134**	0.00032**	1.982**	0.139**	21.99**	143.4**

Note: I_1= 60mm evaporation, I_2= 100mm evaporation, I_3= 120mm evaporation, I_4= 150mm evaporation, T_0= No treatment, T_1= NPK, T_2= PGRs, T_3= (T_1+T_2). The alphabetical superscript in a column present significant difference among the treatments to different rate of evaporation from highest to lowest value (a=highest value). *= least significant, **significant, *** highly significant

Formatted Table

Formatted: Font color: Blue

Table 5: Variation in quality traits of Canola with chemical fertilizers and Plant growth regulators under different water regimes

Irrigation	Treatments	Oil content (%)	Oil yield (kg ha^{1})	Protein (%)	Moisture (%)	Glucosionalate (µmol/g)	Erucic acid (%)
**I_1 Normal**	T_0	38.0^{d}	238.0^{d}	20.77^{d}	5.23^{d}	26.2^{a}	4.44^{a}
	T_1	38.5^{c}	324.5^{c}	24.82^{c}	5.72^{c}	22.0^{c}	4.20^{b}
	T_2	39.0^{b}	328.9^{b}	24.97^{b}	5.96^{b}	18.7^{d}	3.74^{c}
	T_3	42.8^{a}	468.0^{a}	25.07^{a}	6.77^{a}	14.6^{f}	3.22^{d}
**I_2 Mild evaporation**	T_0	37.0^{d}	234.6^{d}	20.0^{d}	5.0^{d}	24.0^{b}	5.21^{a}
	T_1	37.8^{c}	340.0^{c}	24.82^{c}	5.36^{b}	20.0^{c}	4.37^{b}
	T_2	38.2^{b}	355.0^{b}	24.79^{b}	5.30^{c}	19.0^{d}	3.98^{c}
	T_3	42.5^{a}	437.8^{a}	25.0^{a}	5.98^{a}	16.0^{e}	3.41^{d}
**I_3 Moderate evaporation**	T_0	36.0^{d}	229.0^{d}	18.5^{d}	4.93^{c}	23.5^{b}	4.98^{a}
	T_1	37.3^{c}	324.5^{c}	23.0^{c}	5.11^{c}	17.0^{e}	3.75^{c}
	T_2	37.9^{b}	329.38^{b}	23.5^{b}	5.19^{b}	18.2^{d}	3.82^{b}
	T_3	41.6^{a}	398.0^{a}	24.8^{a}	5.75^{a}	14.0^{f}	3.45^{d}
**I_4 Severe evaporation**	T_0	34.8^{d}	220.0^{d}	18.0^{d}	4.81^{d}	20.0^{c}	4.99^{a}
	T_1	36.0^{c}	378.3^{c}	23.0^{b}	5.25^{c}	18.0^{d}	3.68^{c}
	T_2	36.5^{b}	382.0^{b}	22.5^{c}	5.29^{b}	16.5^{e}	3.71^{b}
	T_3	41.0^{a}	437.2^{a}	24.2^{a}	5.78^{a}	11.6^{g}	3.50^{d}
**F_{test}**	I × T	13.68**	6.34*	3.36**	0.985**	4.28*	12.78**

Note: I_1= 60mm evaporation, I_2= 100mm evaporation, I_3= 120mm evaporation, I_4= 150mm evaporation, T_0= No treatment, T_1= NPK, T_2= PGRs, T_3= (T_1+T_2). The alphabetical superscript in a column present significant difference among the treatments to different rate of evaporation from highest to lowest value (a=highest value). *= least significant, **significant, *** highly significant

Formatted: Font color: Blue

Table 6: Variation in Nitrogen (N), Phosphorus (P) and Potassium (K) of Canola with chemical fertilizers and Plant growth regulators under different water regimes

Irrigation	Treatments	N (%)	P (%)	K (%)	N uptake (kg ha^{1})	P uptake (kg ha^{1})	K uptake (kg ha^{1})
**I_1 Normal**	T_0	2.70^{d}	0.18^{d}	1.21^{d}	22.81^{d}	17.43^{d}	16.34^{d}
	T_1	3.72^{b}	0.21^{b}	1.68^{b}	38.45^{b}	21.09^{b}	18.23^{b}
	T_2	3.59^{c}	0.20^{c}	1.55^{c}	34.23^{c}	19.94^{c}	17.78^{c}
	T_3	3.88^{a}	0.24^{a}	1.88^{a}	51.84^{a}	23.29^{a}	22.67^{a}
**I_2 Mild evaporation**	T_0	2.69^{d}	0.20^{c}	1.20^{d}	22.86^{d}	18.43^{d}	16.0^{d}
	T_1	3.35^{c}	0.20^{c}	1.55^{b}	38.98^{b}	20.45^{b}	18.45^{b}
	T_2	3.54^{b}	0.21^{b}	1.43^{c}	29.74^{c}	19.90^{c}	18.21^{c}
	T_3	3.75^{a}	0.22^{a}	1.78^{a}	50.95^{a}	22.45^{a}	21.45^{a}
**I_3 Moderate evaporation**	T_0	2.71^{d}	0.19^{c}	1.16^{d}	20.09^{d}	17.09^{d}	14.76^{d}
	T_1	3.15^{c}	0.22^{a}	1.61^{b}	30.56^{b}	19.90^{b}	17.85^{c}
	T_2	3.19^{b}	0.21^{b}	1.34^{c}	26.64^{c}	18.64^{c}	17.93^{b}
	T_3	3.84^{a}	0.21^{b}	1.72^{a}	49.80^{a}	21.98^{a}	20.06^{a}
**I_4 Severe evaporation**	T_0	2.55^{d}	0.17^{c}	1.03^{d}	17.65^{d}	15.43^{d}	13.49^{d}
	T_1	3.38^{c}	0.21^{b}	1.45^{c}	30.32^{c}	18.45^{c}	17.83^{c}
	T_2	3.45^{b}	0.21^{b}	1.54^{b}	33.90^{b}	19.05^{b}	16.98^{b}
	T_3	3.78^{a}	0.24^{a}	1.69^{a}	48.73^{a}	20.97^{a}	19.56^{a}
**F_{test}**	I × T	3.05**	0.168***	0.98**	16.24*	9.34***	3.44**

Note: I_1= 60mm evaporation, I_2= 100mm evaporation, I_3= 120mm evaporation, I_4= 150mm evaporation, T_0= No treatment, T_1= NPK, T_2= PGRs, T_3= (T_1+T_2). The alphabetical superscript in a column present significant difference among the treatments to different rate of evaporation from highest to lowest value (a=highest value). *= least significant, **significant, *** highly significant

Formatted Table

Formatted: Font color: Blue

Table 7: Variation in Biochemical attributes of Canola with chemical fertilizers and Plant growth regulators under different water

Irrigation	Treatments	PPO	CAT	POX (U g^{-1} FW)	SOD	MDA (mmol g^{-1} FW)	Soluble Sugar (mg /g DW)	Proline (mmol g^{-1} FW)	MSI
I_1 Normal	T_0	0.47^{f}	0.21^{h}	0.15^{f}	0.26^{g}	2.4^{j}	31.4^{ijk}	15.4^{ij}	86.71^{b}
	T_1	0.54^{f}	0.23^{h}	0.17^{f}	0.31^{fg}	2.29^{j}	30.0^{k}	16^{ij}	87.92^{a}
	T_2	0.52^{f}	0.26^{h}	0.16^{f}	0.33^{fg}	2.28^{j}	31.0^{ijk}	15.03^{ij}	87.81^{a}
	T_3	0.57^{f}	0.24^{h}	0.18^{f}	0.32^{fg}	2.27^{j}	30.2^{jk}	15.07^{ij}	87.45^{a}
I_2 Mild evaporation	T_0	0.79^{f}	0.6^{gh}	$o.36^{ef}$	0.59^{efg}	3.5^{gh}	32.8^{ij}	16.36^{hi}	85.0^{c}
	T_1	0.80^{f}	0.64^{g}	0.88^{e}	0.63^{efg}	3.07^{hi}	31.63^{ijk}	16.1^{ij}	$87. 25^{ab}$
	T_2	1.02^{f}	0.65^{g}	0.95^{e}	0.74^{e}	2.66^{ij}	32.24^{ijk}	15.8^{ij}	88.0^{a}
	T_3	1.08^{f}	1.38^{fg}	1.57^{d}	$o.79^{e}$	2.44^{j}	32.87^{i}	15.57^{ij}	88.07^{a}
I_3 Moderate evaporation	T_0	1.88^{e}	2.0^{f}	1.67^{d}	1.28^{d}	5.9^{bc}	38.56^{h}	22.8^{c}	75.62^{g}
	T_1	2.03^{e}	2.08^{f}	1.86^{d}	1.31^{d}	5.29^{ed}	43.6^{f}	21.9^{bc}	78.45^{f}
	T_2	2.82^{cd}	3.15^{d}	2.69^{c}	2.10^{c}	4.34^{f}	45.64^{ef}	18.14^{fg}	80.05^{e}
	T_3	3.38^{bc}	3.56^{c}	2.87^{c}	2.18^{c}	3.72^{g}	49.07^{d}	17.34^{gh}	81.98^{d}
I_4 Severe evaporation	T_0	1.9^{e}	2.75^{e}	2.84^{c}	1.96^{c}	7.30^{a}	41.0^{g}	32.68^{a}	64.09^{i}
	T_1	2.15^{e}	2.85^{e}	3.12^{bc}	1.90^{c}	6.34^{b}	43.52^{f}	27.44^{b}	65.32^{i}
	T_2	3.63^{b}	4.07^{b}	3.85^{b}	2.95^{b}	5.57^{cd}	63.36^{b}	22.08^{cd}	70.29^{h}
	T_3	4.19^{a}	4.39^{a}	4.44^{a}	3.44^{a}	5.72^{cd}	67.62^{a}	21.4^{de}	74.49^{g}
F_{test}	I × T interaction	0.612**	0.49**	0.317**	0.34**	0.509**	99.01**	14.49**	14.22**

regimes

Note: I_1= 60mm evaporation, I_2= 100mm evaporation, I_3= 120mm evaporation, I_4= 150mm evaporation, T_0= No treatment, T_1= NPK, T_2= PGRs, T_3= (T_1+T_2). The alphabetical superscript in a column present significant difference among the treatments to different rate of evaporation from highest to lowest value (a=highest value). *= least significant, **significant, *** highly significant

Formatted: Font color: Blue

Reviewer 1	Response by the Author/s
Authors must provide the full form of any abbreviation used in the study at first usage. For instance, the full form of MDA was not provided at the first use. Kindly confirm the same for all other abbreviations used in the article	Thank you. All the Abbreviations have been carefully tracked for correction
The authors must use line numbers while submitting an article to the journal. It is difficult for any reviewer to mention the specific desired changes without line numbers.	Added Line number
I also see various spacing issues in the article. For instance, have a look at Page 3; Paragraph 2. Please fix all such issues throughout the MS	Corrected the text
Check the citation of Table 7 in the article and correct it.	Corrected the citation in the discussion part
I assume that there is a typo in the title of Table 1. Please fix it	Corrected it
The authors should also make sure that the text font is the same throughout. I found certain instances where the font was not the same	Text Font has been corrected
The acknowledgements section is not in fact presenting “Acknowledgements”. The section describes author contributions instead.	Acknowledgement replace by Author contributions
**Reviewer 2**	**Response by the Author/s**
The author didn’t added line number , so it is very difficult to comment on some specific parts or lines where have some issues, generally	Thank you. Line Number added
I have added some comments in the PDF file, please carefully read the comments and similarly revise the whole MS.	MS revised carefully in the light of comments received
The article need to be revised fully, precisely focusing on the results presentation, discussion and conclusion	Major revision done precisely focusing on result, discussion and conclusion part (A separate file with track changes attached)
Abstract should be revised carefully and adopt the scientific style …	Abstract revised to ensure scientific styling
The article should be revised logically to improve the results and discussion section particularly, conclusion is not good, should be revised.	Results justified logically from the already published literature
I would recommend that all the table results should be used to draw figures to demonstrate the results significantly.	Presentation of results in the form of tables will be easy to understand for the readers.

	It is multifactorial experiment, so may present a confusion to exactly figure out the findings from the figures
Reference style is not in one standard format, revise it and follow the journal style	Reference arranged according to the journal requirement
