# Supplementary material for: Exploration of physiological and biochemical processes of canola with exogenously applied fertilizers and plant growth regulators under drought stress
Source: PLoS One. 2021 Dec 20;16(12):e0260960. doi: 10.1371/journal.pone.0260960 (PMC8687561; doi:10.1371/journal.pone.0260960)
Supplement: Attachment — Submitted filename: Review.docx [file pone.0260960.s002.docx]

**Review of ““Physiological and Biochemical Exploration of Canola for Overcoming Yield Limitation by Integrated Fertilizer management under different water regimes” for PolsOne.**

This manuscript describes a study entitled “Physiological and Biochemical Exploration of Canola for Overcoming Yield Limitation by Integrated Fertilizer management under different water regimes”.

This manuscripts describes the Environmental stress and its effects on the nutritional profile and crops economic value. Authors evaluated the nutritional quality and physio-biochemical modulation in *Brassica napus* L. (canola) from early growth to seed stage with different fertilizers and PGRs in different water regimes. They performed the experiment following randomized complete block design (RCBD) with four treatments (Control, Chemical fertilizers [Nitrogen (N) 90 kg/ha), Phosphorus (P) and Potassium (K) 45 kg/ha], PGRs indole acetic acid (IAA) 15g/ha, gibberellic acid (GA3) 15g/ha and the combination of NPK and PGRs] under different irrigations regimes (60, 100, 120, 150 mm evaporations). The results revealed positive interaction of applied elicitors to the water stress in canola except leaf area and depicted that the combination of NPK with PGRs improved the various morpho-physiological as well as biochemical parameters and improve grain yield and oil contents of canola in field.

I would like to express appreciation to the authors for this study, I found the manuscript very interesting to read, but also came across few issues that will deserve some careful attention.

1. The author didn’t added line number , so it is very difficult to comment on some specific parts or lines where have some issues, generally I have added some comments in the PDF file, please carefully read the comments and similarly revise the whole MS.
2. The article need to be revised fully, precisely focusing on the results presentation, discussion and conclusion.
3. Abstract should be revised carefully and adopt the scientific style …..
4. The article should be revised logically to improve the results and discussion section particularly, conclusion is not good, should be revised.
5. I would recommend that all the table results should be used to draw figures to demonstrate the results significantly.
6. Reference style is not in one standard format, revise it and follow the journal style.
7. Overall, I recommend the article should be published after major revision of the papers results and English improvement.
