# Supplementary material for: Exploration of physiological and biochemical processes of canola with exogenously applied fertilizers and plant growth regulators under drought stress
Source: PLoS One. 2021 Dec 20;16(12):e0260960. doi: 10.1371/journal.pone.0260960 (PMC8687561; doi:10.1371/journal.pone.0260960)
Supplement: Attachment — Submitted filename: Rebuttal Letter.docx [file pone.0260960.s004.docx]

| Reviewer 1 | Response by the Author/s |
| --- | --- |
| Authors must provide the full form of any abbreviation used in the study at first usage. For instance, the full form of MDA was not provided at the first use. Kindly confirm the same for all other abbreviations used in the article | Thank you. All the Abbreviations have been carefully tracked for correction |
| The authors must use line numbers while submitting an article to the journal. It is difficult for any reviewer to mention the specific desired changes without line numbers. | Added Line number |
| I also see various spacing issues in the article. For instance, have a look at Page 3; Paragraph 2. Please fix all such issues throughout the MS | Corrected the text |
| Check the citation of Table 7 in the article and correct it. | Corrected the citation in the discussion part |
| I assume that there is a typo in the title of Table 1. Please fix it | Corrected it |
| The authors should also make sure that the text font is the same throughout. I found certain instances where the font was not the same | Text Font has been corrected |
| The acknowledgements section is not in fact presenting “Acknowledgements”. The section describes author contributions instead. | Acknowledgement replace by Author contributions |
| Reviewer 2 | **Response by the Author/s** |
| The author didn’t added line number , so it is very difficult to comment on some specific parts or lines where have some issues, generally | Thank you. Line Number added |
| I have added some comments in the PDF file, please carefully read the comments and similarly revise the whole MS. | MS revised carefully in the light of comments received |
| The article need to be revised fully, precisely focusing on the results presentation, discussion and conclusion | Major revision done precisely focusing on result, discussion and conclusion part (A separate file with track changes attached) |
| Abstract should be revised carefully and adopt the scientific style … | Abstract revised to ensure scientific styling |
| The article should be revised logically to improve the results and discussion section particularly, conclusion is not good, should be revised. | Results justified logically from the already published literature |
| I would recommend that all the table results should be used to draw figures to demonstrate the results significantly. | Presentation of results in the form of tables will be easy to understand for the readers.  It is multifactorial experiment, so may present a confusion to exactly figure out the findings from the figures |
| Reference style is not in one standard format, revise it and follow the journal style | Reference arranged according to the journal requirement |
