# Supplementary material for: Exploration of physiological and biochemical processes of canola with exogenously applied fertilizers and plant growth regulators under drought stress
Source: PLoS One. 2021 Dec 20;16(12):e0260960. doi: 10.1371/journal.pone.0260960 (PMC8687561; doi:10.1371/journal.pone.0260960)
Supplement: Attachment — Submitted filename: Review_PONE-D-21e.docx [file pone.0260960.s005.docx]

**Review of ““Physiological and Biochemical Exploration of Canola for Overcoming Yield Limitation by Integrated Fertilizer management under different water regimes” for PolsOne.**

This manuscript describes a study entitled “Physiological and Biochemical Exploration of Canola for Overcoming Yield Limitation by Integrated Fertilizer management under different water regimes”.

I would like to express my appreciation to the authors for revising the MS, I found that manuscript has been improved and much better than earlier version, but also came across few issues that still deserve some careful attention. I have highlighted few places and would suggest to read carefully the entire MS and revise the English grammar, abbreviation and formatting. Overall, I recommend the article should be published after revision of these minor issues to improve the quality of their work.
