# Supplementary material for: Exploration of physiological and biochemical processes of canola with exogenously applied fertilizers and plant growth regulators under drought stress
Source: PLoS One. 2021 Dec 20;16(12):e0260960. doi: 10.1371/journal.pone.0260960 (PMC8687561; doi:10.1371/journal.pone.0260960)
Supplement: Attachment — Submitted filename: Rebuttal Letter_2.docx [file pone.0260960.s007.docx]

| Reviewer 3 | Response by the Author/s |
| --- | --- |
| The Reviewer suggested to change the title “Physiological and Biochemical Exploration of Canola for Overcoming Yield Limitation by Integrated Fertilizer management under different water regimes” of the Manuscript | Thank you, improved for better understanding of the contents of the manuscript as  “Exploration of Physiological and Biochemical Process of Canola with exogenously applied NPK and Plant Growth Regulators under Drought Stress” |
| Environmental stressors replaced by | Environmental stresses |
| Replace modify | alter |
| phyto-hormones | phytohormones |
| Chemical fertilizers are not a natural source. | Thank you! Corrected |
| Key interest replaced by | Major aim |
| What is seed stage? Please elaborate and correct. | The Abstract section shows collection of data from early stage to seed stage (maturity) as mentioned against major aim of this study. However, detailed application of fertilizers and Plant growth regulators mentioned in the materials and method section |
| The results are not well described in abstract and are too general. Try to be more specific in terms of treatments and also give percent increase or decrease in each observation by the application of treatments. | Corrected |
| In key words, delete "elicitors", "mitigation strategies". Correct "water shortage" as "water stress". Also, include scientific name of canola and the names of plant growth regulators used in this study. | Corrected |
| withhold" or "holds | Thank you Corrected as Holds |
| The statistics related to area and yield of canola are related to which country? Also give the year of these statistics | This is data taken from the Food and Agriculture Organization 2017. Its mentioned in the reference list. |
| With constant efforts of scientists | Thank you, Corrected as Owing to continuous efforts of scientists (originally it was the same sentence but rephrased because of palegrism) |
| Also is this the only reason for low yield of canola in Pakistan? | Elaborated the sentence as Pakistan facing the low yield of canola somewhat due to the poor management of macro- and micronutrient |
| Rewrite "environmental stress alone and in combinations of multiple stresses, such as heat and drought" | Thank you! Sentence is rephrased as “different environmental stresses, particularly heat and drought” |
| Which physiological and biochemical mechanisms are affected by loss in turgor? Briefly explain here. Also check the grammar of this sentence | Chlorophyll contents, oil contents, antioxidants pool are most affected physiological and biochemical attributes  Can’t figure out about the sentence that pointed by the reviewer |
| "The water deficit condition reduced the chlorophyll destruction, photochemical system disorder and stomatal closure"? Recheck this sentence. | Rechecked and improved it as “ The water deficit condition predominates with the reduced concentration of intracellular CO_2_, chlorophyll destruction, photochemical system disorder and stomatal closure” |
| Is it a physiological or biochemical response | Thanks for the correction, it is biochemical against stress |
| The plants showed......" as "The plants respond to drought........" | The sentence is improved as “The plants respond to drought by enhancing osmolytes (proline), antioxidant activities i(POX. PPO and SOD) in plants” |
| Correct as "Nitrogen plays a pivotal role....". Use "N" for "nitrogen" at first mention and follow this abbreviation throughout the manuscript. | Thank you! Corrected in the whole manuscript |
| decrease" instead of "sway | Thank you, the same word was replaced by sway because of palegrism issue. However, accepted |
| This lead to improve nutrient uptake and reduce impact of drought in crops | Thank you! “It improves nutrient uptake and reduces the damaging effects of drought in crops” |
| but current world situations required some immediate response methods for the growing demands of food and feed | Thank you!, rephrased as “ but the current demands of food and feed required some immediate response methods to address food security and hidden hunger” |
| The hypothesis and aims of the study should be given in the introduction | Thank you! Hypothesis/aim given in the introduction”, this manuscript comprised to gauge the morphological, physiological and biochemical rejoinders of canola to assimilate fertilizers management and PGRs with limited supply of water”. |
| Correct as "Two year field experiments were | Thank you! corrected |
| in reaction to fertilization under excessive rate of water evaporation" | “Reaction” replaced by “respond” |
| Also, rate of evaporation or drought stress was one of the main factors of this study? |  |
| The treatment details should be either given in a table or as a paragraph. | Thank you! Given as tabular form |
| GA and IAA should be defined at first mention | Thank you! Mentioned at first place in the “abstract” |
| Are these the recommended NPK rates for canola? | A series of field trials was conducted to optimize the level of these mentioned fertilizers and this data is already under consideration of publication in peer review journal. |
| What was the reason for the selection of these NPK application rates? | Well a very good point! The sole fact of these nutrient rich fertilizers has been described in the introduction section to highlight its significance. In Materials and Methods section, only methods and mode of their application have been described in detail. |
| Were all the fertilizers including N applied at the time of sowing? | NPK was applied at the time of sowing while PGRs were applied exogenously at the time of flowering having tween 20 as surfactantkl |
| Which canola cultivar was used in this study. | Its mentioned at line 105 |
| The data about the soil properties as well as the climatological data should be the part of M&M instead of results | The data about the soil properties added in the Materials and methods section |
| How did the authors apply this small amount of IAA and GA to the plants in the field. At which canola growth stage were the foliar treatments carried out? | Solution of IAA and GA_3_ was prepared in their respective solvent along with tween 20 as surfactant. |
| Did they use any surfactant for foliar treatments? | Yes, Tween 20. |
| Give the instrumental settings for the measurement of stomatal conductance. | Thank you! Added |
| How did the authors manage the different irrigation regimes? |  |
| Line 177: Correct 25oC. Check throughput the manuscript. | Thank you! Corrected  Line 205 |
| Line 176: What was the pH of the phosphate buffer used for extraction? | 7.8 line 192 |
| Line 180: Correct as "Units/g FW". Needs to be consistent about the use Units/g OR Units g-1 throughout the manuscript | Thank you! Made it uniform as “Units g^-1^”  line 204 |
| Line 181: Correct as "interval of 30 | Thank you! Corrected  Line 198 |
| Line 185: "affected" instead of "effected". Also correct as "nitroblue tetrazolium" | Thank you! Corrected line 201 |
| Line 189: Avoid starting as sentence with a number i.e. 0.5 here | Its approximately sign that make start of sentence line 205 |
| Made it uniform Kg/ha as | kg ha^-1^ |
| In Table 3, correct the units for grain yield. | Thank you corrected |
| Also, the discussion should include how the observed increase in yield and quality attributes is linked to various physiological and biochemical responses under drought stress. | Added |
